# Phylogenetic meta-analysis of persistent SARS-CoV-2 infections in immunocompromised individuals highlights the challenges of robust evolutionary rate estimation caused by low genetic diversity

**DOI:** 10.1093/ve/veaf065

**Published:** 2025-08-30

**Authors:** Sanni Översti, Emily Gaul, Björn-Erik Ole Jensen, Denise Kühnert

**Affiliations:** Transmission, Infection, Diversification and Evolution Group (tide), Max Planck Institute of Geoanthropology, Kahlaische Straße 10, 07745 Jena, Germany; Department of Archaeogenetics, Max Planck Institute for Evolutionary Anthropology, Deutscher Platz 6, 04103 Leipzig, Germany; Transmission, Infection, Diversification and Evolution Group (tide), Max Planck Institute of Geoanthropology, Kahlaische Straße 10, 07745 Jena, Germany; Institute for Archaeological Sciences, Department of Geosciences, Eberhard Karls University of Tübingen, Rümelinstraße 23, 72070 Tübingen, Germany; Department of Gastroenterology, Hepatology and Infectious Diseases, Medical Faculty and University Hospital Düsseldorf, Heinrich Heine University Düsseldorf, Moorenstraße 5, 40225 Düsseldorf, Germany; Transmission, Infection, Diversification and Evolution Group (tide), Max Planck Institute of Geoanthropology, Kahlaische Straße 10, 07745 Jena, Germany; Department of Archaeogenetics, Max Planck Institute for Evolutionary Anthropology, Deutscher Platz 6, 04103 Leipzig, Germany

**Keywords:** chronic, COVID-19, immunocompromised, phylogenetic, molecular dating, evolutionary rate, intrahost, within-host

## Abstract

Time-stamped genomic sequences from rapidly evolving pathogens can be used to estimate the rates of evolution through molecular tip-dating. The validity of this approach, however, depends on whether detectable levels of genetic variation have accumulated over the given sampling interval, generating a temporal signal. Moreover, molecular dating methods have demonstrated varying degrees of systematic biases under different biologically realistic scenarios, such as the presence of phylo-temporal clustering. Persistent SARS-CoV-2 infections in immunocompromised individuals have been linked to accelerated intrahost molecular rates compared to those of global lineages, facilitating the emergence of novel viral lineages. Yet, studies reporting elevated rates lack assessment of data properties, such as evaluation of temporal signal and comparison of multiple methods of inference, both crucial for robust rate estimation. In this study, we applied a range of molecular dating approaches to reassess the rate of SARS-CoV-2 intrahost evolution in immunocompromised individuals using publicly available datasets. Our findings suggest that even during long-term infections, the limited number of genetic changes accumulating may pose a challenge for robust inference of within-host evolutionary rates, particularly when relying on consensus sequences and when datasets are small or unevenly sampled. Moreover, our results highlight that when certain methodological limitations are overlooked, evolutionary rates can be significantly overestimated. In general, our findings demonstrate that estimating within-host evolutionary rates is a challenging question necessitating thorough assessment of data quality, careful selection of appropriate methods, and cautious interpretation of the resulting estimates. Whereas our phylogenetic analyses of viral consensus sequences provide no evidence of elevated evolutionary rates across the complete genome during chronic SARS-CoV-2 infection, prolonged viral shedding may nevertheless promote the emergence of new viral variants in immunocompromised individuals.

## Introduction

Molecular dating postulates that differences between two sequences are directly proportional to the time elapsed since they diverged (‘strict molecular clock’, Zuckerkandl and Pauling 1962), hence allowing an estimation of the timing of evolutionary events. Calibration of a molecular clock with independent temporal information is required to convert relative divergence times of a phylogenetic tree into absolute timescales. For serially sampled data sets, including those generated for rapidly evolving pathogens such as severe acute respiratory syndrome coronavirus 2 (SARS-CoV-2), trees can be calibrated using the sampling times of genetic sequences (Li *et al*. 1988, Rambaut 2000) (for review, see Rieux and Balloux 2016).

Whilst time-stamped genealogies have become fundamental for understanding pathogen evolution, the accuracy of estimated evolutionary rates substantially influences the reliability of inferred timescales (for definitions and discussion of different rates of evolution, see Ho and Larson 2006; Ho *et al.* 2011a). As a result, a large range of evolutionary models and methods have been developed, with key distinctions between different methodologies relying on whether the method accommodates phylogenetic uncertainty and if rate heterogeneity amongst lineages can be modelled. In the simplest approach, a linear regression is fitted between sampling dates and corresponding root-to-tip (RTT) genetic distances (Gojobori *et al*. 1990, Drummond *et al*. 2003). In spite of RTT regression analysis being extensively used, its assumptions of statistical independence of the sequences and rate homogeneity amongst lineages can be considered as substantial limitations (Rambaut *et al*. 2016; Rieux and Balloux 2016; Duchene *et al*. 2020a). Alternatively, distance-based approaches, such as least-squares dating (LSD, To *et al*. 2016) and TreeDater (Volz and Frost 2017), providing estimations of evolutionary rates determined by maximizing the likelihood of the rooted phylogeny, have been developed to account for the shared ancestry of the samples. Moreover, these methods have been demonstrated to be somewhat robust to rate heterogeneity (LSD) or to explicitly account for branch-specific evolutionary rates (TreeDater). In contrast to these distance-based methods, probabilistic models implemented in a Bayesian framework can be used for joint estimation of phylogenetic tree topology and evolutionary rates (for an introduction on Bayesian phylogenetic analysis, see, for example, (Huelsenbeck *et al*. 2001; Nascimento and Yang 2017)). Due to their broad applicability, Bayesian phylogenetic methods, such as BEAST2 (Bouckaert *et al*. 2019) and RevBayes (Höhna *et al*. 2016), have become widely utilized for molecular dating. In addition to tree uncertainty, these methods can accommodate complex demographic and evolutionary models, such as an uncorrelated relaxed clock model where the rate associated with each branch is independently drawn from a shared underlying distribution (Drummond *et al*. 2006). More recently, an uncorrelated rate model incorporating the additive nature of molecular data has been developed to better represent mutation rates, considered particularly relevant for dating pathogens, as their phylogenies often contain numerous short branches resulting from intensive sampling over brief time periods (Didelot *et al*. 2021). Consequently, additive relaxed clock models have been implemented in both distance-based frameworks and within joint Bayesian inference of dated phylogenies (Didelot *et al*. 2021).

Irrespective of the phylogenetic approach chosen, a prerequisite for molecular dating analysis of tip-calibrated phylogenies is that genetic changes can be considered to have accumulated rapidly enough relative to the available range of sequence sampling times. If measurable levels of genetic variation have accumulated over a given sampling interval, the population is considered to be ‘measurably evolving’ (Drummond *et al*. 2003). Since insufficient temporal signal might lead to biased estimates of rates and timescales, determining the strength of the temporal signal of heterochronously sampled data is an essential step prior to the estimation of evolutionary rates (Firth *et al*. 2010). In its simplest form, an adequate temporal signal can be considered a positive correlation between sequence sampling times and their corresponding RTT distances (see, for example, Rieux and Balloux 2016). However, since RTT can be viewed as a qualitative method that only provides visual evidence for a sufficient temporal signal (Rambaut *et al*. 2016), more sophisticated approaches, such as the ‘date-randomization test’ (DRT, Ramsden et al. 2008) and ‘Bayesian Evaluation of Temporal Signal’ (BETS, Duchene *et al*. 2020b), have been developed.

Since the onset of the coronavirus disease 2019 (COVID-19) pandemic, tip-calibrated phylogenies have been exploited extensively to gain insights into the origin and spread of SARS-CoV-2 (for review, see Attwood *et al*. 2022). Studies have indicated episodic increases in evolutionary rates within the global SARS-CoV-2 phylogeny, suggesting that these bursts may have contributed to the emergence of new variants, including variants of concern (VOCs) (Hill *et al*. 2022, Neher 2022, Lythgoe *et al*. 2023, Tay *et al*. 2023). Chronic infections are considered a plausible cause for these temporary increases (Hill *et al*. 2022, Neher 2022, Lythgoe *et al*. 2023, Tay *et al*. 2023), as extended viral shedding may create favourable conditions for intrahost evolution (Kang *et al*. 2020, Zapor 2020). Observations that immunocompromised individuals are at greater risk for prolonged infections (for references, see Table 1) support the ‘Chronic Infection Hypothesis’, which proposes that long-term infections in these patients contribute to SARS-CoV-2 evolution and may be a source of VOCs (Chaguza *et al*. 2023). Consistent with this hypothesis, multiple studies have reported up to two-fold higher molecular rates of SARS-CoV-2 evolution within immunocompromised individuals when accounting for the whole SARS-CoV-2 genome (Choi *et al*. 2020, Borges *et al*. 2021, Ciuffreda *et al*. 2021, Karim *et al*. 2021, Hettle *et al*. 2022, Brandolini *et al*. 2023, Chaguza *et al*. 2023, Stanevich *et al*. 2023, Marques *et al*. 2024, Sigal *et al*. 2024). Most commonly, the reported rates are determined by directly calculating the number of mutations accumulated (Borges *et al*. 2021, Ciuffreda *et al*. 2021, Sonnleitner *et al*. 2022) or through root-to-tip regression analysis (Chaguza *et al*. 2023, Stanevich *et al*. 2023, Marques *et al*. 2024). The former may result in an overestimation of the number of changes due to the general assumption of changes accumulating over time in a single viral lineage, contradicting the observations of within-host SARS-CoV-2 viral populations frequently comprising a collection of genetically closely related lineages, i.e. coexisting quasispecies (Kemp *et al*. 2021, Pérez-Lago *et al*. 2021, Harari *et al*. 2022, Brandolini *et al*. 2023, Chaguza *et al*. 2023). The latter, in contrast, is unsuitable for molecular dating because the data points for the regression are not phylogenetically independent due to shared ancestry within the phylogeny (Rambaut *et al*. 2016; Duchene *et al*. 2020a; Neher 2022). Consequently, mutations occurring in the deeper branches contribute to multiple root-to-tip distances. Moreover, these previous studies have not assessed the strength of the temporal signal in within-host datasets, nor have they compared molecular dating methods or examined the extent to which these methods can be reliably used.

**Table 1:** Overview of datasets included in this study.

**Dataset**	**Number of sequences included in the analysis**	**Sampling window (days)**	**Nextstrain clade/Pango lineage** ^**a**^ **/WHO VOC status**	**Individual’s underlying clinical condition** ^**b**^	**Reference**
Baang-pt-1	8	99	20A/B.1.576/–	B-cell neoplasm	(Baang *et al*. 2021)
Brandolini-pt-1	8	86	21J/AY.122/Delta	B-cell neoplasm	(Brandolini *et al*. 2023)
Caccuri-pt-1	12	222	20B/B.1.1/–	B-cell neoplasm	(Caccuri *et al*. 2022)
Chaguza-pt-1	30	392	20A/B.1.517/–	B-cell neoplasm	(Chaguza *et al*. 2023)
Choi-pt-1	9	134	20A/B.1.576/–	Rheumatological/ autoimmune disease	(Choi *et al*. 2020)
Ciuffreda-pt-1	15	129	19B/A.2/–	PID	(Ciuffreda *et al*. 2021)
Gandhi-pt-1	15	141	20A/B.1.576/–	B-cell neoplasm	(Gandhi *et al*. 2022)
Halfmann-pt-1	12	373	20G/B.1.2/–	B-cell neoplasm and PID	(Halfmann *et al*. 2023)
Harari-pt-5	9	75	20B/B.1.1.50/–	B-cell neoplasm	(Harari *et al*. 2022)
Huygens-pt-2	13	160	21K/BA.1.1/Omicron	B-cell neoplasm	(Huygens *et al*. 2023)
Jensen-pt-2	8	22	20B/B.1.1/–	HIV/AIDS	(Jensen *et al*. 2021)
Kemp-pt-1	16	100	20D/B.1.1.1/–	B-cell neoplasm	(Kemp *et al*. 2021)
Khatamzas-pt-1	21	149	20B/B.1.1/–	B-cell neoplasm	(Khatamzas *et al*. 2022)
Lee-pt-11	11	64	20A/B.1/–	B-cell neoplasm	(Lee *et al*. 2022)
Lee-pt-4	8	342	20A/B.1.576/–	B-cell neoplasm	(Lee *et al*. 2022)
Li-pt-1	11	140	19A/B^c^/–	Multiple conditions	(Li *et al*. 2021)
Lynch-pt-1	8	77	20B/B.1.1/–	B-cell neoplasm	(Lynch *et al*. 2021)
Pérez-Lago-pt-1	9	123	19A/B/–	B-cell neoplasm	(Pérez-Lago *et al*. 2021)
Pérez-Lago-pt-2	10	117	20A/B.1/–	B-cell neoplasm	(Pérez-Lago *et al*. 2021)
Riddell-pt-2	9	111	20I/B.1.1.7/Alpha	B-cell neoplasm and HIV/AIDS	(Riddell *et al*. 2022)
Riddell-pt-3	15	255	20I/B.1.1.7/Alpha	HIV/AIDS	(Riddell *et al*. 2022)
Rockett-pt-2	8	31	21J/AY.39.1.2/Delta	PID	(Rockett *et al*. 2022)
Rockett-pt-4	8	40	21J/AY.39.1.3/Delta	Myeolodysplastic syndrome/ myeloproliferative disorder	(Rockett *et al*. 2022)
Rockett-pt-8	12	34	21J/AY.39.1/Delta	B-cell neoplasm and multiple other conditions	(Rockett *et al*. 2022)
Sonnleitner-pt-1	10	98	20B/B.1.1.232/–	B-cell neoplasm	(Sonnleitner *et al*. 2022)
Weigang-pt-1	9	140	20B/B.1.1/–	Multiple conditions	(Weigang *et al*. 2021)

a Defined with Nextclade v2.14.1.

b For details, see Supplementary Table S2.

c For details, see Supplementary Table S1.

In this study, we re-evaluated the rate of intrahost molecular evolution of SARS-CoV-2 in 26 previously published cases of chronic infection in immunocompromised individuals, using a phylogenetic framework. We assessed key data properties, including genetic diversity and the strength of the temporal signal, to better understand their impact on the reliability of inferred intrahost evolutionary rates. Following common practice for both inter- and intrahost rate estimation, we utilized consensus sequences to infer average evolutionary rates across the entire viral genome and compared four widely used phylogenetic approaches whilst simultaneously evaluating their applicability and robustness. Our findings highlight a fundamental biological limitation for reliably inferring within-host evolutionary rates in a phylogenetic context: even during prolonged infections, the accumulation of genetic changes is often limited, which constrains the signal necessary for robust rate estimation. Furthermore, our systematic meta-analysis did not consistently reproduce previously reported elevated intrahost evolutionary rates, demonstrating that the estimates are highly dependent on both the molecular dating method and dataset characteristics. More broadly, our results contribute to the ongoing research on the complexity of intrahost SARS-CoV-2 evolution by exemplifying a notable variability in evolutionary rates amongst chronically infected individuals. Whilst our findings provide no evidence of accelerated intrahost viral evolution at the consensus sequence level, prolonged viral shedding together with the relapsing viral load dynamics may nevertheless promote the emergence of novel viral variants, such as VOCs.

## Materials & methods

### Data collection

**Figure 1:**
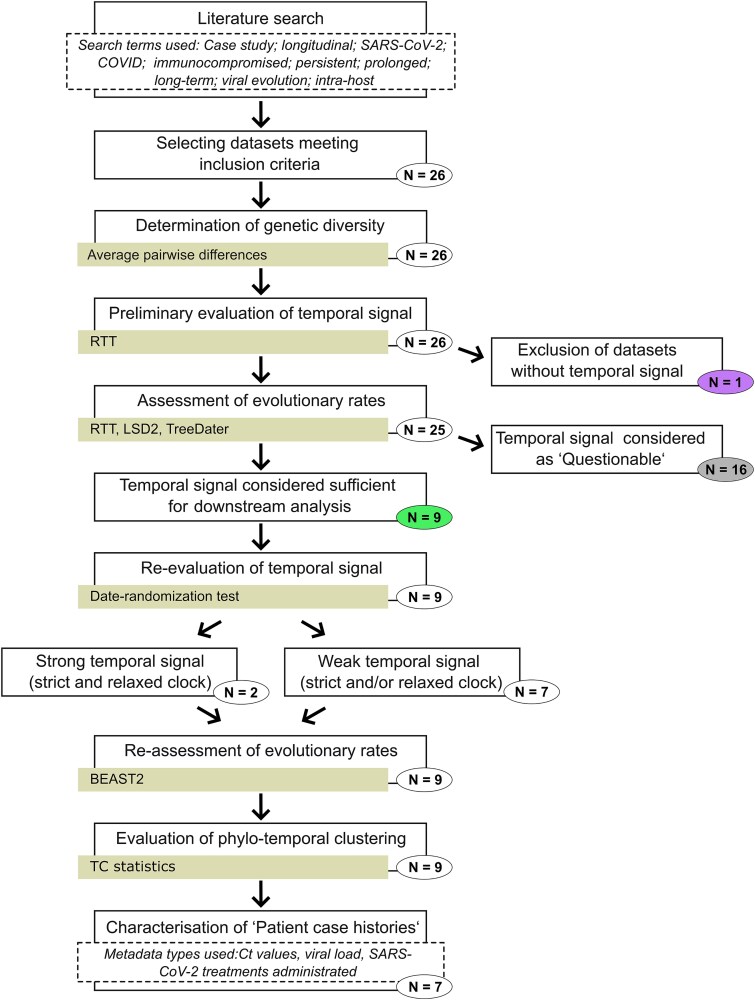
Schematic overview of the workflow. Number of datasets included in each step is given within the circles. Colouring of the number of datasets corresponds to Fig. 2 (purple circle = no temporal signal, grey circle = questionable temporal signal, green circle = sufficient temporal signal). Software/Method or statistics used are indicated with yellow boxes. Additional information is provided in boxes outlined with dashed lines.

A schematic overview of the workflow used in this study is presented in Fig. 1. All data used within this study were obtained through a literature search conducted between 15 August 2022 and 15 March 2023, according to the search terms: Case study; longitudinal; SARS-CoV-2; COVID; immunocompromised; persistent; prolonged; viral evolution; intrahost; and long-term. The resulting dataset of 1029 longitudinally sampled consensus sequences from 255 individuals and 53 publications was then filtered according to the following criteria: (i) given evidence within the original publication of the immunocompromised status of the individual, (ii) confirmation that the infection was the result of a single, long-term infection, i.e. excluding multiple consecutive infections, or a superinfection, and (iii) that at least eight sequences with unique collection dates were available from the individual, with the aim of minimizing phylogenetic uncertainty and thus increasing the precision of parameter estimates. We furthermore followed the procedure presented in Harari *et al*. (2022) and considered an individual to have a chronic SARS-CoV-2 infection if there was evidence of persistent viral shedding for a period of at least 20 days. The removal of all individuals not fulfilling these criteria resulted in a final dataset of 323 consensus sequences from 26 individuals and 21 publications. For the dataset obtained from Lynch *et al*. (2021), the last sample (EPI_ISL_2484152, 2020-07-08) was excluded from all the analyses since, in the original publication, authors suspected a superinfection with a second strain of the virus.

In parallel to sequence data collection, clinical metadata obtained from the original publications or *via* correspondence with the authors are provided within s S1–S6. For consistency, all datasets were renamed according to the first author of the source publication, followed by ‘pt’ and the patient number. This labelling is used throughout the manuscript. Sequence identifiers were renamed according to the day of collection, where, in each case, ‘day 0’ represented the earliest sequence available for the individual. In some instances, multiple samples were collected on the same day, representing different specimen types (e.g. Baang-pt-1_22a and Baang-pt-1_22b). In such cases, only one sample was considered for a given collection date and preference was given to respiratory tract samples, since within-host populations from different tissue types have been shown to be genetically highly distinctive (Wang *et al*. 2021). Pango lineages (Rambaut *et al*. 2020) were obtained from original publications and were further confirmed with Nextclade v2.14.1 (Aksamentov *et al*. 2021).

### Sequence data quality control and assessment of within-dataset genetic diversity

To mitigate the impact of sequencing errors associated with specific laboratories or technologies (De Maio *et al*. 2020, Turakhia *et al*. 2020), we masked all known problematic sites, as suggested in De Maio *et al*. 2020. We furthermore determined the absolute numbers of missing characters (N) and ambiguous characters (R, Y, K, M, S, W, B, D, H, and V) for each sequence with BioEdit (Hall 1999). Sequences were aligned to the SARS-CoV-2 reference genome (Wuhan-Hu-1, NC_045512.2) in MAFFT v7.475 (Katoh and Standley 2013) with the --keeplength option. Within each dataset, the mean number of pairwise differences was determined with MEGA 11 (Tamura *et al*. 2021). Distances were estimated by calculating the absolute number of differences by assuming uniform rates amongst sites and treating gaps and missing data as pairwise deletion, meaning that sites containing missing data and/or alignment gaps are removed for each pair of sequences individually. As a variance estimation method, we ran a nonparametric bootstrap with 100 replications.

### Evolutionary rate estimates—RTT, LSD2, and TreeDater

A shared property of distance-based methods used within this study is that they rely upon a user-specified substitution tree for which the optimal root position is estimated based on software-specific algorithms. For all distance-based methods, the maximum likelihood substitution tree inferred with IQ-Tree v2.1.2 (Minh *et al*. 2020) was provided as an input. The best-fit substitution model was simultaneously estimated with ModelFinder (Kalyaanamoorthy *et al*. 2017) (iqtree2 -s input.fasta -m MFP). As ambiguous characters have an influence on the estimation of branch lengths and thus on evolutionary rates, sequence positions exhibiting ambiguous characters were treated as informative sites, as implemented in IQ-Tree by default.

For each dataset, we assessed the strength of temporal signal with root-to-tip linear regression with the R package BactDating (Didelot *et al*. 2018). The significance of the temporal signal was assessed through random permutations of sampling dates, following the approach implemented in BactDating with 10 000 permutations. This procedure compares estimates obtained with the correct sampling dates to those inferred when all sequences are assigned the same sampling date. At this point, the temporal signal was considered sufficient for further analysis if the *P*-value was <0.05. Subsequently, for datasets with an RTT-confirmed temporal signal, evolutionary rate estimates were assessed with three methods: BactDating (RTT), Least-Squares Dating (LSD2) method integrated in IQ-TREE v2.1.2, and TreeDater. Time trees were inferred by using sampling dates as tip dates, and the root position was estimated as a part of the analyses. For LSD2, the best-fit substitution model was estimated with ModelFinder, as described previously. Regarding the tree, we chose to use two different approaches: Within the first approach, we followed the LSD2 default values and collapsed all internal branches having a branch length < 1.67e-05 (=0.5/sequence length). Within the second approach, none of the branches were collapsed, implying that null branches were allowed. For the output, tree branch lengths were resampled in total 100 times to determine the confidence intervals (with *--date-ci* option). With TreeDater, the molecular rates were determined by assuming a strict and relaxed clock. For both, confidence intervals for the rate estimates were estimated with a parametric bootstrap with 100 replicates. Based on the results obtained from LSD2 and TreeDater, the strength of the temporal signal of each dataset was re-evaluated: If LSD2 and/or TreeDater analysis yielded error messages indicating a poor temporal signal, the temporal signal for the dataset under scrutiny was considered as ‘Questionable’.

### Evolutionary rate estimates—BEAST2

For the datasets passing the re-evaluation of the temporal signal, the evolutionary rates were additionally determined with BEAST v.2.6.7. Evolutionary rates were inferred with strict and uncorrelated relaxed lognormal clock models by assuming a Bayesian Skyline Plot (BSP) as an underlying tree model (see Supplementary text S2 for details and additional sensitivity analysis). Due to small sample sizes, dimensions for BSP model parameters bPopSize and bGroupSize were set to 3–5, depending on the data set. As a substitution model HKY + Γ was used, assuming four gamma rate categories. Whilst recent work has shown that modelling site heterogeneity with a gamma distribution may introduce systematic bias in branch length estimates, this effect is expected to be minimal for small datasets with fewer than 50 sequences (Ferretti *et al*. 2024), which is considerably larger than the datasets analysed here. For datasets containing ambiguous characters, i.e. Chaguza-pt-1 and Khatamzas-pt-1, these characters were treated as informative sites through option useAmbiquities = ‘true’. As a prior distribution for a strict clock rate parameter (clockRate), a uniform distribution (0,1) was used. The same uniform distribution was originally used also for the relaxed clock rate parameter (ucldMean). However, Markov Chain Monte Carlo (MCMC) chains were not reaching convergence. Therefore, we chose to use a more stringent prior and set normal distribution with a mean of 8.0e-04 (subst./site/year) and a standard deviation of 16.0e-04 (subst./site/year). As an additional sensitivity analysis, we conducted further runs using a Gamma-distributed prior for the relaxed clock rate parameter, specifying a shape parameter $\alpha$ = 8.0 and a rate parameter $\lambda$ = 10^4^. This prior results in a mean rate of 8.0e-04 subst./site/year, with 95% of the probability density spanning from 3.45e-04 to 14.4e-04 subst./site/year. No additional modifications were made to the default prior distributions.

The temporal signal was assessed with a date-randomization test (DRT) implemented in R package TIPDATINGBEAST (Rieux and Khatchikian 2017). For the DRT, for each dataset for both clock models, 20 randomized data sets were generated as recommended in Duchêne *et al*. (2015b). We used two previously proposed criteria to evaluate the strength of the temporal signal: (i) there is no overlap between posterior distributions of true and randomized datasets (Ramsden *et al*. 2009), and (ii) the true mean value is not contained in any of the randomized posterior distributions (Firth *et al*. 2010). The MCMC chain length was set to 10–50 million steps for all MCMC analyses. For the BEAST2 analyses with nonrandomized sampling dates, the posterior distributions of parameters were estimated based on two parallel MCMC chains. After confirming sufficient sampling of each chain (effective sample sizes for each parameter > 200), the samples from two runs were combined after discarding the first 10% of each chain as a burn-in. Maximum clade credibility trees with median node heights were reconstructed with TreeAnnotator by assuming 10% as a burn-in. MCC trees were visualized with FigTree v1.4.4 (http://tree.bio.ed.ac.uk/software/figtree/, last visited 11 November 2024).

### Estimating topological distances

The topological distances between pairs of phylogenetic trees were estimated with the R package TreeDist v.2.6.3 (Smith 2020) (https://zenodo.org/records/3528124, last visited 11 November 2024), which represents an information-based generalized Robinson–Foulds metric that defines the overall similarity between two trees. For each dataset, three comparisons were performed: LSD2 vs. BEAST2 strict clock MCC tree, LSD2 vs. BEAST2 relaxed clock MCC tree, and BEAST2 strict clock MCC tree vs. BEAST2 relaxed clock MCC tree. According to Smith (2020), ‘SharedPhylogeneticInfo’ metrics describes the amount of phylogenetic information in common between two trees, whereas ‘DifferentPhylogeneticInfo’ metrics describes the distance between trees under scrutiny, i.e. how much information is different in the splits of these two trees. When ‘DifferentPhylogeneticInfo’ yielded a value of 0, trees were considered identical. When the score for shared splits exceeded the score for conflicting splits (‘SharedPhylogeneticInfo’ > ‘DifferentPhylogeneticInfo’), two trees were considered to exhibit modest variation in the tree topology. When the score for conflicting splits exceeded the score for shared splits (‘SharedPhylogeneticInfo’ < ‘DifferentPhylogeneticInfo’), trees were considered to exhibit notable variation in the tree topology.

### Evaluating the degree of phylo-temporal clustering

The degree of temporal clustering was estimated by calculating temporal clustering (TC) statistics (Gray *et al*. 2011) implemented in R package PhyloTempo (Norström *et al*. 2012). As an input, we used the same unrooted substitution trees generated with IQ-Tree, which we also used as input for BactDating, LSD2, and TreeDater. For each dataset, the TC score was defined with three independent runs by setting the number of randomizations to 500. In case these three separate analyses produced highly divergent TC score estimates, we considered the degree of temporal clustering as unresolved.

### Test of positive selection

The presence of positive selection was evaluated through a codon-based *Z*-test of selection averaging over all sequence pairs within the dataset for nine of the datasets. As a null hypothesis, we assumed strict neutrality (*d*_N_ = *d*_S_) and as an alternative hypothesis, positive selection (*d*_N_ > *d*_S_). All calculations were conducted with MEGA 11 (Tamura *et al*. 2021) by using the Pamilo–Bianchi–Li method by assuming a pairwise deletion, as described previously.

## Results

### Data collection and quality assessment

**Figure 2:**
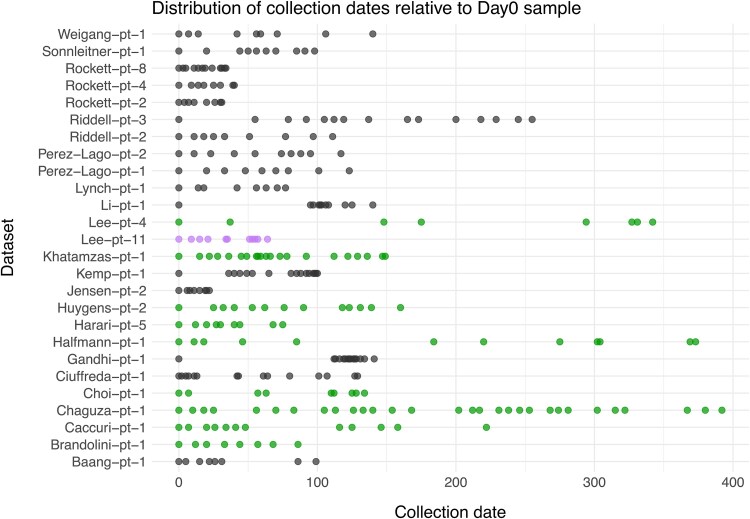
Temporal distribution of sample collection points. Collection dates are given relative to the first sample of each dataset (Day 0), whereas in Supplementary Fig. S1, collection dates are represented in calendar years. Datasets are coded according to their temporal signal: purple circles indicate datasets with no temporal signal, dark-grey circles indicate a poor (‘Questionable’) temporal signal, and green circles denote datasets with a sufficient temporal signal (evaluated based on analysis with RTT, LSD2, and TreeDater).

Data collection resulted in the identification of 26 individuals meeting all the inclusion criteria. Clinical metadata, sequence accession information, and details of the sequences are reported within Supplementary Tables S1–S8. The samples of the final dataset derived primarily from the respiratory tract including nasopharyngeal, oropharyngeal, combined nasopharyngeal/oropharyngeal, as well as less commonly sputum, bronchoalveolar lavage, and tracheal aspirate specimen types. The number of sequences per dataset varied from 8 to 30 sequences (Table 1). The sampling windows, i.e. the days between the first and last sequence sampling point for each dataset, ranged from 22 days (Jensen-pt-2) to 392 days (Chaguza-pt-1) (Table 1, Fig. 2). Collection date information was available in calendar units for 22 datasets, and altogether, these covered a time period from February 2020 to June 2022 (Supplementary Fig. S1).

To minimize the possibility of sequences being recombinants of two different viral variants, we further verified that all sequences within a dataset represented the same Pango lineage. Datasets represented in total 16 different Pango lineages, and furthermore, seven of the individuals carried lineages identified as variants of concern (Table 1). The assignment of Pango lineages to Li-pt-1 sample series suggested that samples reflected distinct lineages (Supplementary Table S1). However, since the original paper by (Li *et al*. 2021) regarded strong sequence similarity as evidence against reinfection, we decided to include the dataset in the subsequent study.

Eighteen of the individuals were receiving treatment for B-cell neoplasm (including B-cell lymphoma and B-cell leukaemia), three each for primary immunodeficiency (PID) and for HIV/AIDS, one for myelodysplastic syndrome/myeloproliferative disorder, and one for rheumatological/autoimmune disease, as well as three individuals with other forms of immunodeficiency (Table 1). Some of the individuals had more than one disease associated with immunodeficiency (Supplementary Table S2). Due to highly unequal representation of distinct underlying clinical condition categories, the potential differences in how they may influence the intrahost evolution of SARS-CoV-2 were not further explored nor discussed within this study.

### Assessment of genetic diversity amongst datasets and temporal signal with RTT

Whereas approximately half of the datasets displayed low levels of genetic diversity with observed mean pairwise differences being <5.0, for some, the differences were notably higher, yielding mean values above 10.0 (Supplementary Table S9). As we detected genetic changes within all datasets, the strength of the temporal signal was first assessed with the regression of root-to-tip distances and associated permutation test, the former indicating a positive correlation for all datasets (Supplementary Fig. S2). However, the datasets displayed highly variable levels of temporal signal, with *R*^2^ values ranging from 0.23 to 0.99 and *P*-values between *P* < 1.00e-04 and 6.45e-02. The estimates of *R*^2^ = 0.23 and *P* = 6.45e-02 observed for Lee-pt-11 indicated an inadequate temporal signal, and the dataset was excluded from subsequent analyses. Based on the positive correlation between genetic differences and sequence sampling dates, 25 of the datasets included in this study would be suitable for phylogenetic molecular clock analysis (Rambaut *et al*. 2016). However, subsequent analyses with LSD2 and TreeDater excluded many of these, showing adequate temporal signal for only nine datasets (Fig. 2). For the remaining 16, a lack of sufficient temporal signal was detected and therefore the temporal signal was considered as ‘Questionable’.

The majority of the datasets for which LSD2 and TreeDater exhibited poor performance displayed rather low genetic diversities (i.e. Baang-pt-1, Jensen-pt-2, Lynch-pt-1, Pérez-Lago-pt-1, Pérez-Lago-pt-2, Riddell-pt-2, Riddell-pt-3, Rocket-pt-2, Rocket-pt-4, Weigang-pt-1) (Supplementary Table S9). For some datasets with higher genetic diversity, the weak temporal signal may be explained by highly skewed temporal distributions of sampling points (i.e. Gandhi-pt-1, Kemp-pt-1, and Li-pt-1) (Fig. 2). Genetic diversities showed positive correlations between sampling windows for datasets with questionable and sufficient temporal signals with correlation coefficient values of *R*^2^ = 0.42 and *R*^2^ = 0.84, respectively (Supplementary Fig. S3). However, for datasets with questionable temporal signal correlation was not statistically significant (*P* = 0.11). This indicates that the duration of infection can explain only some of the observed genetic diversity, meaning that novel mutations have emerged with highly variable patterns across datasets.

### Evolutionary rate estimates—RTT, LSD2, and TreeDater

**Figure 3:**
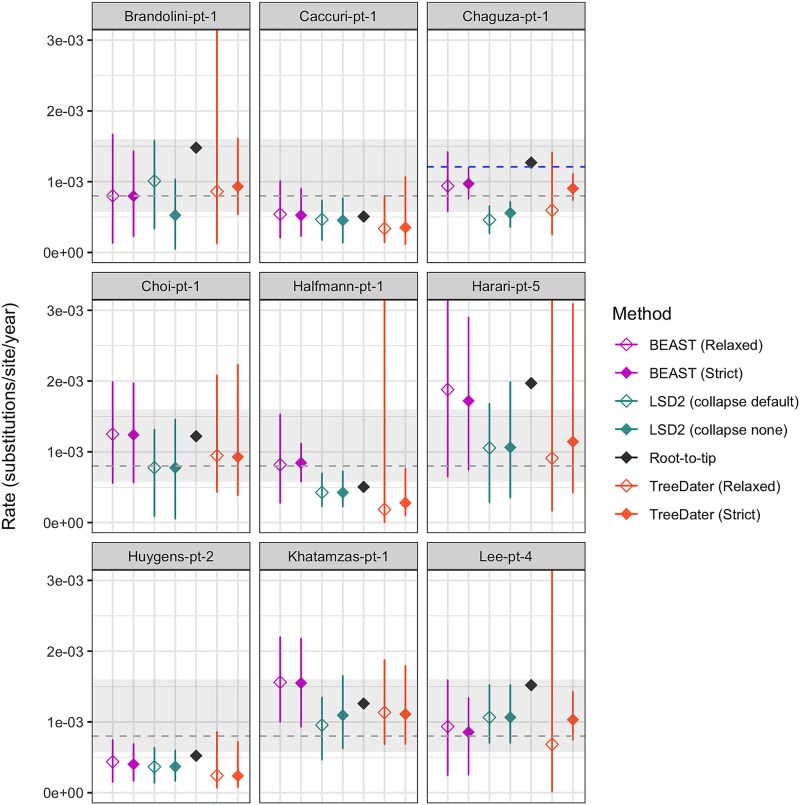
Evolutionary rate estimates determined for nine datasets with sufficient temporal signal (strength of temporal signal defined here based on RTT as well as TreeDater and LSD2 results). For all datasets, rates were determined with following methods: BEAST2 (by assuming relaxed and strict clock models), LSD2 (with and without collapsing short branches), root-to-tip, and TreeDater (by assuming relaxed and strict clock models). In each panel, the *Y* axis denotes the evolutionary rate in subst./site/year. For estimates inferred with BEAST2, diamonds represent median estimates and associated vertical lines correspond to 95% highest posterior density intervals (HPDIs). For RTT only point estimates are represented. For other distance-based methods (i.e. LSD2 and TreeDater), diamonds represent mean estimates and bars illustrate confidence intervals. Grey dashed line represents the commonly used SARS-CoV-2 substitution rate estimate of 8.0e-04 subst./site/year. The grey-shaded area denotes the lowest and highest mean evolutionary rate estimates for SARS-CoV-2 collected from various publications (3e-04–16e-04 subst./site/year, see Supplementary Table S12). For Chaguza-pt-1, a previous estimate of 1.2e-03 subst./site/year is indicated with a blue dashed line.

**Figure 4:**
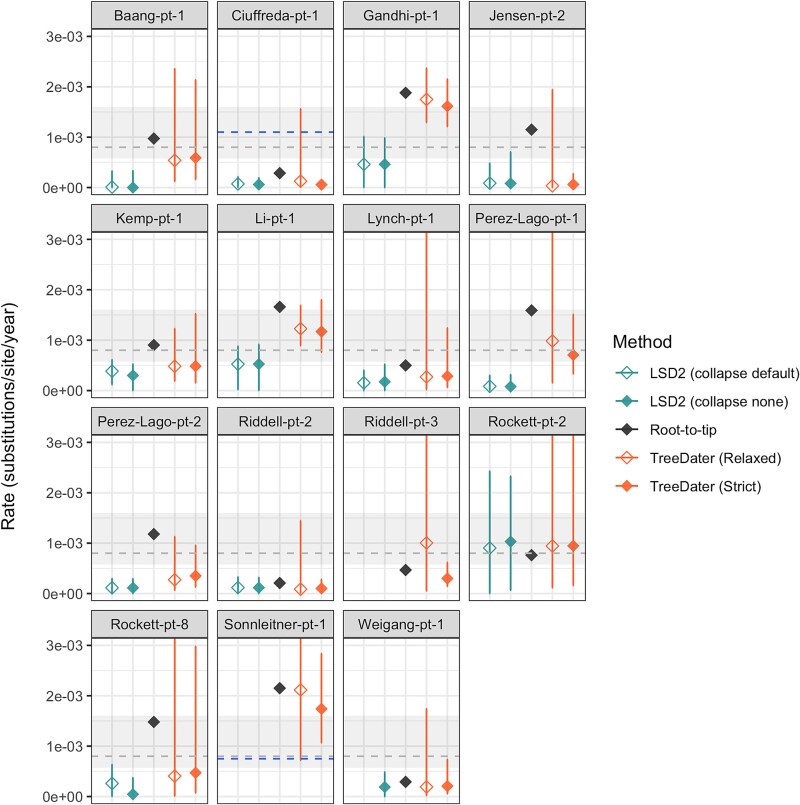
Substitution rate estimates for datasets with ‘Questionable’ temporal signal. For all, rates were determined with following methods: LSD2 (with and without collapsing branches with short lengths), root-to-tip, and TreeDater (by assuming relaxed and strict clock models). In each panel, the *Y* axis denotes the evolutionary rate in subst./site/year. For RTT, only point estimates are represented. For other distance-based methods (i.e. LSD2 and TreeDater), diamonds represent mean estimates and horizontal lines illustrate confidence intervals. Rockett-pt-4 was removed as only RTT was successful (with rate estimate of 9.9e-03 subst./site/year). For Riddell-pt-3 and Sonnleitner-pt-1, evolutionary rate estimates could not be determined with LSD2. Similarly, for Weigang-pt-1 LSD2 analysis with default branch-collapse settings did not produce results. Grey dashed line represents the commonly used SARS-CoV-2 substitution rate estimate of 8.0e-04 subst./site/year. The grey-shaded area denotes the lowest and highest mean evolutionary rate estimates for SARS-CoV-2 collected from various publications (3e-04–16e-04 subst./site/year, see Supplementary table S12). For Ciuffreda-pt-1 and Sonnleitner-pt-1, previously reported estimates of 1.1e–03 and 7.5e-4 subst./site/year, respectively, are indicated with a blue dashed line.

Comparison of evolutionary rates obtained with RTT, LSD2, and TreeDater revealed notable discrepancies across the estimates between different datasets as well as between different methods (Figs 3 and 4). Inconsistencies amongst methods were observed for datasets both with and without an adequate temporal signal. For the nine datasets with sufficient temporal signal, LSD2 and TreeDater yielded comparable mean rate estimates within each dataset (Fig. 3, Supplementary Table S10), yet estimates obtained with RTT were, on average, 1.6–1.9 times higher than those obtained with LSD2 or TreeDater (Supplementary Table S11). A more pronounced pattern of elevated RTT estimates was observed amongst datasets with a ‘Questionable’ temporal signal, where RTT produced estimates that were, on average, 3.1–14.4 times higher than the corresponding point estimates inferred with LSD2 or TreeDater (Supplementary Table S11). The same substitution tree served as input for all three methods, and hence, these differences cannot be attributed to variations in tree reconstruction methods or differences in how methods handle ambiguous sites. Within each dataset, no notable differences were detected between estimates produced with TreeDater by assuming strict or relaxed clock models. Similarly, LSD2 produced highly congruent estimates with and without collapsing the short branches of the tree.

For LSD2, we additionally evaluated the possible impact of an outgroup inclusion and re-inferred rate estimates for trees rooted with the SARS-CoV-2 reference sequence (GenBankID: NC_045512.2). No considerable differences were detected between the estimates reconstructed with and without an outgroup (Supplementary Fig. S4), suggesting that possible topological errors have only a modest impact on the inferred LSD2 rate estimates, if any, as also indicated by To *et al*. (2016). Moreover, in Supplementary text S1, we evaluate the recently proposed method by Sigal *et al*. (2024) for estimating intrahost evolutionary rates using a putative ‘founder sequence’. We furthermore demonstrate the robustness of estimates obtained with LSD2 compared to RTT, both with and without exploiting the ‘founder sequence’ as an outgroup, using Chaguza-pt-1 as a representative sample series (Supplementary text S1 and Supplementary Fig. S5).

We compared the rates obtained within this study with three types of previously published estimates: firstly, with a commonly used point estimate of 8.0e-04 subst./site/year reconstructed based on host-to-host transmission chains (Ghafari *et al*. 2020), secondly, with a range of mean estimates collected from various publications describing evolutionary rates for SARS-CoV-2 host-to-host acute infections (Supplementary Tables S12 and S13), and thirdly, for those datasets for which a within-host rate was estimated in the source publication, this estimate was included in the comparison. This comparison revealed that for six out of nine datasets with sufficient temporal signal, the RTT estimate was higher than the point estimate of 8.0e-04 subst./site/year, whereas only for Harari-pt-5, the RTT estimate of 1.97e-03 subst./site/year exceeded all mean substitution rate estimates obtained from the literature (Fig. 3). Whilst some of the mean estimates from LSD2 or TreeDater analysis were higher than 8.0e-04 subst./site/year, none of them exceeded the collection of mean estimates. However, for Brandolini-pt-1, Halfmann-pt-1, Harari-pt-5, and Lee-pt-4, the widths of the confidence intervals revealed considerable uncertainty in LSD2 and TreeDater estimates. A previous intrahost rate estimate of 1.2e-03 subst./site/year available for Chaguza-pt-1 was obtained with an RTT regression approach and was therefore equal to our RTT estimate.

For the majority of the datasets representing lower degrees of temporal signal, the evolutionary rates obtained in this study were generally in good accordance with published host-to-host estimates (Fig. 4). Amongst these datasets with ‘Questionable’ temporal signal, within-host rates have been previously determined for Ciuffreda-pt-1 and Sonnleitner-pt-1. The previously reported rate for Ciuffreda-pt-1 (Ciuffreda *et al*. 2021) is notably higher than the estimates obtained in this study. Conversely, the previously reported rate for Sonnleitner-pt-1 (Sonnleitner *et al*. 2022), is considerably lower than RTT and TreeDater estimates derived in this study. However, the results should be interpreted cautiously since the genetic diversity and temporal spread of samples may not be sufficient to adequately inform the molecular clock.

### Evolutionary rate estimates—BEAST2

For the nine datasets exhibiting stronger temporal signals, evolutionary rates were also determined with BEAST v.2.6.7. The temporal signal, an essential prerequisite for Bayesian rate estimates (Duchêne *et al*. 2016, Rambaut *et al*. 2016), was additionally assessed with a date-randomization test (DRT) for these nine datasets (Supplementary Figs S6 and S7). By assuming the Ramsden *et al*. (2009) criterion (see Materials & Methods), DRT analysis of a strict clock model displayed strong evidence for sufficient temporal signal in four of the datasets (Caccuri-pt-1, Chaguza-pt1, Halfmann-pt-1, and Khatamzas-pt-1). When assuming the more lenient criterion by Firth *et al*. (2010), datasets Choi-pt-1 and Lee-pt-4 were also included. Considering the uncorrelated relaxed lognormal clock model, a strong temporal signal was observed only for Chaguza-pt-1 (Ramsden *et al*. 2009 criterion) or Chaguza-pt-1 and Khatamzas-pt-1 (Firth *et al*. 2010 criterion). For the rest of the datasets, as the 95% highest posterior density intervals (95% HPDIs) for the randomized datasets were somewhat overlapping with the real rate estimates, the strength of the temporal signal might not be sufficient to infer evolutionary rates with high confidence within a Bayesian framework.

Despite the DRT analysis not indicating a strong temporal signal, particularly when assuming a relaxed clock model, evolutionary rates generated with BEAST2 were compared with estimates retrieved by other methods. For Brandolini-pt-1, Huygens-pt-2, and Lee-pt-4, BEAST2 median estimates were in accordance with mean values obtained with LSD2 and TreeDater, whilst for the rest, the median estimates inferred with BEAST2 were higher. Furthermore, for Caccuri-pt-1, Choi-pt-1, and Khatamzas-pt-1, BEAST2 estimates exceeded the generally high RTT point estimates. Overall, BEAST2 estimates showed less consistency than LSD2 and TreeDater relative to RTT. However, despite BEAST2 producing sporadically higher rates than other methods, similarly to RTT, only Harari-pt-5 displayed a Bayesian median estimate exceeding the literature reference values used. Whereas BEAST2 estimates obtained with strict and relaxed clock models were in good accordance within each dataset, evaluation of the estimated coefficient of rate variation alluded that for none of the nine datasets, the evolutionary rate can be considered strictly constant across branches through time (Supplementary Fig. S8). Furthermore, the sensitivity analyses using a Gamma-distributed prior for the relaxed clock rate parameter produced rate estimates highly consistent with those obtained under the originally applied normally distributed prior (Supplementary Fig. S9). Additionally, rates obtained with alternative coalescent-based tree prior models yielded highly similar estimates across both strict and relaxed molecular clock frameworks (Supplementary Text S2 and Supplementary Fig. S10).

### Evaluating phylogenetic tree topologies and degrees of phylo-temporal clustering

To further examine the possible causes of the evolutionary rate estimate inconsistencies observed principally between BEAST2 vs. LSD2 and TreeDater, we inspected the topologies of SARS-CoV-2 phylogenetic trees. For the majority of the nine datasets, modest split differences were observed between LSD2 and BEAST2 MCC trees, but only for Caccuri-pt-1 and Huygens-pt-2, the score for conflicting splits exceeded the score for shared splits (Supplementary Table S14). Further visual evaluation of the BEAST2 MCC trees revealed a ‘ladder-like’ topology for the majority of the nine datasets (Supplementary Figs S11–S19). This type of tree topology is considered indicative of excessive phylo-temporal clustering (Grenfell *et al*. 2004), known to impact rate estimates (Duchêne *et al*. 2016, Tong *et al*. 2018). Therefore, we further assessed its likely effect by calculating TC score that describes the degree of phylo-temporal clustering. For Chaguza-pt-1, Halfmann-pt-1, Harari-pt-5, and Khatamzas-pt-1, we observed TC scores ranging between ~0.3 and ~0.5 (Supplementary Table S15), indicating a high degree of temporal clustering (Gray *et al*. 2011). For these datasets, evolutionary rate estimates obtained with BEAST2 were notably higher than corresponding estimates produced with LSD2 or TreeDater. For Caccuri-pt-1 and Choi-pt-1, TC scores were <0.1, presumably indicating a lesser degree of phylo-temporal clustering. Whereas for Caccuri-pt-1, similar rate estimates were obtained with all methods, for Choi-pt-1, BEAST2 estimates are greater than LSD2 or TreeDater estimates. However, TC statistics are reported to be sensitive to small sizes below 20 (Gray *et al*. 2011), and thus, a small sample size of nine sequences for Choi-pt-1 might affect its TC score. For the remaining three datasets (Brandolini-pt-1, Huygens-pt-2, and Lee-pt-4), we were not able to resolve the TC score unambiguously (for details, see Materials & Methods).

For Huygens-pt-2, a closer evaluation of MCC tree topologies (Supplementary Fig. S17), revealed a notable substructure of the viral population. Whereas the first sequence for the datasets was obtained on the same day as the reported onset of symptoms, the median estimates for the tree height date 2 months earlier with both clock models (Supplementary Fig. S17). Similar estimates for the most recent common ancestor were obtained with LSD2, and TreeDater yielded even older estimates. Based on this, it is plausible that the individual has been superinfected with two SARS-CoV-2 strains representing the same Pango lineage (BA.1.1), and thus, results for Huygens-pt-2 have been interpreted with caution.

### Test for positive selection

We further evaluated the possibility of continuous selection resulting in ladder-like tree topologies, as suggested by Grenfell *et al*. (2004). Additionally, positive selection is expected to increase the rate of nonsynonymous mutations, whilst the synonymous rate is assumed to remain largely unaffected, resulting in an elevated dN/dS ratio and thus higher evolutionary rates. Amongst nine of the datasets, only Lee-pt-4 showed evidence of positive selection across all functionally important proteins, albeit this was presumably driven by strong positive selection on ORF1ab, which constitutes the vast majority of the SARS-CoV-2 genome (Supplementary Table S16). For four of the datasets, positive selection was detected on the S gene (Brandolini-pt-1, Chaguza-pt-1, Choi-pt-1, and Halfmann-pt-1), but no such signal was detected at the genome-wide level (i.e. considering ORF1ab, S, E, M, and N genes together). For the remaining datasets (Caccuri-pt-1, Harari-pt-5, Huygens-pt-2, and Khatamzas-pt-1), we found no statistically significant evidence of positive selection, either in individual genes or across the genome. This suggests that, across the whole genome, the overall ratio of nonsynonymous to synonymous mutations is not significantly elevated in eight of the nine sample series examined. Consequently, the test of positive selection does not provide additional evidence for accelerated evolutionary rates in these series.

### Patient case histories

**Figure 5:**
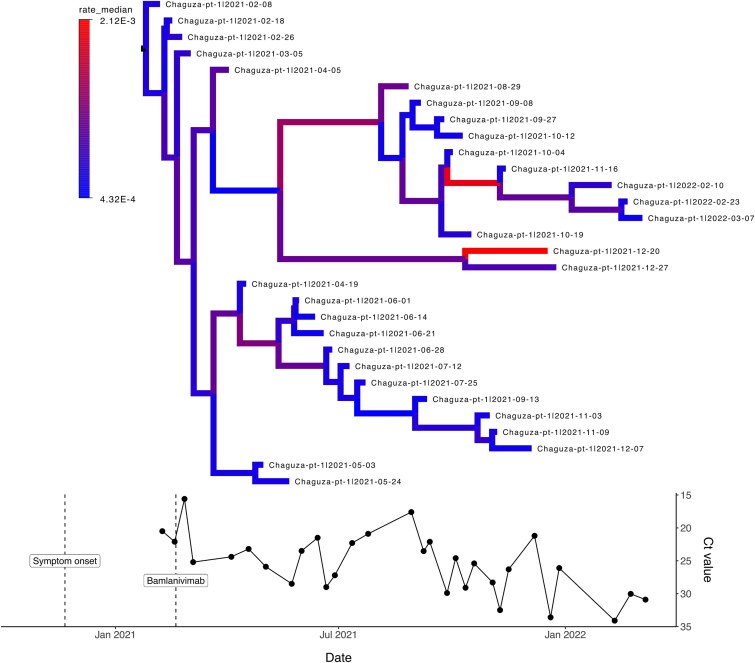
Patient case history for Chaguza-pt-1 patient, with advanced lymphocytic leukaemia and B-cell lymphoma as underlying clinical conditions. Figure describes through time the changes in the evolutionary rates (by assuming an uncorrelated lognormal relaxed clock model), Ct values and SARS-CoV-2 treatments administered within the sampling window. For Chaguza-pt-1 dataset, the first viral sequence was obtained 79 days after the first positive test. Patient was treated with bamlanivimab, which targets spike protein and has a half-time of ~17 days. Colouring of the branches within the phylogenetic tree represents evolutionary rate estimates (in subst./site/year) obtained with BEAST2, lower values indicated with blue and higher rates with red colour. Open circles denote samples for which only Ct values were available, and coloured circles denote samples that were sequenced.

**Figure 6:**
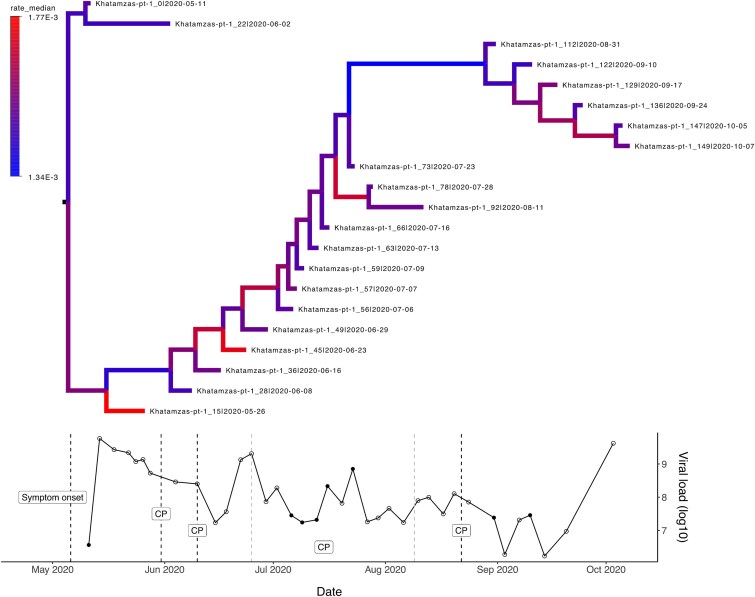
Patient case history for Khatamzas-pt-1 patient, with follicular lymphoma as underlying clinical condition. Figure describes through time the changes in the evolutionary rates (by assuming an uncorrelated lognormal relaxed clock model), viral load on a logarithmic scale, and SARS-CoV-2 treatments administered within the sampling window. For the Khatamzas-pt-1 dataset, the first viral sequence was obtained 5 days after the onset of symptoms. Patient was treated with convalescent plasma (CP) multiple times within the sampling window: on Days 20, 30, 45–90, and 103 after the first sequenced sample (i.e. Day 0). Convalescent plasma targets spike-protein and has a half-time of ~26 days with notable variation. Colouring of the branches within the phylogenetic tree represents evolutionary rate estimates (in subst./site/year) obtained with BEAST2, lower values indicated with blue and higher rates with red colour. For the viral load SARS-CoV-2 RNA copy numbers per millilitre of endotracheal aspirates are presented (Khatamzas *et al*. 2022). Open circles denote samples for which only viral load estimates were available, and coloured circles denote samples that were sequenced.

We obtained evidence of non-clocklike evolution in all nine datasets (Supplementary Fig. S8). Considering this, we were further interested in contrasting the timing of evolutionary rate changes with the temporal fluctuations in the viral load and the timing of SARS-CoV-2 treatments administered. As a proxy for viral load, we used Ct values or direct estimates of viral load, available for seven of the individuals, for which we additionally collected the SARS-CoV-2 treatment information, if any, from the original publications (Supplementary Tables S2 and S4). Changes in evolutionary rates through time were characterized by visualizing BEAST2 MCC trees by assuming an uncorrelated relaxed clock model. However, as the BEAST2 estimates appeared biased towards higher rates, we further evaluated if the observed temporal oscillations in evolutionary rates hold through additional sensitivity analysis (Supplementary text S3 and Supplementary Figs S20–S26). ‘Patient case histories’ for Chaguza-pt-1 and Khatamzas-pt-1, the only datasets for which temporal signal was adequate for relaxed clock analysis, are characterized in Figs 5 and 6, respectively. For the rest of the datasets results are presented in Supplementary Figs S27–S31. These seven individuals displayed numerous different clinical conditions leading to a severely immunosuppressed condition (Table 1). Altogether the datasets covered a lengthy period of time from April 2020 to July 2022 (Supplementary Fig. S1), during which new therapeutics for SARS-CoV-2 infection were developed. As a consequence, a notable variation in the treatment types can be detected amongst individuals, antibody-based treatments targeting the spike protein—i.e. convalescent plasma, bamlanivimab, intravenous immunoglobulin, and sotrovimab—being the most commonly used therapeutic agents. Two of the individuals also received remdesivir with a direct antiviral activity targeting RNA polymerase. Moreover, the half-lives of different treatments vary greatly, ranging from a few hours for remdesivir (Corcione *et al*. 2021, Humeniuk *et al*. 2021) to nearly 7 weeks for sotrovimab (Gupta *et al*. 2021) (https://www.ema.europa.eu/en/medicines/human/EPAR/xevudy, last visited 11 November 2024). Convoluted cycling patterns of viral load were found in all seven individuals, complicating a systematic comparison even further (Supplementary Fig. S32). Whilst a visual examination revealed no strong evidence of temporal correspondences between molecular rate variation, viral loads, and the various SARS-CoV-2 treatments administered, further statistical testing was limited by the small sample sizes considered.

## Discussion

### Limitations of the data

The robustness of molecular dating inferences primarily relies on within-sample genetic diversity, evolutionary rate, the range of sampling times, and the number of sequences (Duchene *et al*. 2020b). Most datasets included in this study did not meet these criteria due to relatively small numbers of sequences, relatively narrow sampling windows, or both. A reliable inference of intrahost evolutionary rate estimates was feasible only for a subset of datasets analysed, proposing that in many sample series, even prolonged infections failed to produce a measurable evolutionary signal. Our findings suggest that the number of accrued mutations over the course of persistent SARS-CoV-2 infections, spanning from several weeks to months, does not necessarily generate sufficient phylogenetic signal, potentially leading to biased and highly uncertain rate estimates. Overall, our results highlight the critical importance of ensuring the data properties, such as the genetic diversity and the strength of temporal signal, before inferring intrahost rate estimates within a phylogenetic framework—an essential aspect that has been overlooked in several previous studies reporting accelerated within-host rates (Chaguza *et al*. 2023, Stanevich *et al*. 2023, Harari *et al*. 2024, Marques *et al*. 2024).

Whilst our data inclusion criteria of at least eight sequences were arbitrarily chosen, our findings in principle underscore the importance of sample size. Only the two datasets with the largest sample sizes—Chaguza-pt-1 and Khatamzas-pt-1, containing 30 and 21 samples, respectively—exhibited a sufficiently strong signal to allow for robust rate inference. Interestingly, whilst Chaguza-pt-1 also had the longest sampling window in this study, five other datasets with 8–15 sequences exceeded the 149-day window of Khatamzas-pt-1 but nevertheless failed to produce an adequate signal according to the date randomization test.

As commonly done (Chaguza *et al*. 2023, Stanevich *et al*. 2023, Marques *et al*. 2024, Sigal *et al*. 2024), we derived evolutionary rates based on consensus sequences and hence ignored within-host evolutionary dynamics. Assessment of full within-host viral diversity would require a representative sample of independently evolving quasispecies and accounting for distinctive variant frequencies within these subpopulations (Smith *et al*. 2024). Based on our literature and database searches, the availability of raw sequence data in public repositories is even more restricted than what is seen for consensus sequences. Furthermore, we utilized consensus sequences as provided by the original publications, implying that distinct methodologies as well as different variant calling thresholds have been used for consensus sequence reconstruction across datasets (see Supplementary Table S6 for details). However, since we compare molecular dating methods on a within-dataset level, possible biases introduced by differences between consensus sequence reconstruction methods can be considered negligible.

To evaluate the pace of intrahost viral evolution with respect to the previously presented within-host rates (Choi *et al*. 2020, Borges *et al*. 2021, Ciuffreda *et al*. 2021, Karim *et al*. 2021, Brandolini *et al*. 2023, Chaguza *et al*. 2023, Stanevich *et al*. 2023, Marques *et al*. 2024, Sigal *et al*. 2024), we inferred rate estimates based on a full genome analysis. The second rationale for focusing on genome-wide analysis stems directly from the data properties: since our aim was to infer evolutionary rates separately for each dataset, and given that most lacked sufficient signal for reliable rate estimation even at the genome-wide level, we considered robust gene-level analyses not feasible. However, previous studies have reported evolutionary rate variation between different genomic regions of SARS-CoV-2 (Pereson *et al*. 2021, Wang *et al*. 2022), leaving the characterization of gene-specific rate differentiations between within-host and host-to-host viral evolution an open question for future research.

Generally, our findings propose that viral phylogenies reconstructed based on consensus sequences alone cannot fully elucidate within-host dynamics of SARS-CoV-2. A comprehensive understanding of the underlying drivers shaping the intrahost evolution would require the development of models accounting jointly for multiple evolutionary processes, including host immune responses (Terbot *et al*. 2023). Therefore, robust evaluation of intrahost viral evolution necessitates systematically collected samples over the course of infection, larger cohorts, and improved clinical metadata documentation for statistically robust conclusions.

### Notable variation in rate estimates caused by method-specific limitations

Our results revealed notable variations in evolutionary rates, both between the datasets and across different molecular dating methods. Whereas the between-dataset differences suggest discrepancies in viral population dynamics amongst hosts and exemplifies the complexities of intrahost viral evolution, the latter highlights the overall shortcomings of SARS-CoV-2 phylogenetic inference, as thoroughly detailed in Morel *et al*. (2021). In short, estimates generated within this study by using RTT tend to be consistently higher than rates obtained with LSD2 and TreeDater, which yielded rather similar results within each dataset. In contrast, rate estimates obtained using BEAST2 showed lesser degrees of consistency in comparison to LSD2 and TreeDater results. The variations in rate estimates observed between different dating methods within a dataset can theoretically be attributed to several bias-causing factors in the data or the phylogenetic analyses: (i) issues with sequence quality, such as sequencing artefacts and ambiguous sites, (ii) undetected recombination events, (iii) discrepancies in tree topologies, and (iv) inherent limitations of the specific molecular dating methods employed.

Firstly, to mitigate the potential impacts of data quality issues, we filtered out known problematic sequence positions during the initial analysis and treated ambiguous characters as informative in each molecular dating framework. Secondly, since even minor levels of recombination can affect phylogenetic analysis, including evolutionary rate estimation (Schierup and Hein 2000), we excluded the possibility of interclade recombination during data collection by applying strict inclusion criteria, i.e. evidence confirming a long-term infection and verification of all sequences within a dataset representing the same Pango lineage. Detection of intraclade recombination, however, is highly challenging due to similarity of consensus sequences amongst coexisting quasispecies, resulting in an insufficient number of polymorphic sites for reliable recombination analysis. Consequently, signatures of intraclade recombination may be indistinguishable from recurrent mutations at the molecular level. Therefore, the varying sensitivities of molecular dating methods to unidentified intraclade recombination become trivial, as these events—whether they are misspecified recombinations or true recurrent mutations—are accounted for through the implementation of a substitution model.

Thirdly, as our datasets consist of genetically highly similar sequences, a weak phylogenetic signal may complicate the root estimation and result in topological differences between the output trees produced with different molecular dating approaches. However, the possibility of topological dissimilarities and errors being the cause for the rate variation between methods was excluded through three additional analyses. Within the first approach, we re-assessed evolutionary rates with LSD2 by utilizing a SARS-CoV-2 reference sequence as an outgroup but did not detect any prominent discrepancies in the inferred rate estimates. In the second approach, we employed RTT and LSD2 to re-estimated rates for Chaguza-pt-1 by incorporating a ‘founder sequence’ as suggested by Sigal *et al*. (2024). Once again, LSD2 demonstrated excessive robustness across various rooting strategies, whilst RTT produced highly inconsistent rate estimates, always exceeding those derived with LSD2. In the third approach, we contrasted time-tree topologies generated with BEAST2 and LSD2, and although we observed topological disparities for some of the datasets, we couldn’t detect any systematic correlations between topological differences and inflated BEAST2 rate estimates explaining the variation (Table 2).

**Table 2:** Summary of the results for nine datasets for which evolutionary rates were determined with RTT, LSD2, TreeDater, and BEAST2.

Dataset (Number of sequences)	Temporal signal strict clock (DRT)	Temporal signal relaxed clock (DRT)	Deviation from a clock-like evolution^a^	Degree of temporal clustering^b^	Rate estimates BEAST2 vs. LSD2 & TreeDater^c^	Tree topology BEAST2 vs. LSD2^d^	Tree topology BEAST2 strict vs. relaxed^e^	Signals of positive selection^f^
Brandolini-pt-1 (*N* = 8)	Weak	Weak	Modest	Unresolved	BEAST2 $\approx$ others	Modest variation	Modest variation	Yes (S gene)
Caccuri-pt-1 (*N* = 12)	Strong	Weak	Modest	Low	BEAST2 $\approx$ others	Notable variation	Modest variation	No
Chaguza-pt-1 (*N* = 30)	Strong	Strong	Notable	High	BEAST2 > others	Modest variation	Modest variation	Yes (S gene)
Choi-pt-1 (*N* = 9)	Weak	Weak	Modest	Low	BEAST2 > others	Modest variation	Identical	Yes (S gene)
Halfmann-pt-1 (*N* = 12)	Strong	Weak	Notable	High	BEAST2 > others	Modest variation	Identical	Yes (S gene)
Harari-pt-5 (*N* = 9)	Weak	Weak	Notable	High	BEAST2 > others	Modest variation	Modest variation	No
Huygens-pt-2 (*N* = 13)	Weak	Weak	Modest	Unresolved	BEAST2 $\approx$ others	Notable variation	Identical	No
Khatamzas-pt-1 (*N* = 21)	Strong	Strong	Modest	High	BEAST2 > others	Modest variation	Identical	No
Lee-pt-4 (*N* = 8)	Weak	Weak	Notable	Unresolved	BEAST2 $\approx$ others	Modest variation	Identical	Yes (ORF1ab)

a Estimated based on the coefficient of rate variation (Supplementary Fig. S8).

b Estimated based on TC statistics (detailed values for three parallel runs are presented in Supplementary Table S15).

c Comparison of point estimates (BEAST2 median estimates vs. LSD2 & TreeDater mean estimates) (see Fig. 3 and Supplementary Table S10).

d Estimated based on tree similarity and distance measures as proposed in Smith (2020) (detailed values presented in Supplementary Table S14, see also Supplementary Figs. S11–S19).

e Estimated based on tree similarity and distance measures as proposed in Smith (2020) (detailed values presented in Supplementary Table S14, see also Supplementary Figs. S11–S19).

f Results from Z-test of positive selection available in Supplementary Table S16.

Lastly, as we could not explain the differences between dating approaches by sequencing artefacts, ambiguous characters, recombination, or inconsistencies within the tree structure, we consider that differences may arise due to intrinsic characteristics of different methodologies. Several findings presented within this study confirm the problematic usage of root-to-tip regression as an explicit approach for molecular dating (Rambaut *et al*. 2016; Duchene *et al*. 2020a) and further highlight RTTs’ incompatibility for the intrahost framework. Due to the inherently high degree of non-independence of intrahost sequences, the simplified assumption of genetic changes accumulating independently along the lineages leads to a pseudoreplication, where particularly the mutations occurring at the deeper branches of the tree are contributing to multiple root-to-tip distances. As the RTT regression method does not explicitly model the shared ancestry, varying degrees of phylogenetic dependence between a within-host sample set and a collection of sequences randomly drawn from a large background population may explain the notably higher intrahost evolutionary rates reported by Chaguza *et al*. (2023), Stanevich *et al*. (2023), Harari *et al*. (2024), and Marques *et al*. (2024), which were based solely on RTT analysis. Furthermore, our finding that RTT estimates are markedly higher than those from LSD2 or TreeDater, particularly for sample series with limited temporal signal (Supplementary Table S11), has two key implications. First, it underscores the critical importance of evaluating data properties prior to molecular clock analyses. Second, it raises concerns about previously reported accelerated rate estimates obtained with RTT from datasets characterized by small sample sizes, narrow sampling windows, or both (Stanevich *et al*. 2023, Harari *et al*. 2024, Marques *et al*. 2024), as such datasets likely fail to meet the requirements for robust rate estimation (Duchene *et al*. 2020b). Notably, even though our datasets contain, on average, three times more samples and three times longer sampling windows than, for example, chronic-like clades analysed in Harari *et al*. (2024), reliable rate inference was only possible for two of our sample series. This strongly suggests that previously reported estimates may be shaped by methodological biases or insufficient data signal, rather than reflecting true biological processes.

Conversely, the high Bayesian rate estimates observed are likely attributable to strong phylo-temporal clustering, indicated by ladder-like topologies typical of intrahost genealogies (Grenfell *et al*. 2004). BEAST2 has been demonstrated to be profoundly sensitive to such clustering (Duchêne *et al*. 2016, Tong *et al*. 2018), since it reduces the number of independent calibration points, thereby decreasing information content and increasing uncertainty, leading to an upward bias in rate estimates (Ho *et al*. 2011b; Duchêne *et al*. 2015b; Tong *et al*. 2018). In contrast, LSD2 and TreeDater are less vulnerable to temporal clustering effects (To *et al*. 2016, Volz and Frost 2017, Tong *et al*. 2018). Despite the majority of the sample series displaying a ladder-like structure, reliable quantification of phylo-temporal clustering was achievable only for the largest datasets, Chaguza-pt-1 and Khatamzas-pt-1, for which strong phylo-temporal clustering likely explains the high BEAST2 rate estimates. Consequently, rate estimates derived with LSD2 and TreeDater are presumably closer to the true rates than those obtained using RTT or BEAST2.

### Comparison with rate estimates obtained from acute infections provides no evidence for elevated intrahost rates

Previous studies have brought the intrahost molecular rate estimates to a broader context through comparison with either RTT estimates obtained from a randomly sampled background population (Chaguza *et al*. 2023, Stanevich *et al*. 2023) or with a point estimate obtained from the literature (Borges *et al*. 2021, Ciuffreda *et al*. 2021, Brandolini *et al*. 2023, Sigal *et al*. 2024). The latter typically contrasts within-host mutation accumulation with a global rate estimate from the pandemic’s early stages (8.0e-04 subst./site/year). Later studies, however, have reported highly variable rates of SARS-CoV-2 evolution on a population scale, making inferences derived from a single point estimate somewhat ambiguous. Furthermore, the assumption of genetic changes accumulating along a single viral lineage overlooks the coexistence of genetically distinct viral populations (Kemp *et al*. 2021, Pérez-Lago *et al*. 2021, Harari *et al*. 2022, Brandolini *et al*. 2023, Chaguza *et al*. 2023), as also seen in some datasets included in this study (Supplementary Fig. S2).

Principally, a direct comparison of within-host and between-host rates may not be straightforward since molecular rate variation is not solely dependent on the mutation rate. Instead, the demographic history of the population has been found to alter the strength of genetic drift and selection, subsequently introducing rate variation through time (Ohta and Kimura 1971, Ohta 1987, Ohta 2002) (for review see (Bromham and Penny 2003)). Rate variability and accuracy have in addition shown to be impacted due to ‘time-dependency’ (Ho *et al*. 2005; Ho *et al*. 2011a), the degree of phylogenetic tree imbalance (Duchêne *et al*. 2015a), the presence of a pronounced population structure (Navascués and Emerson 2009), and the temporal distribution of sampling dates (Ho *et al*. 2007). To address these biases from demographic processes, we compared rates derived in this study to a variety of previously published estimates that have been retrieved by using diverse molecular dating methodologies and obtained from different datasets representing different timescales and phases of the pandemic (Supplementary Table S12). These published point estimates vary between ~3e-04 and 16e-04 subst./site/year (Supplementary Table S12), with the lowest estimates reported for viral lineages circulating during later stages of the pandemic (Hill *et al*. 2022, Neher 2022). Since the datasets analysed in our study primarily represent lineages from the earlier pandemic phase, which have been associated with higher evolutionary rates (Neher 2022), the lowest published host-to-host estimates are likely not appropriate as direct reference points for our comparisons (see Supplementary Table S13 for further details). Therefore, despite substantial discrepancies between datasets and methods used, intrahost evolutionary rates obtained in this study are generally consistent with rates reported from transmission chains of acutely infected individuals.

### Fluctuations in the viral population size shaping the rate of molecular evolution?

Elevated intrahost rates have been interpreted to reflect differences in viral population sizes, as the within-host viral population, unlike in host-to-host transmissions, is not constrained by transmission bottlenecks, potentially leading to faster evolutionary rates (Chaguza *et al*. 2023). Since our results do not indicate notable differences between host-to-host and within-host rates, we further explored whether changes in viral population size could result in intrahost rates comparable to those observed in acute infections. For the seven datasets with available data, frequent fluctuations in Ct or viral load estimates are apparent (Supplementary Fig. S32). Despite both measures being sensitive to inconsistencies in sampling method (for a review, see Puhach *et al*. 2022), successive intrahost genetic bottlenecks might have caused a significant loss in genetic diversity, as shown also for example for *Staphylococcus aureus* (Golubchik *et al*. 2013). Intriguingly, the genetic diversity of respiratory tract samples—comprising the majority of samples used in this study—has been found to be significantly lower compared to other anatomic sites presumably leading to a more pronounced genetic drift (Wang *et al*. 2021). Whereas the size of the intrahost genetic bottleneck is undoubtedly less stringent than what has been observed for host-to-host SARS-CoV-2 transmissions (Popa *et al*. 2020, Lythgoe *et al*. 2021), repeated bottlenecks combined with a small effective population size and thus a greater impact of random sampling might temporarily affect the frequency of novel mutations emerging, subsequently leading to lower molecular rates.

Additional evidence for population size changes shaping intrahost viral evolution comes from the observation that high degrees of phylo-temporal clustering can occur under neutral evolution due to repeated genetic bottlenecks (Gray *et al*. 2011). Whilst excessive phylo-temporal clustering is typically attributed to strong selective pressure (Grenfell *et al*. 2004), our study’s lack of strong selective signals suggests that selection alone may not account for ladder-like topologies. It is essential to note, however, that we tested selection signals by averaging over entire genes or genomes, and whilst we found no strong evidence of positive selection at this scale for eight out of the nine datasets investigated, specific mutations like E484K and del144 did emerge and subsequently became fixed within datasets, indicating positive selection of individual antibody escape mutations. Nevertheless, we anticipate that intrahost population size variations can explain, at least to some extent, molecular rates analogous to host-to-host rates. Similar conclusions have been made for HIV (Pybus and Rambaut 2009).

### Complex patterns of nonclocklike evolution

As our findings indicated non-clocklike evolution in all nine datasets, we explored the possible factors causing episodic evolution through ‘Patient case histories’. Temporal correspondences of mutational patterns, viral loads, and antibody-based treatments have previously suggested a correlation between viral rebound and the emergence of antibody evasion mutations (Harari *et al*. 2022). Building on this framework, our approach intends to explore mutational patterns on a more generic scale. By incorporating rate variation across branches, we aim to understand evolutionary changes between sampling points, offering insights even for unsampled parts of the phylogenetic tree. It is essential to note, however, that neither the approach used in this study nor the one exploited in Harari *et al*. (2022) can reveal the exact timing of novel mutations. Denser sampling over the course of infection would be required to distinguish if antibody evasion mutations arose at the time of viral rebound or during the preceding stages characterized by decreasing or undetectable levels of viral load. Whereas proper statistical testing was not feasible due to reasons like complex cycling patterns of viral load and wide variation in clinical conditions, visual examination of ‘Patient case histories’ did not reveal a temporal link between viral rebound and elevated levels of viral evolution. Instead, our findings emphasize the complexities of the interplay between intrahost viral bottlenecks, molecular rate variation, and therapies targeting the virus, which are influenced by factors beyond the scope of this study.

### Standardized framework for intrahost viral molecular rate inference needed

Whilst previous molecular dating studies of intrahost SARS-CoV-2 have typically relied on a single dating method (Chaguza *et al*. 2023, Stanevich *et al*. 2023, Marques *et al*. 2024, Sigal *et al*. 2024), our findings underscore the value of employing multiple approaches to estimate intrahost evolutionary rates accurately. We therefore recommend the following steps for robust tip-calibrated dating inference in within-host datasets: (i) determination of genetic diversity, (ii) evaluation of temporal signal, (iii) exploration of the tree topology, and (iv) comparison of different molecular dating methods. We further note the importance of the fundamental work evaluating through simulations the performance of distinct frameworks for molecular dating and temporal signal assessment (Duchêne *et al*. 2015a; Duchêne *et al*. 2016; Murray *et al*. 2016; To *et al*. 2016; Volz and Frost 2017; Tong *et al*. 2018; Duchene *et al*. 2020b). Whilst different methods generally perform well when applied to informative datasets, these studies have shown discrepancies to arise in the presence of strong phylo-temporal clustering, low substitution rates, notable amongst-lineage rate variation, and non-uniformly distributed sampling times. Since within-host datasets could be plausibly subject to all the aforementioned phenomena, additional simulation studies are necessary to more comprehensively address the intrinsic limitations of each methodology and their potential impact on viral intrahost evolutionary rate inference.

## Conclusions

Our findings have two types of implications: firstly, they emphasize the complexity of determining the within-host evolutionary rates, not restricted to intrahost evolution of SARS-CoV-2 but generalized also for other pathogens. By neglecting the limitations of the data or the method used, it is possible to obtain highly biased rate estimates and to hence draw invalid conclusions. Our findings highlight the significance of the systematic study of several intrahost datasets using different approaches in order to support reliable estimations. Secondly, in terms of SARS-CoV-2, our phylogenetic meta-analyses provide no evidence of generally elevated levels of viral evolution in immunocompromised individuals with chronic SARS-CoV-2 infection. Instead, within-host molecular rates inferred are comparable with rate estimates derived from host-to-host transmission chains. The contradicting results between this study and those of Chaguza *et al*. (2023), Stanevich *et al*. (2023), and Sigal *et al*. (2024) can likely be attributed to the method-specific limitations of different molecular dating approaches. Whilst our findings challenge previous claims of increased intrahost evolutionary rates, they do not refute the generally recognized theory of immunocompromised individuals serving as a source for emergence of new viral variants—a prolonged SARS-CoV-2 infection within an immunodeficient individual might promote the appearance of novel antibody escape mutations. Furthermore, our findings do not preclude the possibility of increased evolutionary rates amongst immunocompromised individuals. Instead, interpretations based solely on consensus sequences should be approached with caution, as they may obscure the full extent of within-host viral diversity.

## Supplementary Material

SI_VEVOLU-2025-041_R1_veaf065

SupplTables_S1-S8_Oeversti_Gaul_Jensen_Kuehnert_2025-02-10_veaf065

## Data Availability

No new data was created as part of this study. Instead, the findings in this study are based on previously published datasets. For the majority of the datasets included, accession information for the viral genomic sequences is given in Supplementary Table S3. Additionally, viral genomic data generated for Sonnleitner *et al*. (2022) is available in the Genome Sequence Archive as .bam files under the bioproject name PRJCA008906 (https://ngdc.cncb.ac.cn/bioproject/browse/PRJCA008906). Corresponding consensus sequences can be obtained through correspondence with the authors of Sonnleitner *et al*. (2022). Viral genomic data generated for Li *et al*. (2021) can be obtained through correspondence with the authors of Li *et al*. (2021). Viral genomic data generated for Jensen *et al*. (2021) can be obtained through correspondence with the authors of Jensen *et al*. (2021). Files associated with phylogenetic analysis are available in GitHub: https://github.com/tidelab/Persistent_SARS-CoV-2_evolutionary_rates.

## References

[ref1] Aksamentov I, Roemer C, Hodcroft EB et al. Nextclade: clade assignment, mutation calling and quality control for viral genomes. J Open Source Softw 2021;6:3773. 10.21105/joss.03773

[ref2] Attwood SW, Hill SC, Aanensen DM et al. Phylogenetic and phylodynamic approaches to understanding and combating the early SARS-CoV-2 pandemic. Nat Rev Genet 2022;23:547–62. 10.1038/s41576-022-00483-8 35459859 PMC9028907

[ref3] Baang JH, Smith C, Mirabelli C et al. Prolonged severe acute respiratory syndrome coronavirus 2 replication in an immunocompromised patient. J Infect Dis 2021;223:23–7. 10.1093/infdis/jiaa666 33089317 PMC7797758

[ref4] Borges V, Isidro J, Cunha M et al. Long-term evolution of SARS-CoV-2 in an immunocompromised patient with non-Hodgkin. Lymphoma 2021;6:e00244–21. 10.1128/mSphere.00244-21 PMC8386466 34319130

[ref5] Bouckaert R, Vaughan TG, Barido-Sottani J et al. BEAST 2.5: an advanced software platform for Bayesian evolutionary analysis. PLoS Comput Biol 2019;15:e1006650. 10.1371/journal.pcbi.1006650 30958812 PMC6472827

[ref6] Brandolini M, Zannoli S, Gatti G et al. Viral population heterogeneity and fluctuating mutational pattern during a persistent SARS-CoV-2 infection in an immunocompromised patient. Viruses 2023;15:291. 10.3390/v15020291 36851504 PMC9962589

[ref7] Bromham L, Penny D. The modern molecular clock. Nat Rev Genet 2003;4:216–24. 10.1038/nrg1020 12610526

[ref8] Caccuri F, Messali S, Bortolotti D et al. Competition for dominance within replicating quasispecies during prolonged SARS-CoV-2 infection in an immunocompromised host. Virus Evol 2022;8:veac042. 10.1093/ve/veac042 35706980 PMC9129230

[ref9] Chaguza C, Hahn AM, Petrone ME et al. Accelerated SARS-CoV-2 intrahost evolution leading to distinct genotypes during chronic infection. Cell Rep Med 2023;4:100943. 10.1016/j.xcrm.2023.100943 36791724 PMC9906997

[ref10] Choi B, Choudhary MC, Regan J et al. Persistence and evolution of SARS-CoV-2 in an immunocompromised host. N Engl J Med 2020;383:2291–3. 10.1056/NEJMc2031364 33176080 PMC7673303

[ref11] Ciuffreda L, Lorenzo-Salazar JM, Alcoba-Florez J et al. Longitudinal study of a SARS-CoV-2 infection in an immunocompromised patient with X-linked agammaglobulinemia. J Inf Secur 2021;83:607–35. 10.1016/j.jinf.2021.07.028 PMC8316714 34329678

[ref12] Corcione S, De Nicolò A, Montrucchio G et al. Real-life study on the pharmacokinetic of remdesivir in ICU patients admitted for severe COVID-19 pneumonia. Br J Clin Pharmacol 2021;87:4861–7. 10.1111/bcp.14895 33990984 PMC8239594

[ref13] De Maio N, Walker C, Borger R et al. Masking strategies for SARS-CoV-2 alignments - SARS-CoV-2 coronavirus / software and tools. Virological 2020. Available from: https://virological.org/t/masking-strategies-for-sars-cov-2-alignments/480

[ref14] Didelot X, Croucher NJ, Bentley SD et al. Bayesian inference of ancestral dates on bacterial phylogenetic trees. Nucleic Acids Res 2018;46:e134–e134. 10.1093/nar/gky783 30184106 PMC6294524

[ref15] Didelot X, Siveroni I, Volz EM. Additive uncorrelated relaxed clock models for the dating of genomic epidemiology Phylogenies.Crandall K, editor. Mol Biol Evol 2021;38:307–17. 10.1093/molbev/msaa193 32722797 PMC8480190

[ref16] Drummond AJ, Pybus OG, Rambaut A et al. Measurably evolving populations. Trends Ecol Evol 2003;18:481–8. 10.1016/S0169-5347(03)00216-7

[ref17] Drummond AJ, Ho SYW, Phillips MJ et al. Relaxed phylogenetics and dating with confidence. PLoS Biol 2006;4:699–710. 10.1371/journal.pbio.0040088 PMC1395354 16683862

[ref18] Duchêne D, Duchêne S, Ho SYW. Tree imbalance causes a bias in phylogenetic estimation of evolutionary timescales using heterochronous sequences. Mol Ecol Resour 2015a;15:785–94. 10.1111/1755-0998.12352 25431227

[ref19] Duchêne S, Duchêne D, Holmes EC et al. The performance of the date-randomization test in phylogenetic analyses of time-structured virus data. Mol Biol Evol 2015b;32:1895–906. 10.1093/molbev/msv056 25771196

[ref20] Duchêne S, Geoghegan JL, Holmes EC et al. Estimating evolutionary rates using time-structured data: a general comparison of phylogenetic methods. Bioinformatics 2016;32:3375–9. 10.1093/bioinformatics/btw421 27412094

[ref21] Duchene S, Featherstone L, Haritopoulou-Sinanidou M et al. Temporal signal and the phylodynamic threshold of SARS-CoV-2. Virus Evol 2020a;6:veaa061. 10.1093/ve/veaa061 33235813 PMC7454936

[ref22] Duchene S, Lemey P, Stadler T et al. Bayesian evaluation of temporal signal in measurably evolving populations. Mol Biol Evol 2020b;37:3363–79. 10.1093/molbev/msaa163 32895707 PMC7454806

[ref23] Ferretti L, Golubchik T, Di Lauro F et al. Biased estimates of phylogenetic branch lengths resulting from the discretised gamma model of site rate heterogeneity. bioRxiv. 2024. Available from: https://www.biorxiv.org/content/10.1101/2024.08.01.606208v2 10.1093/sysbio/syag037 42161371

[ref24] Firth C, Kitchen A, Shapiro B et al. Using time-structured data to estimate evolutionary rates of double-stranded DNA viruses. Mol Biol Evol 2010;27:2038–51. 10.1093/molbev/msq088 20363828 PMC3107591

[ref25] Gandhi S, Klein J, Robertson AJ et al. De novo emergence of a remdesivir resistance mutation during treatment of persistent SARS-CoV-2 infection in an immunocompromised patient: a case report. Nat Commun 2022;13:1547. 10.1038/s41467-022-29104-y 35301314 PMC8930970

[ref26] Ghafari M, Du Plessis L, Pybus OG et al. Time dependence of SARS-CoV-2 substitution rates. Virological 2020. Available from: https://virological.org/t/time-dependence-of-sars-cov-2-substitution-rates/542

[ref27] Gojobori T, Moriyama EN, Kimura M. Molecular clock of viral evolution, and the neutral theory. Proc Natl Acad Sci 1990;87:10015–8. 10.1073/pnas.87.24.10015 2263602 PMC55305

[ref28] Golubchik T, Batty EM, Miller RR et al. Within-host evolution of Staphylococcus aureus during asymptomatic carriage. PLoS One 2013;8:e61319. 10.1371/journal.pone.0061319 23658690 PMC3641031

[ref29] Gray RR, Pybus OG, Salemi M. Measuring the temporal structure in serially sampled phylogenies: temporal structure in phylogenies. Methods Ecol Evol 2011;2:437–45. 10.1111/j.2041-210X.2011.00102.x 22121470 PMC3222587

[ref30] Grenfell BT, Pybus OG, Gog JR et al. Unifying the epidemiological and evolutionary dynamics of pathogens. Science 2004;303:327–32. 10.1126/science.1090727 14726583

[ref31] Gupta A, Gonzalez-Rojas Y, Juarez E et al. Early treatment for Covid-19 with SARS-CoV-2 neutralizing antibody Sotrovimab. N Engl J Med 2021;385:1941–50. 10.1056/NEJMoa2107934 34706189

[ref32] Halfmann PJ, Minor NR, Haddock LA III et al. Evolution of a globally unique SARS-CoV-2 spike E484T monoclonal antibody escape mutation in a persistently infected, immunocompromised individual. Virus Evol 2023;9:veac104. 10.1093/ve/veac104 37692895 PMC10491860

[ref33] Hall T . BioEdit: a user-friendly biological sequence alignment editor and analysis program for windows 95/98/NT. Nucleic Acids Symp Ser 1999;41:95–8.

[ref34] Harari S, Tahor M, Rutsinsky N et al. Drivers of adaptive evolution during chronic SARS-CoV-2 infections. Nat Med 2022;28:1501–8. 10.1038/s41591-022-01882-4 35725921 PMC9307477

[ref35] Harari S, Miller D, Fleishon S et al. Using big sequencing data to identify chronic SARS-Coronavirus-2 infections. Nat Commun 2024;15:648. 10.1038/s41467-024-44803-4 38245511 PMC10799923

[ref36] Hettle D, Hutchings S, Muir P et al. Persistent SARS-CoV-2 infection in immunocompromised patients facilitates rapid viral evolution: retrospective cohort study and literature review. Clin Infect Pract 2022;16:100210. 10.1016/j.clinpr.2022.100210 36405361 PMC9666269

[ref37] Hill V, Du Plessis L, Peacock TP et al. The origins and molecular evolution of SARS-CoV-2 lineage B.1.1.7 in the UK. Virus Evol 2022;8:veac080. 10.1093/ve/veac080 36533153 PMC9752794

[ref38] Ho SYW, Larson G. Molecular clocks: when timesare a-changin. Trends Genet 2006;22:79–83. 10.1016/j.tig.2005.11.006 16356585

[ref39] Ho SYW, Phillips MJ, Cooper A et al. Time dependency of molecular rate estimates and systematic overestimation of recent divergence times. Mol Biol Evol 2005;22:1561–8. 10.1093/molbev/msi145 15814826

[ref40] Ho SYW, Kolokotronis SO, Allaby RG. Elevated substitution rates estimated from ancient DNA sequences. Biol Lett 2007;3:702–5. 10.1098/rsbl.2007.0377 17785261 PMC2391221

[ref41] Ho SYW, Lanfear R, Bromham L et al. Time-dependent rates of molecular evolution. Mol Ecol 2011a;20:3087–101. 10.1111/j.1365-294X.2011.05178.x 21740474

[ref42] Ho SYW, Lanfear R, Phillips MJ et al. Bayesian estimation of substitution rates from ancient DNA sequences with low information content. Syst Biol 2011b;60:366–75. 10.1093/sysbio/syq099 21296909

[ref43] Höhna S, Landis MJ, Heath TA et al. RevBayes: Bayesian phylogenetic inference using graphical models and an interactive model-specification language. Syst Biol 2016;65:726–36. 10.1093/sysbio/syw021 27235697 PMC4911942

[ref44] Huelsenbeck JP, Ronquist F, Nielsen R et al. Bayesian inference of phylogeny and its impact on evolutionary biology. Science 2001;294:2310–4. 10.1126/science.1065889 11743192

[ref45] Humeniuk R, Mathias A, Kirby BJ et al. Pharmacokinetic, pharmacodynamic, and drug-interaction profile of remdesivir, a SARS-CoV-2 replication inhibitor. Clin Pharmacokinet 2021;60:569–83. 10.1007/s40262-021-00984-5 33782830 PMC8007387

[ref46] Huygens S, Gharbharan A, Serroukh Y et al. High-titer convalescent plasma plus nirmatrelvir/ritonavir treatment for non-resolving COVID-19 in six immunocompromised patients. J Antimicrob Chemother 2023;78:1644–8. 10.1093/jac/dkad144 37248664 PMC10320105

[ref47] Jensen B, Luebke N, Feldt T et al. Emergence of the E484K mutation in SARS-COV-2-infected immunocompromised patients treated with bamlanivimab in Germany. Lancet Reg Health Eur 2021;8:100164. 10.1016/j.lanepe.2021.100164 34278371 PMC8278033

[ref48] Kalyaanamoorthy S, Minh BQ, Wong TKF et al. ModelFinder: fast model selection for accurate phylogenetic estimates. Nat Methods 2017;14:587–9. 10.1038/nmeth.4285 28481363 PMC5453245

[ref49] Kang H, Wang Y, Tong Z et al. Retest positive for SARS-CoV-2 RNA of “recovered” patients with COVID-19: persistence, sampling issues, or re-infection? J Med Virol 2020;92:2263–5. 10.1002/jmv.26114 32492212 PMC7300489

[ref50] Karim F, Moosa M, Gosnell B et al. Persistent SARS-CoV-2 infection and intra-host evolution in association with advanced HIV infection. medRxiv. 2021. Available from: https://www.medrxiv.org/content/10.1101/2021.06.03.21258228v1

[ref51] Katoh K, Standley DM. MAFFT multiple sequence alignment software version 7: improvements in performance and usability. Mol Biol Evol 2013;30:772–80. 10.1093/molbev/mst010 23329690 PMC3603318

[ref52] Kemp SA, Collier DA, Datir RP et al. SARS-CoV-2 evolution during treatment of chronic infection. Nature 2021;592:277–82. 10.1038/s41586-021-03291-y 33545711 PMC7610568

[ref53] Khatamzas E, Antwerpen MH, Rehn A et al. Accumulation of mutations in antibody and CD8 T cell epitopes in a B cell depleted lymphoma patient with chronic SARS-CoV-2 infection. Nat Commun 2022;13:5586. 10.1038/s41467-022-32772-5 36151076 PMC9508331

[ref54] Lee CY, Shah MK, Hoyos D et al. Prolonged SARS-CoV-2 infection in patients with lymphoid malignancies. Cancer Discov 2022;12:62–73. 10.1158/2159-8290.CD-21-1033 34753749 PMC8758535

[ref55] Li W-H, Masako T, Sharp P. Rates and dates of divergence between AIDS virus nucleotide sequences. Mol Biol Evol 1988;5:313–30. 10.1093/oxfordjournals.molbev.a040503 3405075

[ref56] Li L, Li S, Pan Y et al. An immunocompetent patient with high neutralizing antibody Titers who shed COVID-19 virus for 169 days — China, 2020. China CDC Wkly 2021;3:688–91. 10.46234/ccdcw2021.163 34594968 PMC8393007

[ref57] Lynch M, Macori G, Fanning S et al. Genomic evolution of SARS-CoV-2 virus in immunocompromised patient. Ireland Emerg Infect Dis 2021;27:2499–501. 10.3201/eid2709.211159 34161223 PMC8386806

[ref58] Lythgoe KA, Hall M, Ferretti L et al. SARS-CoV-2 within-host diversity and transmission. Science 2021;372:eabg0821. 10.1126/science.abg0821 33688063 PMC8128293

[ref59] Lythgoe KA, Golubchik T, Hall M et al. Lineage replacement and evolution captured by 3 years of the United Kingdom Coronavirus (COVID-19) Infection Survey. Proc R Soc B Biol Sci 2023;290:20231284. 10.1098/rspb.2023.1284 PMC10581763 37848057

[ref60] Marques AD, Graham-Wooten J, Fitzgerald AS et al. SARS-CoV-2 evolution during prolonged infection in immunocompromised patients. MBio 2024;15:e00110–24. 10.1128/mbio.00110-24 38364100 PMC10936176

[ref61] Minh BQ, Schmidt HA, Chernomor O et al. IQ-TREE 2: new models and efficient methods for phylogenetic inference in the genomic era. Mol Biol Evol 2020;37:1530–4. 10.1093/molbev/msaa015 32011700 PMC7182206

[ref62] Morel B, Barbera P, Czech L et al. Phylogenetic analysis of SARS-CoV-2 data is difficult. Mol Biol Evol 2021;38:1777–91. 10.1093/molbev/msaa314 33316067 PMC7798910

[ref63] Murray GGR, Wang F, Harrison EM et al. The effect of genetic structure on molecular dating and tests for temporal signal. Methods Ecol Evol 2016;7:80–9. 10.1111/2041-210X.12466 27110344 PMC4832290

[ref64] Nascimento FF, Reis M dos, Yang Z. 2017. A biologist’s guide to Bayesian phylogenetic analysis. Nat Ecol Evol 1:1446–54, 10.1038/s41559-017-0280-x. 28983516 10.1038/s41559-017-0280-x PMC5624502

[ref65] Navascués M, Emerson BC. Elevated substitution rate estimates from ancient DNA: model violation and bias of Bayesian methods. Mol Ecol 2009;18:4390–7. 10.1111/j.1365-294X.2009.04333.x 19735451

[ref66] Neher RA . Contributions of adaptation and purifying selection to SARS-CoV-2 evolution. Virus Evol 2022;8:veac113. 10.1093/ve/veac113 37593203 PMC10431346

[ref67] Norström MM, Prosperi MCF, Gray RR et al. PhyloTempo: a set of R scripts for assessing and visualizing temporal clustering in genealogies inferred from serially sampled viral sequences. Evol Bioinforma 2012;8:261–9. 10.4137/EBO.S9738 PMC3382462 22745529

[ref68] Ohta T . Very slightly deleterious mutations and the molecular clock. J Mol Evol 1987;26:1–6. 10.1007/BF02111276 3125329

[ref69] Ohta T . Near-neutrality in evolution of genes and gene regulation. Proc Natl Acad Sci 2002;99:16134–7. 10.1073/pnas.252626899 12461171 PMC138577

[ref70] Ohta T, Kimura M. On the constancy of the evolutionary rate of cistrons. J Mol Evol 1971;1:18–25. 10.1007/BF01659391 4377445

[ref71] Pereson MJ, Flichman DM, Martínez AP et al. Evolutionary analysis of SARS-CoV-2 spike protein for its different clades. J Med Virol 2021;93:3000–6. 10.1002/jmv.26834 33512021 PMC8013443

[ref72] Pérez-Lago L, Aldámiz-Echevarría T, García-Martínez R et al. Different within-host viral evolution dynamics in severely immunosuppressed cases with persistent SARS-CoV-2. Biomedicines 2021;9:808. 10.3390/biomedicines9070808 34356872 PMC8301427

[ref73] Popa A, Genger JW, Nicholson MD et al. Genomic epidemiology of superspreading events in Austria reveals mutational dynamics and transmission properties of SARS-CoV-2. Sci Transl Med 2020;12:1–14. 10.1126/scitranslmed.abe2555 PMC7857414 33229462

[ref74] Puhach O, Meyer B, Eckerle I. SARS-CoV-2 viral load and shedding kinetics. Nat Rev Microbiol 2022;21:147–61. 10.1038/s41579-022-00822-w 36460930 PMC9716513

[ref75] Pybus OG, Rambaut A. Evolutionary analysis of the dynamics of viral infectious disease. Nat Rev Genet 2009;10:540–50. 10.1038/nrg2583 19564871 PMC7097015

[ref76] Rambaut A . Estimating the rate of molecular evolution: incorporating non-contemporaneous sequences into maximum likelihood phylogenies. Bioinformatics 2000;16:395–9. 10.1093/bioinformatics/16.4.395 10869038

[ref77] Rambaut A, Lam TT, Carvalho LM et al. Exploring the temporal structure of heterochronous sequences using TempEst (formerly path-O-gen). Virus Evol 2016;2:1–7. 10.1093/ve/vew007 PMC4989882 27774300

[ref78] Rambaut A, Holmes EC, O’Toole Á et al. A dynamic nomenclature proposal for SARS-CoV-2 lineages to assist genomic epidemiology. Nat Microbiol 2020;5:1403–7. 10.1038/s41564-020-0770-5 32669681 PMC7610519

[ref79] Ramsden C, Melo FL, Figueiredo LuizM, Holmes EC, Zanotto PMA, the VGDN Consortium . High rates of molecular evolution in hantaviruses. Mol Biol Evol 2008;25:1488–92. 10.1093/molbev/msn093 18417484

[ref80] Ramsden C, Holmes EC, Charleston MA. Hantavirus evolution in relation to its rodent and insectivore hosts: No evidence for codivergence. Mol Biol Evol 2009;26:143–53. 10.1093/molbev/msn234 18922760

[ref81] Riddell AC, Kele B, Harris K et al. Generation of novel severe acute respiratory syndrome coronavirus 2 variants on the B.1.1.7 lineage in 3 patients with advanced human immunodeficiency Virus-1 disease. Clin Infect Dis 2022;75:2016–8. 10.1093/cid/ciac409 35616095 PMC9213850

[ref82] Rieux A, Balloux F. Inferences from tip-calibrated phylogenies: a review and a practical guide. Mol Ecol 2016;25:1911–24. 10.1111/mec.13586 26880113 PMC4949988

[ref83] Rieux A, Khatchikian CE. Tipdatingbeast: an r package to assist the implementation of phylogenetic tip-dating tests using beast. Mol Ecol Resour 2017;17:608–13. 10.1111/1755-0998.12603 27717245

[ref84] Rockett R, Basile K, Maddocks S et al. Resistance mutations in SARS-CoV-2 Delta variant after Sotrovimab use. N Engl J Med 2022;386:1477–9. 10.1056/NEJMc2120219 35263515 PMC8929376

[ref85] Schierup MH, Hein J. Consequences of recombination on traditional phylogenetic analysis. Genetics 2000;156:879–91. 10.1093/genetics/156.2.879 11014833 PMC1461297

[ref86] Sigal A, Neher RA, Lessells RJ. The consequences of SARS-CoV-2 within-host persistence. Nat Rev Microbiol 2024;23:288–302. 10.1038/s41579-024-01125-y 39587352

[ref87] Smith MR . Information theoretic generalized Robinson–Foulds metrics for comparing phylogenetic trees. Bioinformatics 2020;36:5007–13. 10.1093/bioinformatics/btaa614 32619004

[ref88] Smith E, Hamilton WL, Warne B, et al. Variable rates of SARS-CoV-2 evolution in chronic infections. PLoS Pathogens 2025;21:e1013109. 10.1371/journal.ppat.1013109 PMC12061394 40294077

[ref89] Sonnleitner ST, Prelog M, Sonnleitner S et al. Cumulative SARS-CoV-2 mutations and corresponding changes in immunity in an immunocompromised patient indicate viral evolution within the host. Nat Commun 2022;13:2560. 10.1038/s41467-022-30163-4 35538074 PMC9090742

[ref90] Stanevich OV, Alekseeva EI, Sergeeva M et al. SARS-CoV-2 escape from cytotoxic T cells during long-term COVID-19. Nat Commun 2023;14:149. 10.1038/s41467-022-34033-x 36627290 PMC9831376

[ref91] Tamura K, Stecher G, Kumar S. MEGA11: molecular evolutionary genetics analysis version 11. Mol Biol Evol 2021;38:3022–7. 10.1093/molbev/msab120 33892491 PMC8233496

[ref92] Tay JH, Baele G, Duchene S. Detecting episodic evolution through Bayesian inference of molecular clock models. Mol Biol Evol 2023;40:msad212. 10.1093/molbev/msad212 PMC10560005 37738550

[ref93] Terbot JW, Johri P, Liphardt SW et al. Developing an appropriate evolutionary baseline model for the study of SARS-CoV-2 patient samples. PLoS Pathog 2023;19:e1011265. 10.1371/journal.ppat.1011265 37018331 PMC10075409

[ref94] To TH, Jung M, Lycett S et al. Fast dating using least-squares criteria and algorithms. Syst Biol 2016;65:82–97. 10.1093/sysbio/syv068 26424727 PMC4678253

[ref95] Tong KJ, Duchêne DA, Duchêne S et al. A comparison of methods for estimating substitution rates from ancient DNA sequence data. BMC Evol Biol 2018;18:70. 10.1186/s12862-018-1192-3 PMC5956955 29769015

[ref96] Turakhia Y, De Maio N, Thornlow B et al. Stability of SARS-CoV-2 phylogenies.Barsh GS, editor. PLoS Genet 2020;16:e1009175. 10.1371/journal.pgen.1009175 33206635 PMC7721162

[ref97] Volz EM, Frost SDW. Scalable relaxed clock phylogenetic dating. Virus Evol 2017;3:1–9.

[ref98] Wang Y, Wang D, Zhang L et al. Intra-host variation and evolutionary dynamics of SARS-CoV-2 populations in COVID-19 patients. Genome Med 2021;13:1–13. 10.1186/s13073-021-00847-5 33618765 PMC7898256

[ref99] Wang S, Xu X, Wei C et al. Molecular evolutionary characteristics of SARS-CoV-2 emerging in the United States. J Med Virol 2022;94:310–7. 10.1002/jmv.27331 34506640 PMC8662038

[ref100] Weigang S, Fuchs J, Zimmer G et al. Within-host evolution of SARS-CoV-2 in an immunosuppressed COVID-19 patient as a source of immune escape variants. Nat Commun 2021;12:6405. 10.1038/s41467-021-26602-3 34737266 PMC8568958

[ref101] Zapor M . Persistent detection and infectious potential of SARS-CoV-2 virus in clinical specimens from COVID-19 patients. Viruses 2020;12:1384. 10.3390/v12121384 33287245 PMC7761721

[ref102] Zuckerkandl E, Pauling LB. Molecular disease, evolution, and genetic heterogeneity. In: Kasha M, Pullman B (eds) Horizons in biochemistry. In: Kasha M, Pullman B (eds.), *Horizons in Biochemistry*, pp. 189–225. New York: Academic Press, 1962.

