# Supplementary material for: Phylogenetic meta-analysis of persistent SARS-CoV-2 infections in immunocompromised individuals highlights the challenges of robust evolutionary rate estimation caused by low genetic diversity
Source: Virus Evol. 2025 Aug 30;11(1):veaf065. doi: 10.1093/ve/veaf065 (PMC12477587; doi:10.1093/ve/veaf065)
Supplement: SI_VEVOLU-2025-041_R1_veaf065 [file si_vevolu-2025-041_r1_veaf065.pdf]

Supplementary tables and figures

Phylogenetic meta-analysis of persistent SARS-CoV-2 infections in immunocompromised individuals highlights the challenges of robust evolutionary rate estimation caused by low genetic diversity

Sanni Översti, Emily Gaul, Björn-Erik Ole Jensen, Denise Kühnert

Supplementary tables S1–S8 can be found from a separate Excel file

Supplementary table S1. Sequence metadata.
Supplementary table S2. Patient metadata.
Supplementary table S3. Sequence accession information.
Supplementary table S4. All reported Ct values / viral loads of patient viral specimens.
Supplementary table S5. List of supporting publications.
Supplementary table S6. Information on bioinformatics procedures used in each supporting publication.
Supplementary table S7. Patient list.
Supplementary table S8. The absolute numbers of missing characters (N) and ambiguous characters (R, Y, K, M, S, W, B, D, H, and V) for each sequence included in the study.

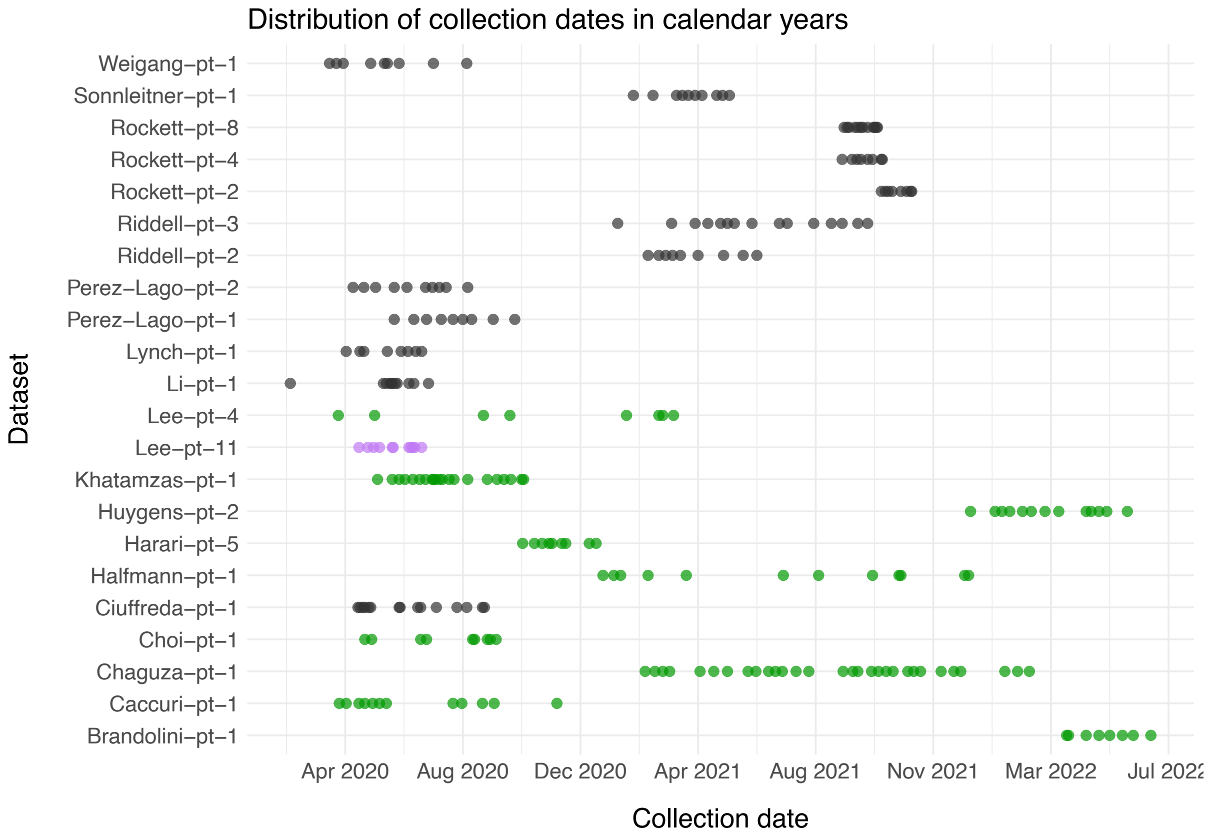

Supplementary figure S1. Temporal distribution of sample collection points. Collection dates are represented in calendar years. Datasets are coded according to their temporal signal: Purple circles indicate datasets with no temporal signal, grey circles indicate poor ('Questionable') temporal signal whereas green circles denote datasets with sufficient temporal signal (evaluated based on analysis with RTT, LSD2 and TreeDater). As for the following datasets the collection dates were not given as calendar units, they are omitted: Baang-pt-1, Gandhi-pt-1, Jensen-pt-2 and Kemp-pt-1. Collection dates are given relative to the first sample of each dataset (Day0) in Figure 2.

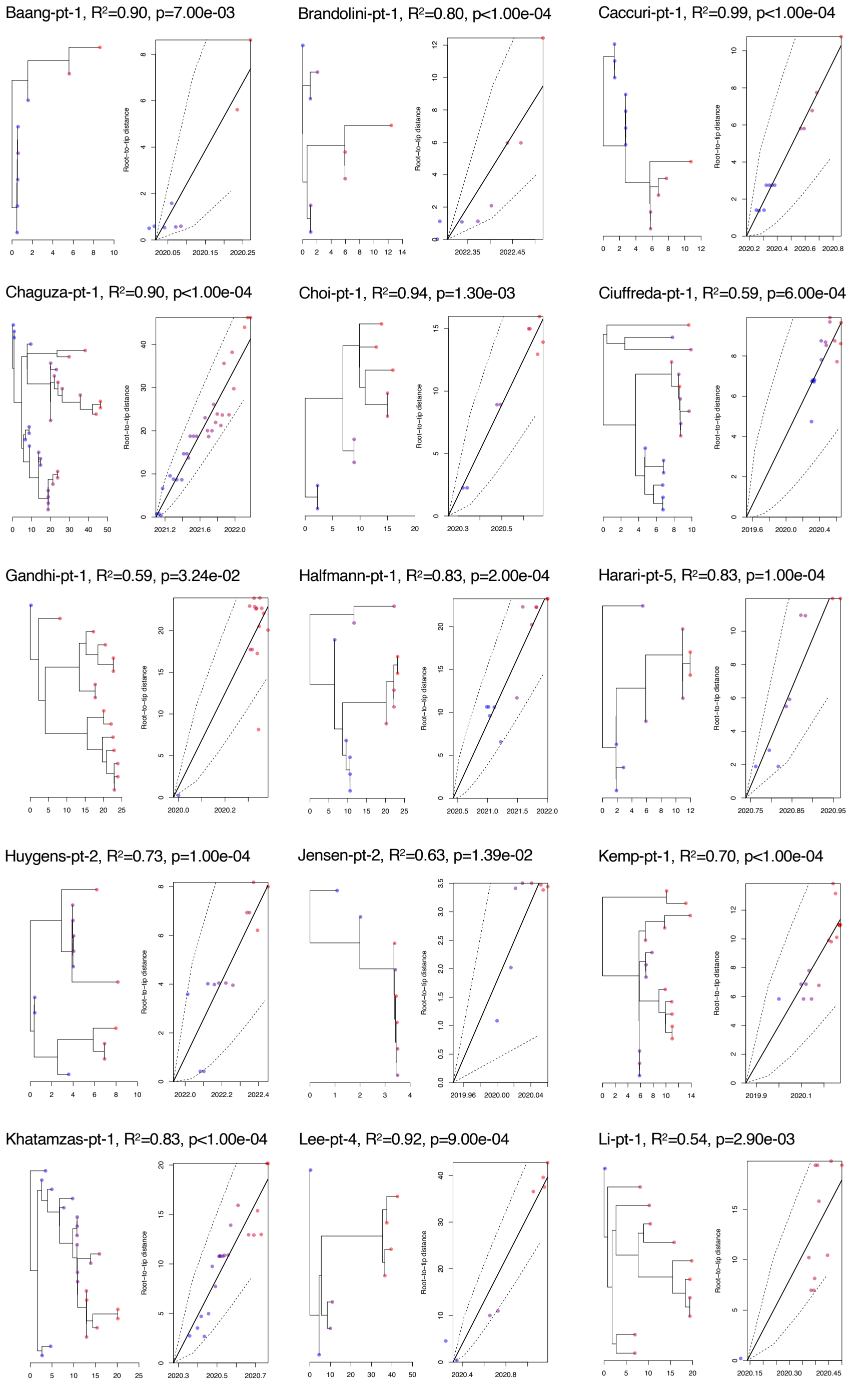

Supplementary figure S2. Root-to-tip regression plots for 25 datasets included in this study (Lee-pt-11 omitted due to lack of temporal signal). R package BactDating (Didelot et al. 2018) was used to perform regression of root-to-tip analysis and to generate the figures. Note, that as BactDating requires sampling dates in calendar units, for datasets lacking collection dates in calendar years (i.e. Baang-pt-1, Gandhi-pt-1, Jensen-pt-2 and Kemp-pt-1) the collection day for Day0 sequence was arbitrarily set to 2020-01-01 and collection dates for the rest of the sequences were calculated accordingly (i.e. for Baang-pt-1: Day5 sample → 2020-01-06, Day15 sample → 2020-01-16, etc.). Therefore, for these four datasets the timescales on the x axis do not indicate the actual sampling window.

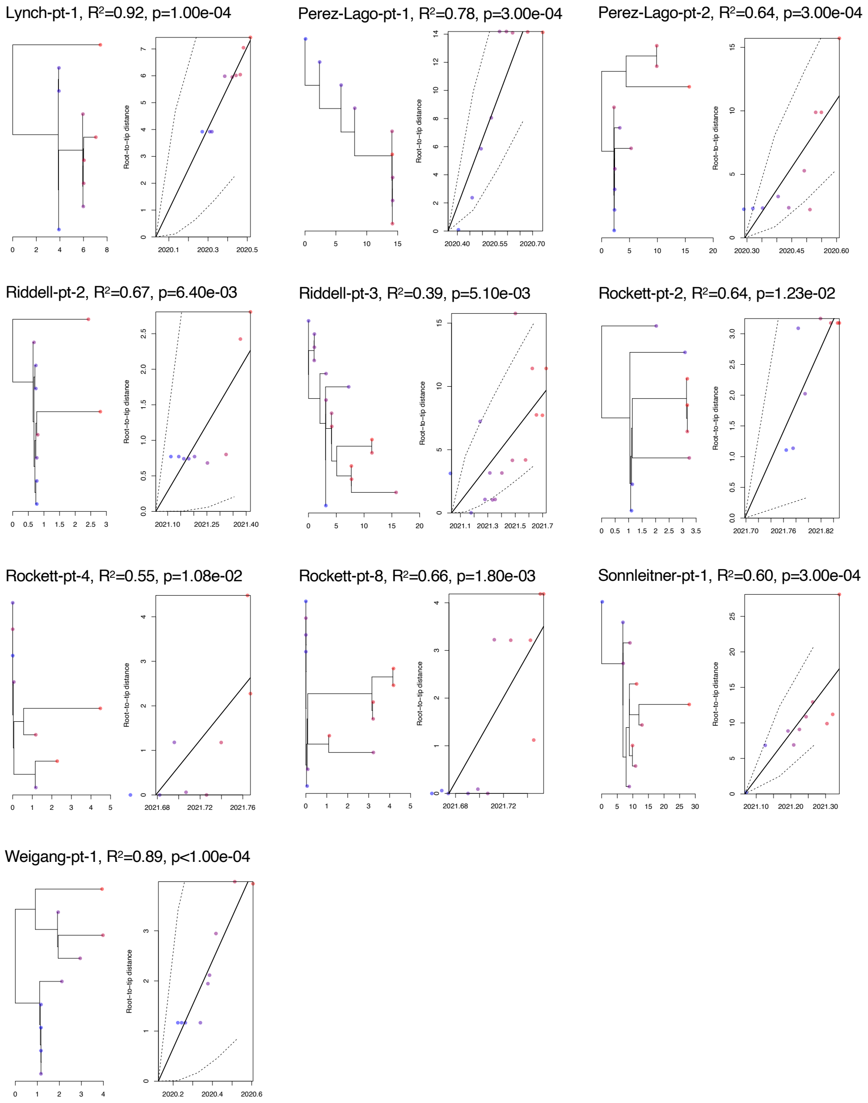

Supplementary figure S2 (cont.). Root-to-tip regression plots for 25 datasets included in this study (Lee-pt-11 omitted due to lack of temporal signal). R package BactDating (Didelot et al. 2018) was used to perform regression of root-to-tip analysis and to generate the figures. Note, that as BactDating requires sampling dates in calendar units, for datasets lacking collection dates in calendar years (i.e. Baang-pt-1, Gandhi-pt-1, Jensen-pt-2 and Kemp-pt-1) the collection day for Day0 sequence was arbitrarily set to 2020-01-01 and collection dates for the rest of the sequences were calculated accordingly (i.e. for Baang-pt-1: Day5 sample → 2020-01-06, Day15 sample → 2020-01-16, etc.). Therefore, for these four datasets the timescales on the x axis do not indicate the actual sampling window.

Supplementary table S9. Mean number of pairwise differences between sequence pairs within each dataset. See also Supplementary figure S3.

Dataset	Number of sequences	Sampling window (days)	Temporal signal	Pairwise differences mean	Pairwise differences stdev
Baang-pt-1	8	99	Questionable	3.54	1.27
Brandolini-pt-1	8	86	Yes	5.07	1.50
Caccuri-pt-1	12	222	Yes	4.53	1.39
Chaguza-pt-1	30	392	Yes	19.43	2.09
Choi-pt-1	9	134	Yes	10.56	1.74
Ciuffreda-pt-1	15	129	Questionable	8.18	1.60
Gandhi-pt-1	15	141	Questionable	19.11	2.96
Halfmann-pt-1	12	373	Yes	14.03	2.18
Harari-pt-5	9	75	Yes	5.94	1.46
Huygens-pt-2	13	160	Yes	5.62	1.55
Jensen-pt-2	8	22	Questionable	1.04	0.56
Kemp-pt-1	16	100	Questionable	7.98	1.31
Khatamzas-pt-1	21	149	Yes	6.05	1.06
Lee-pt-4	8	342	Yes	20.64	2.85
Lee-pt-11	11	64	No	0.36	0.24
Li-pt-1	10	140	Questionable	14.40	2.33
Lynch-pt-1	8	77	Questionable	1.32	0.78
Perez-Lago-pt-1	9	123	Questionable	3.83	1.17
Perez-Lago-pt-2	10	117	Questionable	4.76	1.00
Riddell-pt-2	9	111	Questionable	1.03	0.47
Riddell-pt-3	15	255	Questionable	4.12	0.82
Rockett-pt-2	8	31	Questionable	2.57	0.91
Rockett-pt-4	8	40	Questionable	1.64	0.85
Rockett-pt-8	12	34	Questionable	2.39	0.80
Sonnleitner-pt-1	10	98	Questionable	5.69	1.29
Weigang-pt-1	9	140	Questionable	3.17	0.96

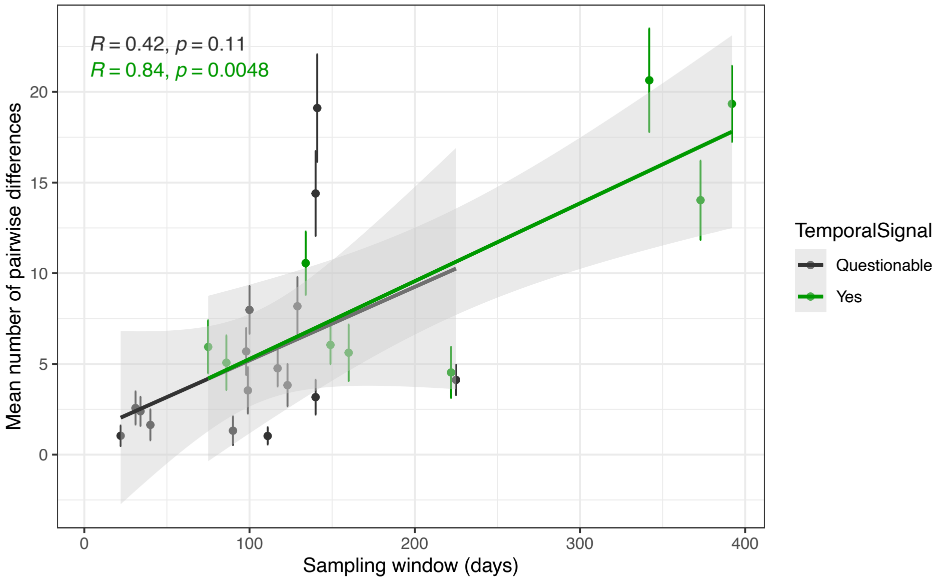

Supplementary figure S3. Mean number of pairwise differences between sequence pairs within each dataset plotted against the sampling window. Circles represent mean estimates and vertical lines standard deviations for each dataset. Green colour denotes datasets for which temporal signal was considered sufficient based on LSD2 and TreeDater analysis whereas grey colour denotes datasets for which temporal signal was not adequately assigned. Based on a linear regression model, statistically significant indications of strong correlations between sampling window and mean number of pairwise distances was found only for a group of datasets exhibiting adequate temporal signal.

Supplementary table S10. Evolutionary rate estimates reconstructed with RTT, LSD2, TreeDater and BEAST2. Evolutionary rates are given in substitutions/site/year. For LSD2 and TreeDater mean estimates are given with lower and upper bounds of confidence intervals. For estimates inferred with BEAST2 median estimates with 95% highest posterior density intervals (HPDI) are presented. For Rockett-pt-4 the rate estimate was successfully determined only with RTT, whereas for Riddell-pt-3, Sonnleitner-pt-1, and Weigang-pt-1 the estimates were successfully obtained with RTT and TreeDater but not with LSD2.

**Dataset (Number of sequences)**	**RTT**	**LSD2 Collapse none**	**LSD2 Collapse default**	**TreeDater Strict clock**	**TreeDater Relaxed clock**	**BEAST2 Strict clock**	**BEAST2 Relaxed clock**
Baang-pt-1 (N=8)	9.73e-04	1.00e-10 [1.00e-10, 3.35e-04]	9.21e-06 [1.00e-10, 3.29e-04]	5.90e-04 [1.62e-04, 2.13e-03]	5.39e-04 [1.23e-04, 2.35e-03]	NA	NA
Brandolini-pt-1 (N=8)	1.48e-03	5.25e-04 [4.70e-05, 1.03e-03]	1.00e-03 [3.35e-04, 1.58e-03]	9.32e-04 [5.39e-04, 1.61e-03]	8.61e-04 [1.25e-04, 5.90e-03]	7.96e-04 [2.27e-04, 1.43e-03]	8.01e-04 [1.34e-04, 1.67e-03]
Caccuri-pt-1 (N=12)	5.08e-04	4.54e-04 [1.36e-04, 7.65e-04]	4.64e-04 [1.74e-04, 7.34e-04]	3.52e-04 [1.16e-04, 1.07e-03]	3.38e-04 [1.42e-04, 8.00e-04]	5.25e-04 [2.29e-04, 9.04e-04]	5.39e-04 [2.08e-04, 1.01e-03]
Chaguza-pt-1 (N=30)	1.27e-03	5.58e-04 [3.58e-04, 7.17e-04]	4.60e-04 [2.67e-04, 6.55e-04]	9.04e-04 [7.35e-04, 1.11e-03]	5.96e-04 [2.52e-04, 1.41e-03]	9.74e-04 [7.68e-04, 1.22e-03]	9.40e-04 [5.75e-04, 1.43e-03]
Choi-pt-1 (N=9)	1.22e-03	7.74e-04 [5.36e-05, 1.45e-03]	7.76e-04 [8.81e-05, 1.31e-03]	9.27e-04 [3.85e-04, 2.23e-03]	9.46e-04 [4.30e-04, 2.08e-03]	1.24e-03 [5.66e-04, 1.97e-03]	1.25e-03 [5.56e-04, 1.99e-03]
Ciuffreda-pt-1 (N=15)	2.86e-04	6.07e-05 [1.00e-10, 1.95e-04]	7.25e-05 [1.00e-10, 2.14e-04]	5.89e-05 [3.13e-05, 1.10e-04]	1.27e-04 [1.03e-05, 1.56e-03]	NA	NA
Gandhi-pt-1 (N=15)	1.88e-03	4.62e-04 [1.00e-10, 9.85e-04]	4.61e-04 [1.00e-10, 1.01e-03]	1.61e-03 [1.21e-03, 2.15e-03]	1.74e-03 [1.29e-03, 2.36e-03]	NA	NA
Halfmann-pt-1 (N=12)	5.04e-04	4.24e-04 [2.25e-04, 7.25e-04]	4.25e-04 [2.28e-04, 6.97e-04]	2.79e-04 [1.01e-04, 7.65e-04]	1.85e-04 [2.85e-06, 1.19e-02]	8.43e-04 [5.77e-04, 1.12e-03]	8.17e-04 [2.76e-04, 1.53e-03]
Harari-pt-5 (N=9)	1.97e-03	1.06e-03 [3.52e-04, 1.98e-03]	1.05e-03 [2.82e-04, 1.68e-03]	1.14e-03 [4.22e-04, 3.09e-03]	9.10e-04 [1.65e-04, 5.02e-03]	1.72e-03 [7.43e-04, 2.90e-03]	1.88e-03 [6.50e-04, 3.36e-03]
Huygens-pt-2 (N=13)	5.22e-04	3.69e-04 [1.64e-04, 5.98e-04]	3.65e-04 [1.37e-04, 6.36e-04]	2.37e-04 [7.83e-05, 7.21e-04]	2.41e-04 [6.78e-05, 8.61e-04]	4.02e-04 [1.66e-04, 6.90e-04]	4.37e-04 [1.50e-04, 7.47e-04]
Jensen-pt-2 (N=8)	1.15e-03	8.18e-05 [1.00e-10, 7.06e-04]	8.67e-05 [1.00e-10, 4.83e-04]	6.17e-05 [1.38e-05, 2.74e-04]	3.51e-05 [6.36e-07, 1.94e-03]	NA	NA
Kemp-pt-1 (N=16)	9.03e-04	3.00e-04 [1.00e-10, 5.24e-04]	3.84e-04 [1.18e-04, 6.12e-04]	4.84e-04 [1.53e-04, 1.52e-03]	4.82e-04 [1.89e-04, 1.22e-03]	NA	NA

Khatamzas-pt-1 (N=21)	1.26e-03	1.09e-03 [6.26e-04, 1.65e-03]	9.55e-04 [4.66e-04, 1.34e-03]	1.11e-03 [6.87e-04, 1.79e-03]	1.13e-03 [6.85e-04, 1.87e-03]	1.57e-03 [9.88e-04, 2.24e-03]	1.63e-03 [9.39e-04, 2.43e-03]
Lee-pt-4 (N=8)	1.52e-03	1.06e-03 [7.00e-04, 1.52e-03]	1.06e-03 [7.00e-04, 1.52e-03]	1.03e-03 [7.46e-04, 1.42e-03]	6.82e-04 [1.51e-05, 3.07e-02]	8.55e-04 [2.53e-04, 1.34e-03]	9.35e-04 [2.44e-04, 1.59e-03]
Li-pt-1 (N=10)	1.66e-03	5.25e-04 [1.00e-10, 9.16e-04]	5.24e-04 [1.94e-05, 8.78e-04]	1.17e-03 [7.61e-04, 1.80e-03]	1.22e-03 [8.92e-04, 1.68e-03]	NA	NA
Lynch-pt-1 (N=8)	5.08e-04	4.07e-04 [1.00e-10, 9.55e-04]	2.79e-04 [1.00e-10, 6.29e-04]	3.22e-04 [8.78e-05, 1.18e-03]	3.53e-04 [4.01e-05, 3.10e-03]	NA	NA
Pérez-Lago-pt-1 (N=9)	1.59e-03	7.82e-05 [1.00e-10, 3.51e-04]	7.48e-05 [1.00e-10, 3.03e-04]	7.06e-04 [3.29e-04, 1.51e-03]	9.84e-04 [1.53e-04, 6.29e-03]	NA	NA
Pérez-Lago-pt-2 (N=10)	1.18e-03	1.13e-04 [1.00e-10, 2.95e-04]	1.14e-04 [1.00e-10, 2.97e-04]	3.51e-04 [1.29e-04, 9.57e-04]	2.73e-04 [6.63e-05, 1.12e-03]	NA	NA
Riddell-pt-2 (N=9)	2.09e-04	1.16e-04 [1.00e-10, 3.18e-04]	1.18e-04 [1.00e-10, 3.29e-04]	1.01e-04 [3.66e-05, 2.83e-04]	8.72e-05 [5.25e-06, 1.44e-03]	NA	NA
Riddell-pt-3 (N=15)	4.67e-04	NA	NA	2.99e-04 [1.45e-04, 6.17e-04]	1.00e-03 [5.29e-05, 1.90e-02]	NA	NA
Rockett-pt-2 (N=8)	7.62e-04	1.03e-03 [6.54e-05, 2.32e-03]	9.03e-04 [1.00e-10, 2.42e-03]	9.47e-04 [1.61e-04, 5.54e-03]	9.44e-04 [1.15e-04, 7.69e-03]	NA	NA
Rockett-pt-4 (N=8)	9.93e-03	NA	NA	NA	NA	NA	NA
Rockett-pt-8 (N=12)	1.48e-03	4.57e-05 [1.00e-10, 3.74e-04]	2.60e-04 [1.00e-10, 6.37e-04]	4.72e-04 [7.47e-05, 2.98e-03]	4.06e-04 [9.32e-06, 1.77e-02]	NA	NA
Sonnleitner-pt-1 (N=10)	2.15e-03	NA	NA	1.74e-03 [1.06e-03, 2.83e-03]	2.11e-03 [7.18e-04, 6.22e-03]	NA	NA
Weigang-pt-1 (N=9)	2.91e-04	1.88e-04 [1.00e-10, 4.86e-04]	NA	2.07e-04 [5.81e-05, 7.37e-04]	1.91e-04 [2.10e-05, 1.74e-03]	NA	NA

Supplementary table S11. Average ratios of point estimates derived using RTT compared to other methods, calculated as RTT_{mean}/Alternative $method_{mean/median}$ across all relevant datasets. Datasets lacking results from one or both methods were excluded from comparisons.
* Excluded datasets: Lee-pt-11, Rocket-pt-4, Baang-pt-1, Riddell-pt-3, Sonnleitner-pt-1.
** Excluded datasets: Lee-pt-11, Rocket-pt-4, Riddell-pt-3, Sonnleitner-pt-1, Weigang-pt-1.
*** Excluded datasets: Lee-pt-11, Rocket-pt-4.

	All datasets	Datasets with temporal signal (All with N=9)	Datasets with 'Questionable' temporal signal
RTT / LSD2 collapse none	5.42 (N=21)*	1.65	8.25 (N=12)
RTT / LSD2 collapse default	8.91 (N=21)**	1.57	14.40 (N=12)
RTT / TreeDater strict clock	2.55 (N=24)***	1.56	3.14 (N=15)
RTT / TreeDater relaxed clock	3.15 (N=24)***	1.89	3.91 (N=15)
RTT / BEAST2 strict clock	–	1.19	–
RTT / BEAST2 relaxed clock	–	1.16	–

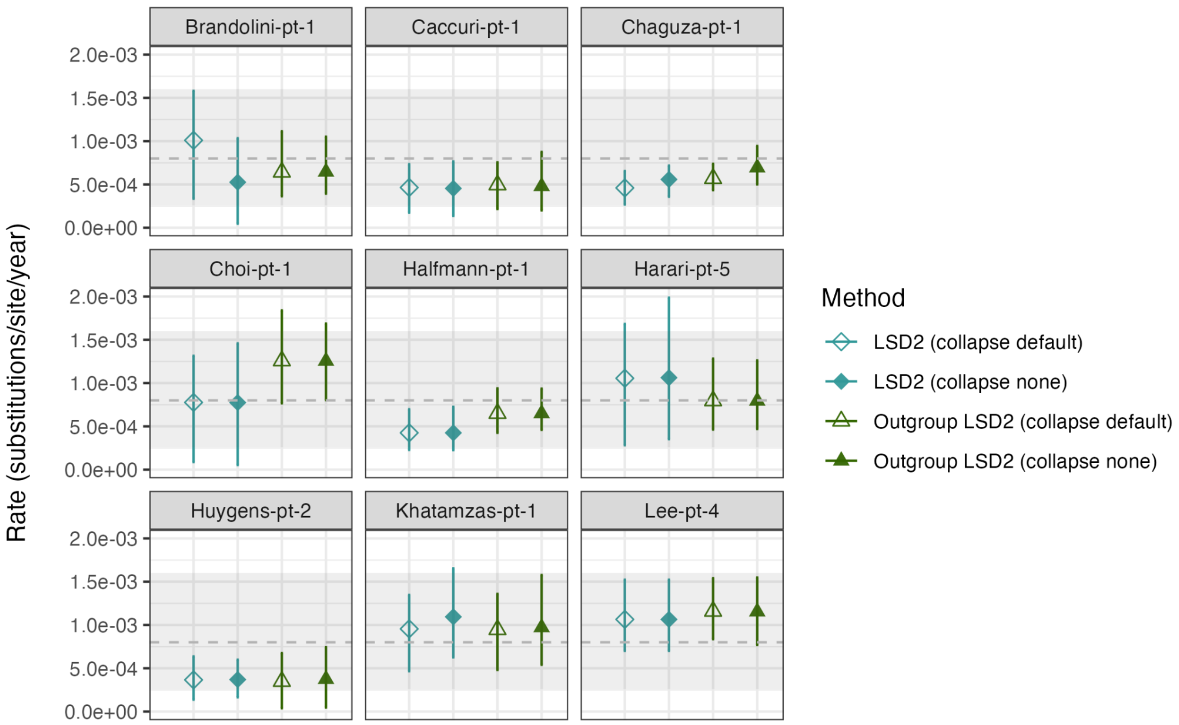

Supplementary figure S4. Testing the impact of inclusion of an outgroup for evolutionary rate estimates inferred with LSD2. As an outgroup reference sequence Wuhan-Hu-1 (NC_045512.2) was used. In each panel, the Y axis denotes the evolutionary rate in substitutions/site/year. Diamonds represent mean estimates obtained without an outgroup (i.e. the best-fit root position is estimated according to LSD criteria, see (To et al. 2016)), whereas triangles represent mean estimates obtained when a tree is being rooted with a known outgroup. Grey dashed line represents the commonly used SARS-CoV-2 substitution rate estimate of 8.0e-04 subst./site/year (Ghafari et al. 2020). The grey shaded area denotes the lowest and highest mean evolutionary rate estimates for SARS-CoV-2 collected from various publications (3e-04 – 16e-04 subst./site/year, see Supplementary table S11).

Supplementary text S1. Evaluation of the 'founder sequence' approach and potential incorrect rooting

A recent study by (Sigal et al. 2024) introduced a novel approach for estimating SARS-CoV-2 intrahost evolutionary rates by identifying a putative 'founder sequence' from publicly available databases. While the authors do not specify how to correctly identify the founder sequence, its selection is likely based on genetic similarity to the intrahost sample series and temporal alignment with the presumed infection onset. Evolutionary rates are inferred using pairwise distances to the 'founder sequence' as well as to the reference sequence (Wuhan-Hu-1), with different weights assigned to ambiguous and non-ambiguous mutations. Sigal et al. (2024) applied this method to six patients from prior studies (Chaguza et al. 2023, Khatamzas et al. 2023, Stanevich et al. 2023, and Karim et al. 2024), concluding that their estimates align

with or exceed evolutionary rates observed along branches leading to VOCs. However, several key assumptions and interpretations made by Sigal et al. (2024) require closer scrutiny.

First, the method appears to assume complete sequence independence, failing to account for shared genetic ancestry. Weighting ambiguous and non-ambiguous mutations differently does not mitigate this limitation. Second, the approach assumes rate homogeneity across lineages, a well-known limitation of, for instance, root-to-tip methods. Contrasting this assumption, the findings of our study provided evidence for non-clocklike evolution across all the sample series examined (Supplementary Figure S8). Furthermore, the approach oversimplifies molecular evolution by relying on absolute genetic distances rather than an appropriate substitution model. Lastly, the concept of a 'founder sequence' raises significant concerns. While presumably intended to mitigate rooting biases, it assumes that the identified sequence and intrahost sample series form a monophyletic clade, requiring not only genetic similarity but also epidemiological evidence – data that is rarely available. Moreover, in half of the cases analysed by Sigal et al. (2024), the first sequenced samples were obtained months after initial infection (e.g., 79, 122, and 209 days later), leaving the viral composition during these extended periods unknown. This makes the identification of a 'founder sequence' that accurately reflects the viral lineage entering these patients, impossible. In the absence of epidemiological and/or genetic evidence the selection of a 'founder sequence' becomes arbitrary and is likely to result in biased outcomes.

Given the major concerns outlined, the approach proposed by Sigal et al. (2024) requires further validation, and the reliability of their results should be critically reassessed. While a full validation is beyond the scope of this study, we conducted a small-scale sensitivity analysis to compare the robustness of RTT and LSD2 estimates using Chaguza-pt-1 as a representative case. The Chaguza-pt-1 patient was symptomatic but tested negative a few days before their first positive test (2020-11-21), whereas their household contact had already tested positive. Although details of the transmission chain are unavailable, Chaguza-pt-1 was likely infected through their household contact. Assuming an incubation period of ~5–7 days (Galmiche et al. 2023), both individuals were probably infected before mid-November. Based on this estimate, we identified through Nextstrain a putative 'founder sequence' that aligns temporally and shows close genetic similarity to Chaguza-pt-1 sample series (GenBank accession ID PP601523.1, with a collection date of 2020-11-09). However, as for Chaguza-pt-1 the first sequenced sample was obtained 79 days after the first positive test, the true genetic relatedness of the 'founder sequence' and the viral genotype initially infecting Chaguza-pt-1, remains unknown.

Supplementary Figure S5 compares the original rate estimates from this study with those obtained using either the 'founder sequence' or Wuhan-Hu-1 as an outgroup. RTT point estimates range from 8.6e-04 to 12.7e-04 subst./site/year, while mean estimates derived with LSD2 remain consistent (~ 5.0–6.0e-04 subst./site/year) regardless of the chosen rooting approach. Notably, all RTT estimates exceed those obtained with LSD2, yet the only point estimate significantly surpassing the reference value of 8.0e-4 subst./site/year is the original estimate of 12.7e-04 subst./site/year, which aligns with the rate derived in Chaguza et al.

(2024). This challenges their initial claims of accelerated intrahost evolution, as none of the alternative approaches explored here reproduce the elevated intrahost rate estimate. While the small sample size prevents definitive conclusions, the results highlight the robustness of LSD2 and illustrate three key points: 1) RTT is highly sensitive to different rooting strategies, 2) RTT consistently produces higher rate estimates than LSD2, and 3) since both methods used the same substitution tree as input, the observed discrepancies cannot be attributed solely to ambiguous sites, contrary to the hypothesis by Sigal et al. (2024). Instead, these findings strongly suggest that the differences stem from inherent limitations in the molecular dating methods themselves.

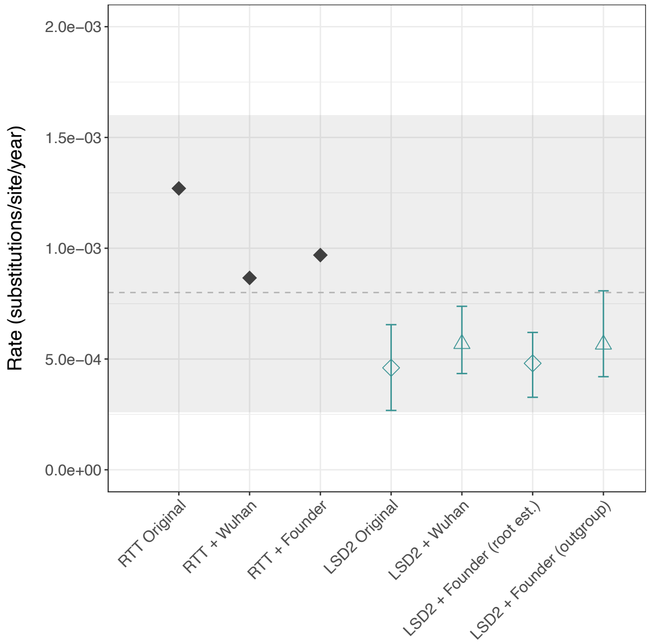

Supplementary figure S5. Testing the impact of inclusion of a 'founder sequence' for evolutionary rates estimates inferred with RTT and LSD2. For LSD2 the diamonds represent mean estimates without determining the outgroup (i.e. the best-fit root position is estimated according to LSD criteria, see (To et al. 2016)), whereas triangles represent mean estimates obtained when a tree is being rooted with a known outgroup (i.e. either Wuhan-Hu-1 or 'founder sequence'). Grey dashed line represents the commonly used SARS-CoV-2 substitution rate estimate of 8.0e-04 subst./site/year (Ghafari et al. 2020). The grey shaded area denotes the lowest and highest mean evolutionary rate estimates for SARS-CoV-2 collected from various publications (3e-04 – 16e-04 subst./site/year, see Supplementary table S11).

Supplementary table S12. Evolutionary rates obtained from literature and used as a reference. Table is an extension to the Table 1 presented (Attwood et al. 2022). Abbreviations as in (Attwood et al. 2022): BCP = Bayesian coalescent phylodynamic, MTBD = Multi-type birth-death, SC = Structured coalescent, BC + EG = Bayesian coalescent with exponential growth.

**Reference**	**Rate (subst./site/year)**	**95% HPD (if available)**	**Method**
(Douglas et al. 2021)	6.91e-04	6.00e-04, 7.78e-04	Phylodynamics: MTBD
(Seemann et al. 2020)	1.1e-03	Na	Phylodynamics: BCP + SC
(Douglas et al. 2021)	5.75e-04	4.96e-04, 6.47e-04	Phylodynamics: MTBD
(Alteri et al. 2021)	1.16e-03	1.01e-03, 1.32e-03	Phylodynamics: BCP, BCP + SC
(Douglas et al. 2021)	6.09e-04	5.16e-04, 7.03e-04	Phylodynamics: MTBD
(Komissarov et al. 2021)	9.43e-04	8.46e-04, 1.04e-03	Phylodynamics: BCP + SC
(Douglas et al. 2021)	8.00e-04	6.89e-04, 9.17e-04	Phylodynamics: MTBD
(Geidelberg et al. 2021)	1.30e-03	0.98e-03, 1.7e-03	Phylodynamics: BCP + CFEM
(Díez-Fuertes et al. 2021)	1.47e-03	1.08e-03, 1.87e-03	Phylogenetics: BC + EG
(Duchene et al. 2020)	1.1e-03	7.03e-04, 1.5e-03	Phylogenetics: BC + EG
(Bai et al. 2020)	1.60e-03	1.42e-03, 1.80e-03	Phylogenetics: BC + EG
(Pipes et al. 2021)	5.47e-04	3.05e-04, 7.89e-04	Bayesian Molecular clock rooting
(Duchene et al. 2020)	6.7e-04 – 8.8e-04	Na	Root-to-tip
(Chaguza et al. 2023)	5.83e-04	5.56e-04, 6.11e-04	Root-to-tip
(Fauver et al. 2020)	1.00e-03	Na	Root-to-tip
(Wolf et al. 2023)	9.82e-04	Na	Root-to-tip
(Neher 2022)	2.6e-04 – 9.3e-04	Na	Root-to-tip
(Hill et al. 2022)	3.9e-04 – 5.0e-04	Na	Root-to-tip

Supplementary table S13. Comparison of rate estimates from this study with those reported by Neher (2022) and Hill et al. (2022). The lowest host-to-host evolutionary rate estimates listed in Supplementary Table S12 originate from Neher (2022) and Hill et al. (2022), both of which focus primarily on viral lineages circulating during later stages of the pandemic. In contrast, most of the datasets analysed in this study correspond to earlier clades, such as 20A and 20B (Table 1), which have been shown to exhibit higher evolutionary rates than later-emerging clades (Neher 2022). Hill et al. (2022) specifically reported rate estimates for Alpha and Delta variants and their respective outgroups. Among our datasets, only three sample series fall into these categories: Alpha (Huygens-pt-2) and Delta (Riddell-pt-2 and Riddell-pt-3). As earlier lineages tend to evolve faster than later ones (Neher 2022), direct comparisons to Hill et al. (2022) are appropriate only for these three datasets. However, due to insufficient genetic diversity and weak temporal signal, the Delta datasets (Riddell-pt-2 and -pt-3) are not suitable for robust rate comparisons. In contrast, the Huygens-pt-2 dataset shows a clear temporal signal, and its mean rate estimates (2.4e-04 to 4.4e-04 subst./site/year) align well with those reported for Alpha by Neher (2022) and Hill et al. (2022). The table below summarises the overlap between the viral lineages analysed in this study and those included in Neher (2022) and Hill et al. (2022). For each clade, the number of sample series used in this study is reported.

Nextstrain clade	Number of sample series (in this study)	Neher 2022 Overall rate y^{-1}	Neher 2022 subs./site/year	Hill et al. 2022 subs./site/year
19B	1	19.46	6.5e-04	Na
19A	2	19.73	6.6e-04	Na
20A	7	15.08	5.0e-04	Na
20B	7	14.29	4.8e-04	Na
20D	1	Na	Na	Na
20G	1	Na	Na	Na
20I (Alpha)	2	11.56	3.9e-04	4.6e-04 (out-group 4.3e-04)
21J (Delta)	4	14.30	4.8e-04	Na
21K (Omicron)	1	13.48	4.5e-04	5.03 e-04 (out-group 3.88e-04)

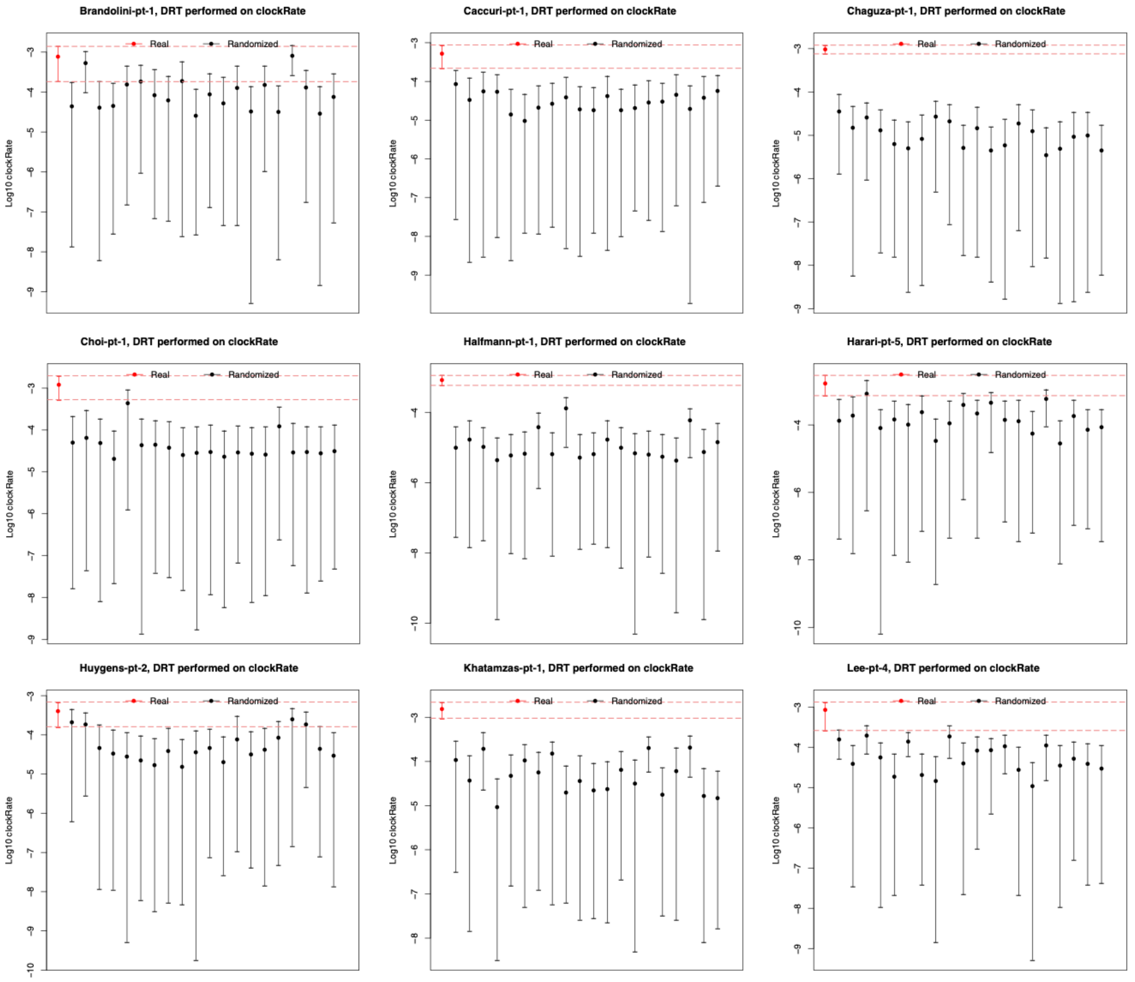

Supplementary figure S6. Date-randomisation test (DRT) performed on the clock rate parameter of the strict clock model. Each panel corresponds to estimates obtained from one dataset. Within each panel, an estimate indicated with red colour represents the real estimate whereas black colour denotes estimates obtained from date-randomized data sets. For each dataset date-randomization was performed twenty times. For clarity, on the Y axis evolutionary rate estimates are reported on a logarithmic scale. Overlapping 95% highest posterior density (HPD) distributions of real and randomized estimates might indicate that the strength of the temporal signal might not be sufficient enough to infer evolutionary rates with high confidence only based on tip-dating.

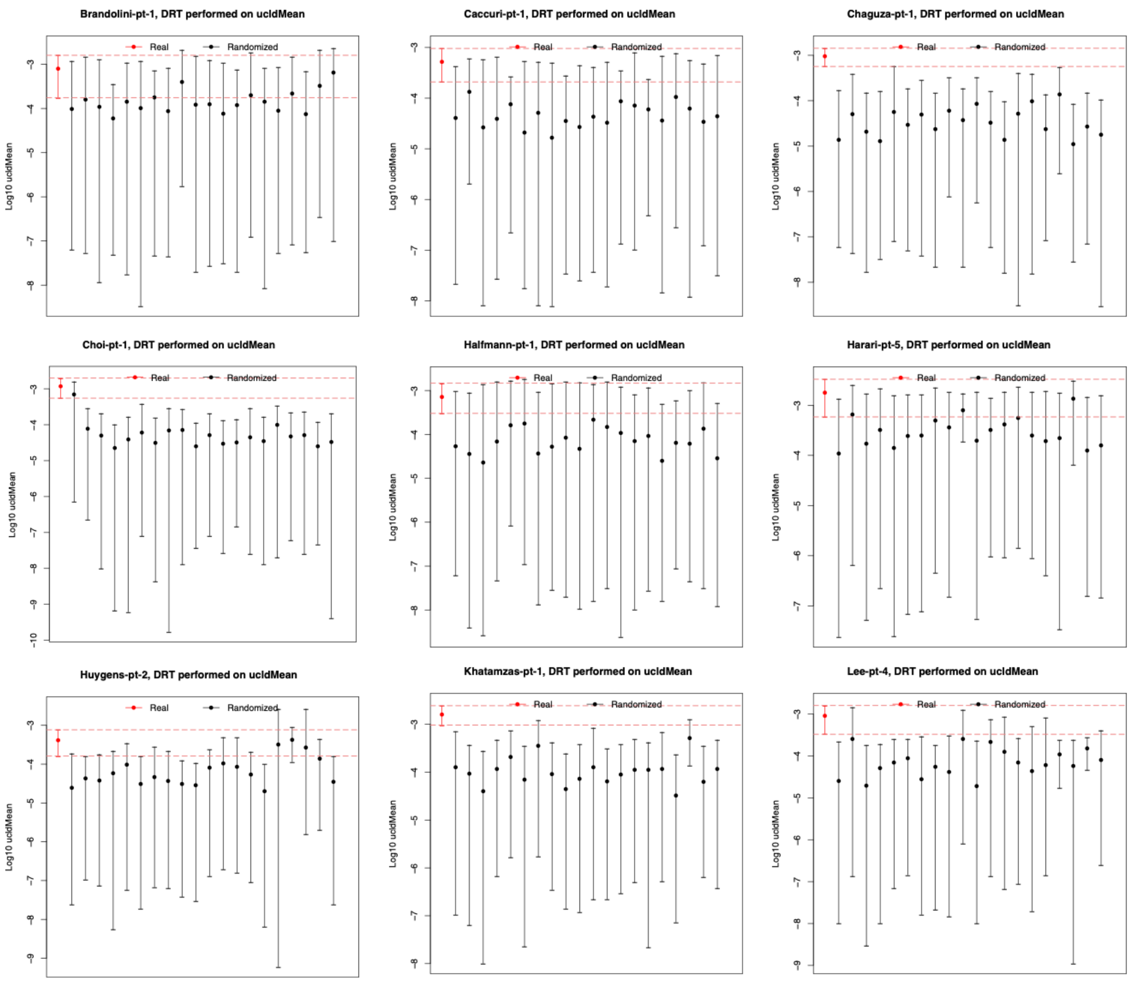

Supplementary figure S7. Date-randomisation test (DRT) performed on the clock rate parameter of the uncorrelated relaxed lognormal clock model. Each panel corresponds to estimates obtained from one dataset. Within each panel, an estimate indicated with red colour represents the real estimate whereas black colour denotes estimates obtained from date-randomized data sets. For each dataset date-randomization was performed twenty times. For clarity, on the Y axis evolutionary rate estimates are reported on a logarithmic scale. Overlapping 95% highest posterior density (HPD) distributions of real and randomized estimates might indicate that the strength of the temporal signal might not be sufficient enough to infer evolutionary rates with high confidence only based on tip-dating.

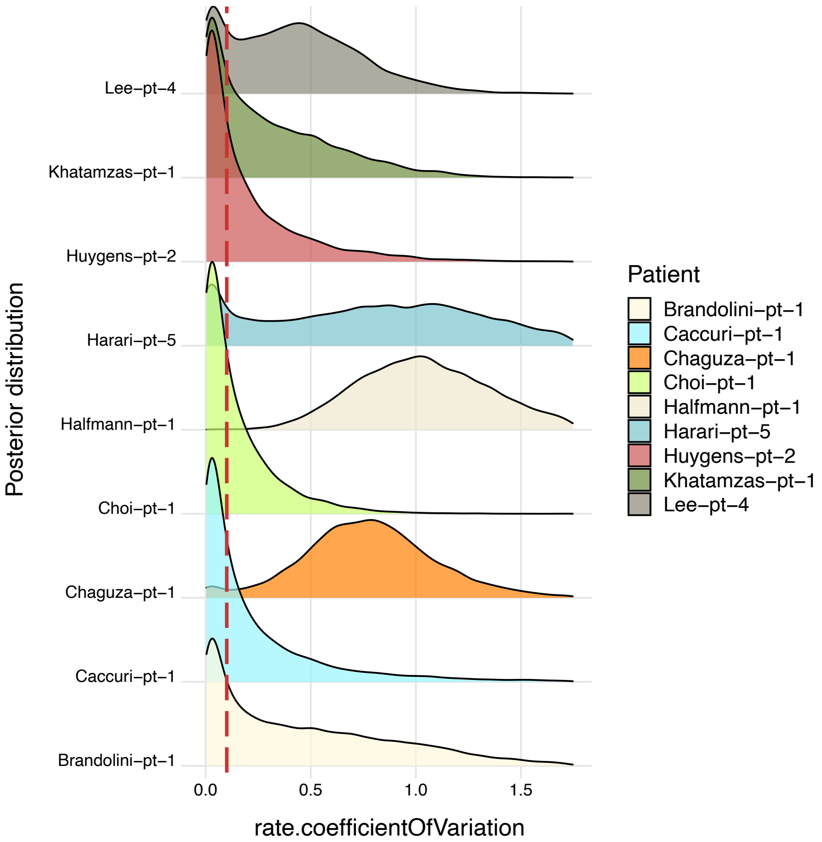

Supplementary figure S8. Marginal posterior distributions for coefficient of rate variation (uncorrelated lognormal relaxed clock model). This parameter characterises the clock-likeness of the data, and values closer to zero suggest that a strict clock model might describe the data better. Although no precise criteria have been established in the literature, concentration of marginal posterior distribution of coefficient of rate variation below the value 0.1 (indicated with red dashed line) can be considered sufficient to warrant the use of a strict clock model (Drummond and Bouckaert 2015). Posterior distributions for all nine datasets illustrate signals of non-clocklike evolution, rate variation among branches being pronounced especially in Chaguza-pt-1, Halfmann-pt-1, Harari-pt-5, Khatamzas-pt-1 and Lee-pt-4.

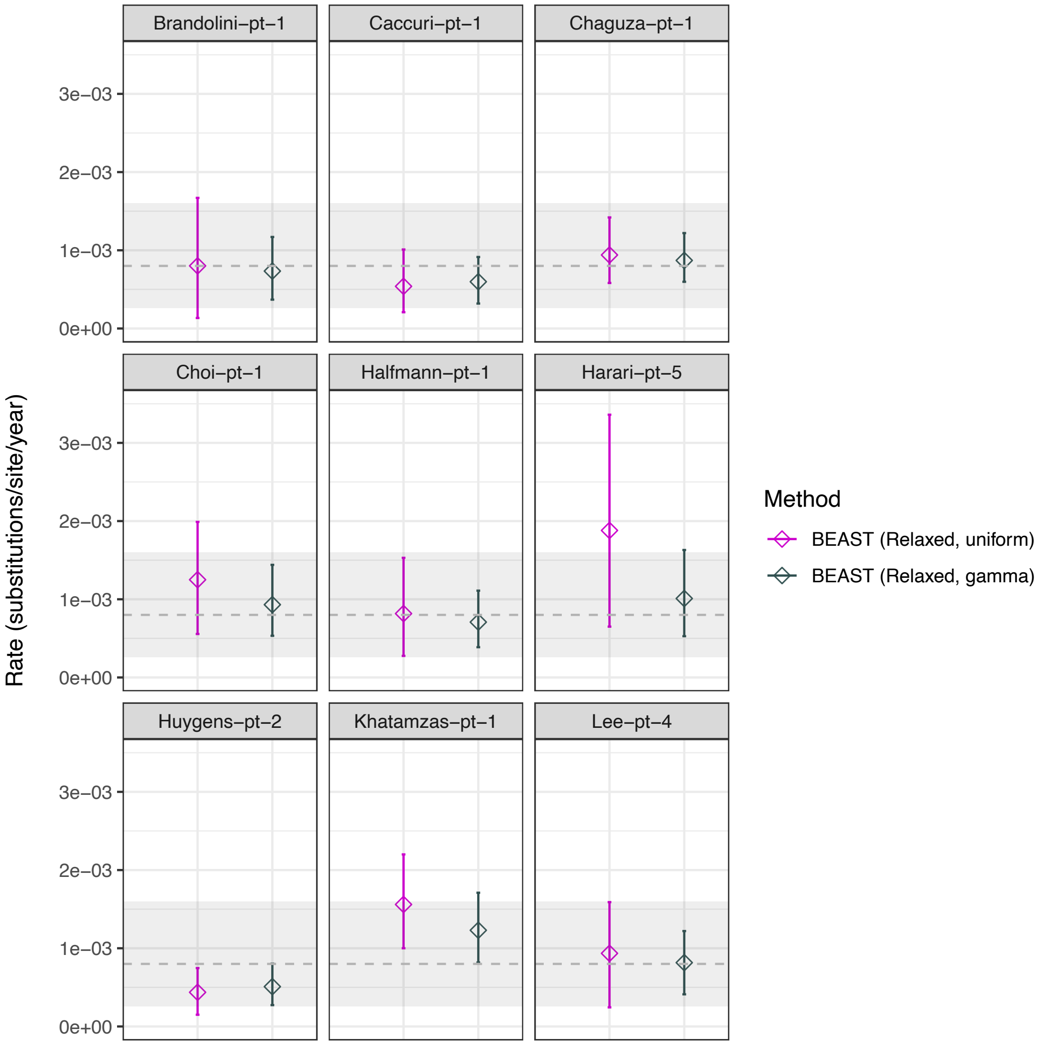

Supplementary figure S9. BEAST2 sensitivity analysis with a Gamma-distributed prior for the relaxed clock analyses. For the results presented in the main text, a normal distribution with mean of 0.0008 and standard deviation of 0.0016 was assumed as a prior distribution for the relaxed clock rate parameter. These sensitivity analyses assume a Gamma-distributed prior with shape parameter α=8.0 and rate parameter λ=10^4, yielding a mean rate of 8.0e-04 subst./site/year, with 95% of the probability density falling between 3.45e-04 and 14.4e-04 subst./site/year. Evolutionary rate estimates obtained using the originally applied normally-distributed prior and the Gamma-distributed prior are highly consistent. The grey dashed line represents the commonly used SARS-CoV-2 substitution rate estimate of 8.0e-04 subst./site/year (Ghafari et al. 2020). The grey shaded area denotes the lowest and highest mean evolutionary rate estimates for SARS-CoV-2 collected from previous publications (3e-04 – 16e-04 subst./site/year, see Supplementary table S12).

Supplementary text S2. BEAST2 sensitivity analysis with alternative tree prior models

For the Bayesian approach we chose to utilise as an underlying tree prior distribution a deterministic coalescent based Bayesian skyline plot (BSP) model (Drummond et al. 2005) over the birth-death-sampling models. Despite the latter being considered more suitable for processes with stochastic population size changes including the emergence of a viral outbreak (Boskova et al. 2014) modelling the within-host sampling process through time might be challenging, if not impossible. Given that poor characterization of the sampling process may lead to severely biased results within the birth-death-sampling framework (Volz and Frost 2014) we considered a coalescent based approach being less vulnerable for misspecified sampling schemes. Moreover, we would like to point out that a comprehensive Bayesian analysis would also involve proper model selection to evaluate the best-fit clock and tree prior models, as well as sample-from-prior analysis, as discussed for example in (Baele et al. 2012; Lartillot 2023). However, given the vast number of datasets and various combinations of clock (strict vs. relaxed) and tree prior models (BSP vs. coalescent constant population size vs. coalescent exponential growth) to be tested, we chose to omit these further steps. Nonetheless, since misspecified tree prior may lead to increased rate estimates (Möller et al. 2018), we performed additional analyses for Chaguza-pt-1 and Khatamzas-pt-1 with coalescent constant size and coalescent exponential population growth models to ensure that elevated BEAST2 estimates are not a product of a tree prior used. Rate estimates inferred with these two additional tree prior models are greatly similar to estimates derived with BSP, as shown in Supplementary figure S9.

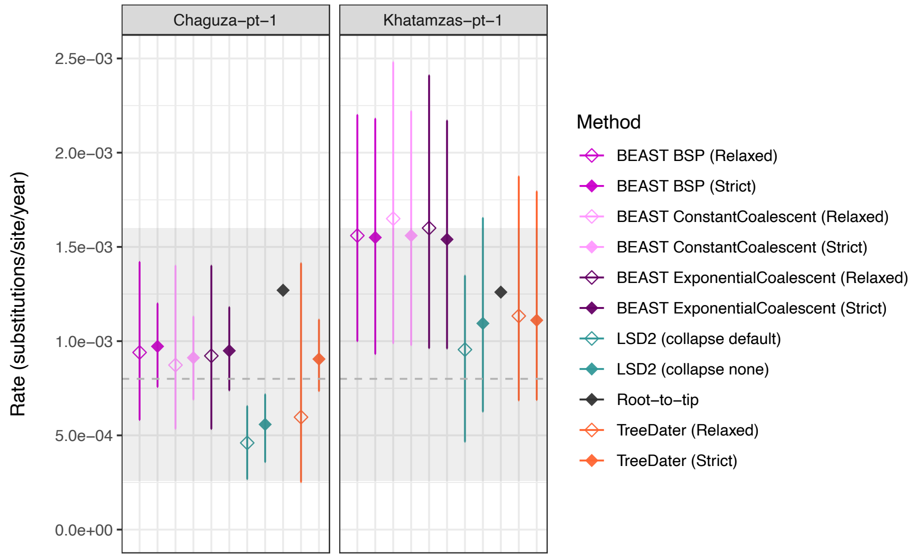

Supplementary figure S10. Rate estimates for Chaguza-pt-1 and Khatamzas-pt-1 obtained with alternative tree priors. For the results presented in the main text, for BEAST2 analysis the Bayesian skyline plot (BSP) model was used as an underlying tree prior. For Chaguza-pt-1 and Khatamzas-pt-1 we performed additional analysis by assuming coalescent constant size and coalescent exponential population growth models. For both tree priors, runs were executed by assuming strict and uncorrelated lognormal relaxed clock models. Results show that tree priors do not have a notable impact on the evolutionary rate estimates inferred. For details, see Supplementary text S2. Grey dashed line represents the commonly used SARS-CoV-2 substitution rate estimate of 8.0e-04 subst./site/year (Ghafari et al. 2020). The grey shaded area denotes the lowest and highest mean evolutionary rate estimates for SARS-CoV-2 collected from various publications (3e-04 – 16e-04 subst./site/year, see Supplementary table S11).

Supplementary table S14. Topological distances between pairs of phylogenetic trees. For each dataset, three comparisons were performed with R package TreeDist: LSD2 vs. BEAST2 strict clock MCC tree, LSD2 vs. BEAST2 relaxed clock MCC tree, and BEAST2 strict clock MCC tree vs. BEAST2 relaxed clock MCC tree. According to (Smith 2020), 'SharedPhylogeneticInfo' metrics describes the amount of phylogenetic information in common between two trees, whereas 'DifferentPhylogeneticInfo' metrics describes the distance between trees under scrutiny i.e. how much information is different in the splits of these two trees. Regarding LSD2, comparisons were performed with trees reconstructed by allowing zero length branches and collapsing short branches. Results for the latter are presented in parenthesis. When 'DifferentPhylogeneticInfo' yielded a value of 0, trees were considered identical. When the score for shared splits exceeded the score for conflicting splits ('SharedPhylogeneticInfo' > 'DifferentPhylogeneticInfo'), two trees were considered to exhibit modest variation in the tree topology. When the score for conflicting splits exceeded the score for shared splits ('SharedPhylogeneticInfo' < 'DifferentPhylogeneticInfo'), trees were considered to exhibit notable variation in the tree topology (highlighted with red colour).

Dataset	LSD2 vs. BEAST2 strict		LSD2 vs. BEAST2 relaxed		BEAST2 strict vs. BEAST2 relaxed	
	Shared	Different	Shared	Different	Shared	Different
Brandolini-pt-1	20.47 (13.55)	0.0 (6.92)	14.98 (13.55)	13.05 (8.99)	14.98	13.05
Caccuri-pt-1	29.68 (20.22)	63.66 (46.41)	29.68 (20.22)	63.66 (46.41)	62.39	8.49
Chaguza-pt-1	291.74 (261.42)	210.76 (192.05)	295.06 (262.86)	191.82 (176.87)	389.60	32.14
Choi-pt-1	23.09 (23.09)	10.12 (3.70)	23.09 (23.09)	10.12 (3.70)	26.79	0.0
Halfmann-pt-1	48.39 (47.89)	18.49 (10.99)	48.39 (47.89)	18.49 (10.99)	58.89	0.0
Harari-pt-5	23.18 (16.54)	16.42 (14.85)	26.35 (16.54)	10.08 (14.85)	27.69	7.40
Huygens-pt-2	41.95 (34.25)	67.59 (48.41)	41.95 (34.25)	67.59 (48.41)	75.75	0.0
Khatamzas-pt-1	186.34 (116.89)	80.01 (127.85)	186.34 (116.89)	80.01 (127.85)	237.99	0.0
Lee-pt-4	19.36 (19.36)	0.0 (0.0)	19.36 (19.36)	0.0 (0.0)	19.36	0.0

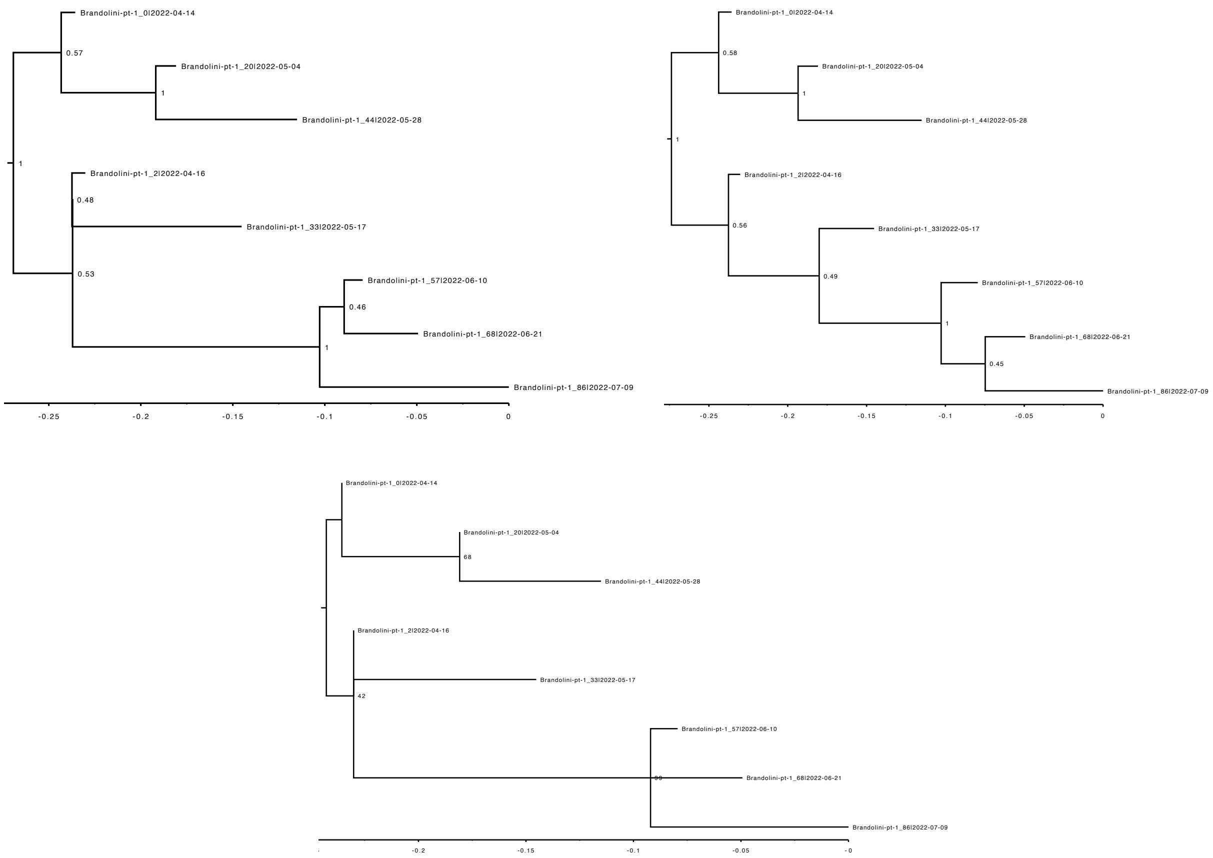

Supplementary figure S11. Time-trees for Brandolini-pt-1. In the upper panel maximum clade credibility (MCC) trees from BEAST2 strict (left) and relaxed (right) clock analysis are given. In the lower panel a maximum likelihood tree generated with LSD2 is given. For the LSD2 tree, internal branches having branch length less than 1.67e-05 (= 0.5/sequence length) were collapsed. For BEAST2 trees node posterior support values are presented, for LSD2 bootstrap values.

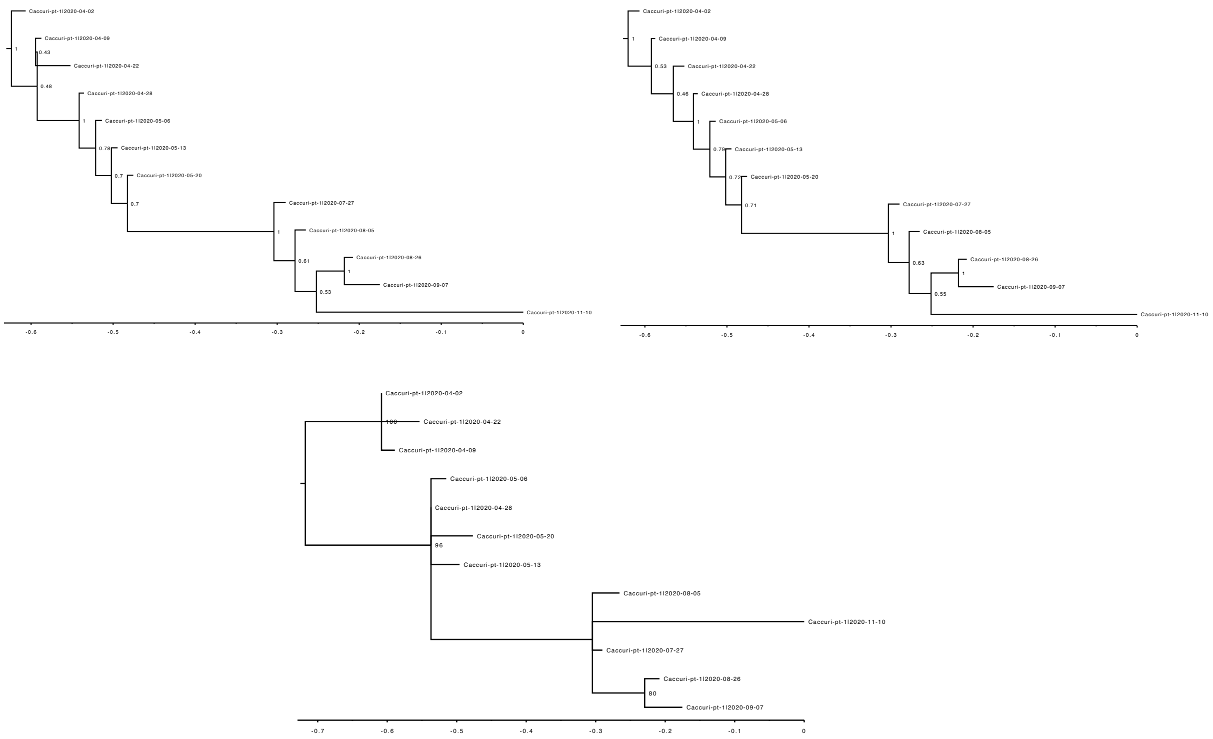

Supplementary figure S12. Time-trees for Caccuri-pt-1. In the upper panel maximum clade credibility (MCC) trees from BEAST2 strict (left) and relaxed (right) clock analysis are given. In the lower panel a maximum likelihood tree generated with LSD2 is given. For the LSD2 tree, internal branches having branch length less than 1.67e-05 (= 0.5/sequence length) were collapsed. For BEAST2 trees node posterior support values are presented, for LSD2 bootstrap values.

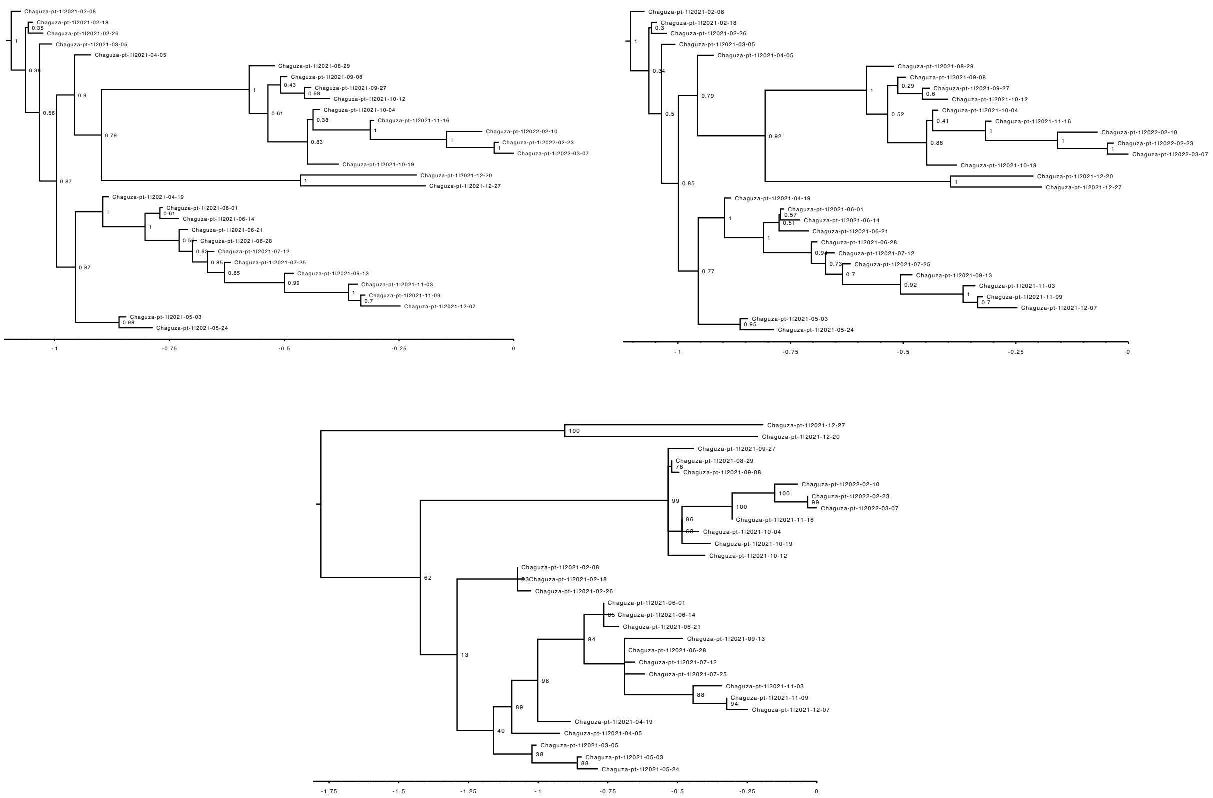

Supplementary figure S13. Time-trees for Chaguza-pt-1. In the upper panel maximum clade credibility (MCC) trees from BEAST2 strict (left) and relaxed (right) clock analysis are given. In the lower panel a maximum likelihood tree generated with LSD2 is given. For the LSD2 tree, internal branches having branch length less than 1.67e-05 (= 0.5/sequence length) were collapsed. For BEAST2 trees node posterior support values are presented, for LSD2 bootstrap values.

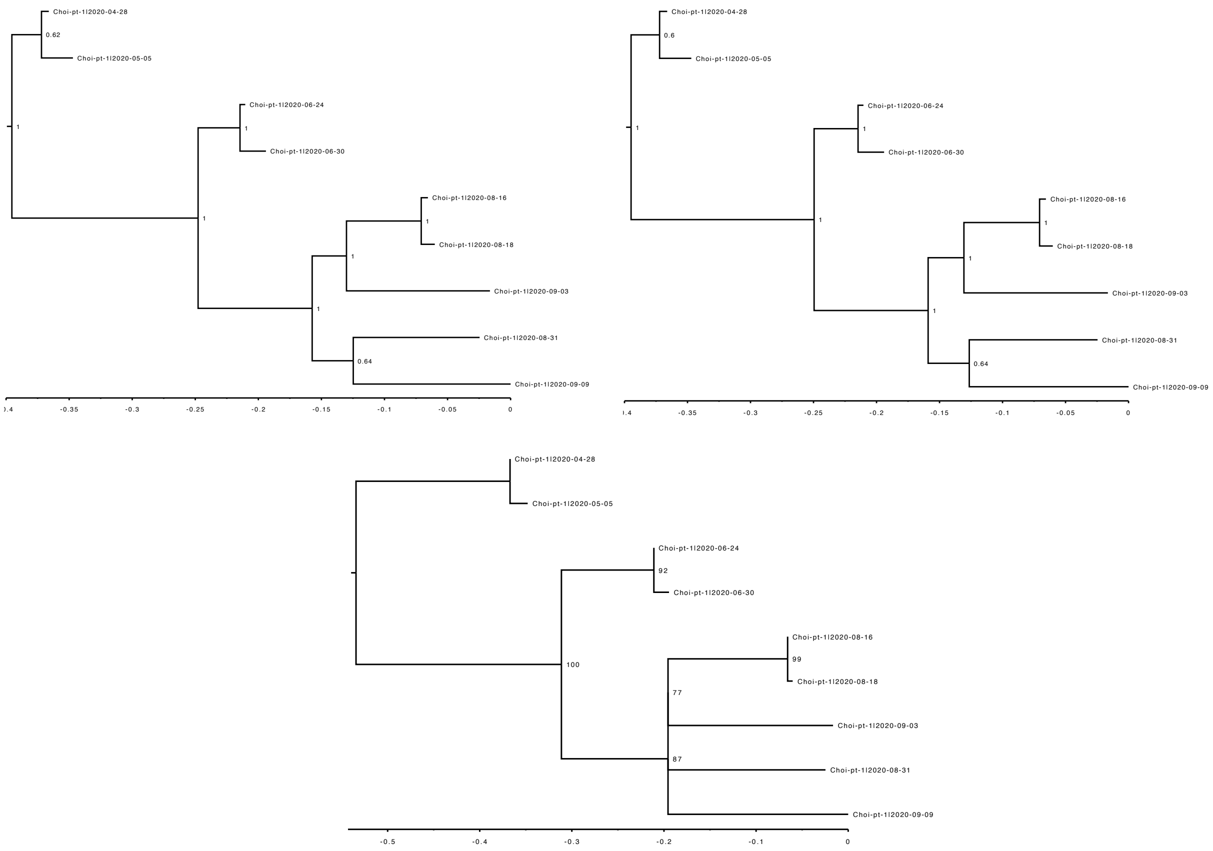

Supplementary figure S14. Time-trees for Choi-pt-1. In the upper panel maximum clade credibility (MCC) trees from BEAST2 strict (left) and relaxed (right) clock analysis are given. In the lower panel a maximum likelihood tree generated with LSD2 is given. For the LSD2 tree, internal branches having branch length less than 1.67e-05 (= 0.5/sequence length) were collapsed. For BEAST2 trees node posterior support values are presented, for LSD2 bootstrap values.

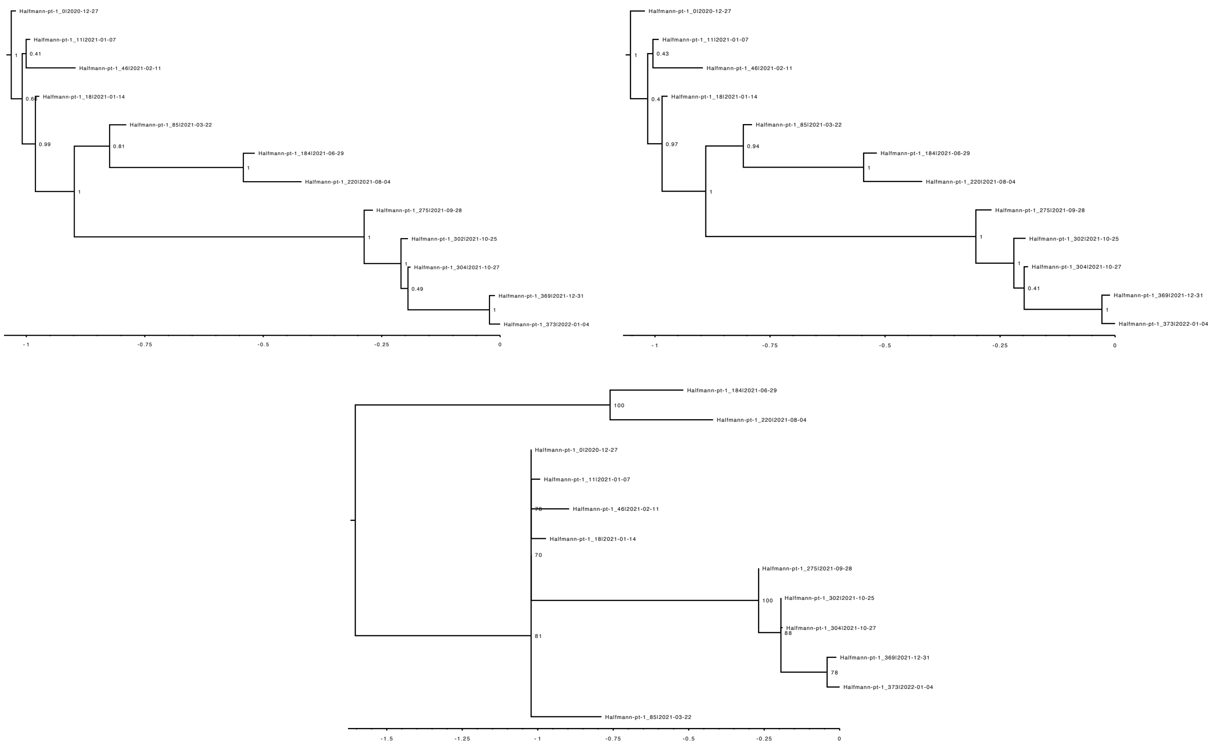

Supplementary figure S15. Time-trees for Halfmann-pt-1. In the upper panel maximum clade credibility (MCC) trees from BEAST2 strict (left) and relaxed (right) clock analysis are given. In the lower panel a maximum likelihood tree generated with LSD2 is given. For the LSD2 tree, internal branches having branch length less than 1.67e-05 (= 0.5/sequence length) were collapsed. For BEAST2 trees node posterior support values are presented, for LSD2 bootstrap values.

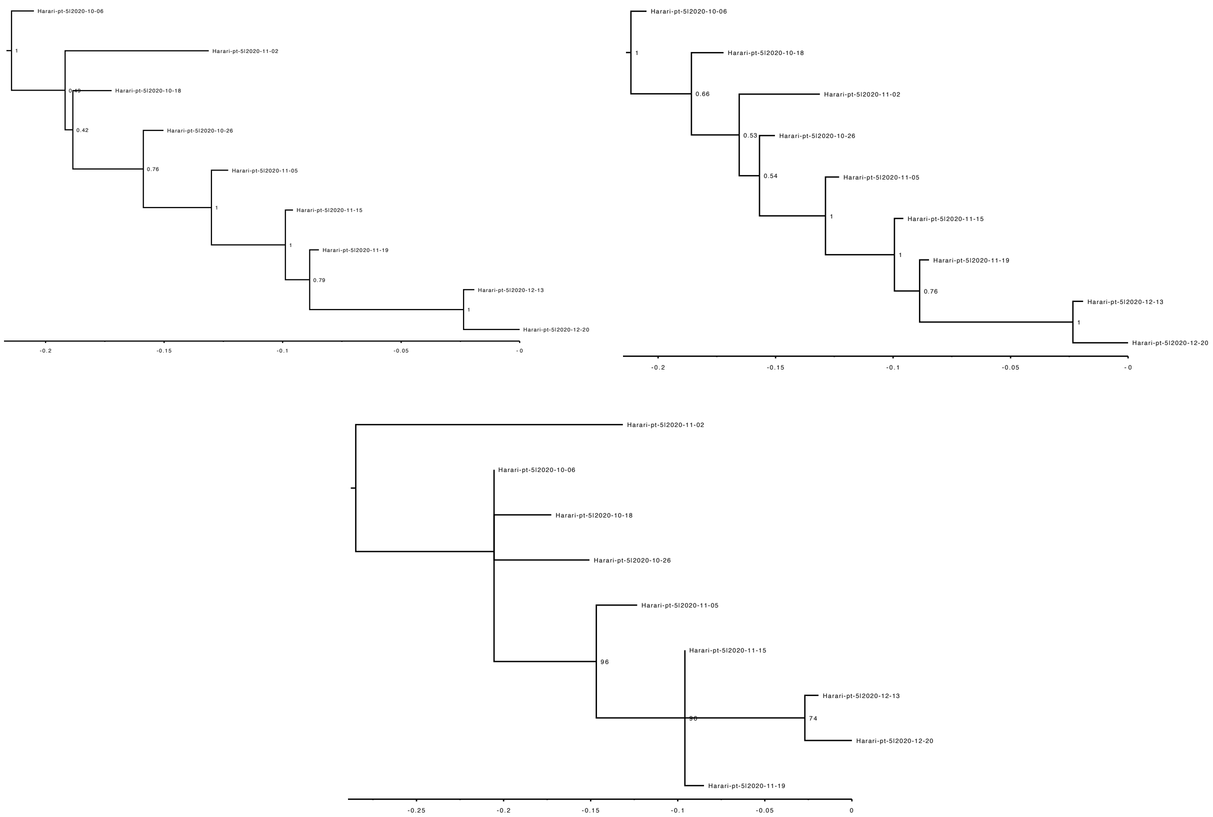

Supplementary figure S16. Time-trees for Harari-pt-5. In the upper panel maximum clade credibility (MCC) trees from BEAST2 strict (left) and relaxed (right) clock analysis are given. In the lower panel a maximum likelihood tree generated with LSD2 is given. For the LSD2 tree, internal branches having branch length less than 1.67e-05 (= 0.5/sequence length) were collapsed. For BEAST2 trees node posterior support values are presented, for LSD2 bootstrap values.

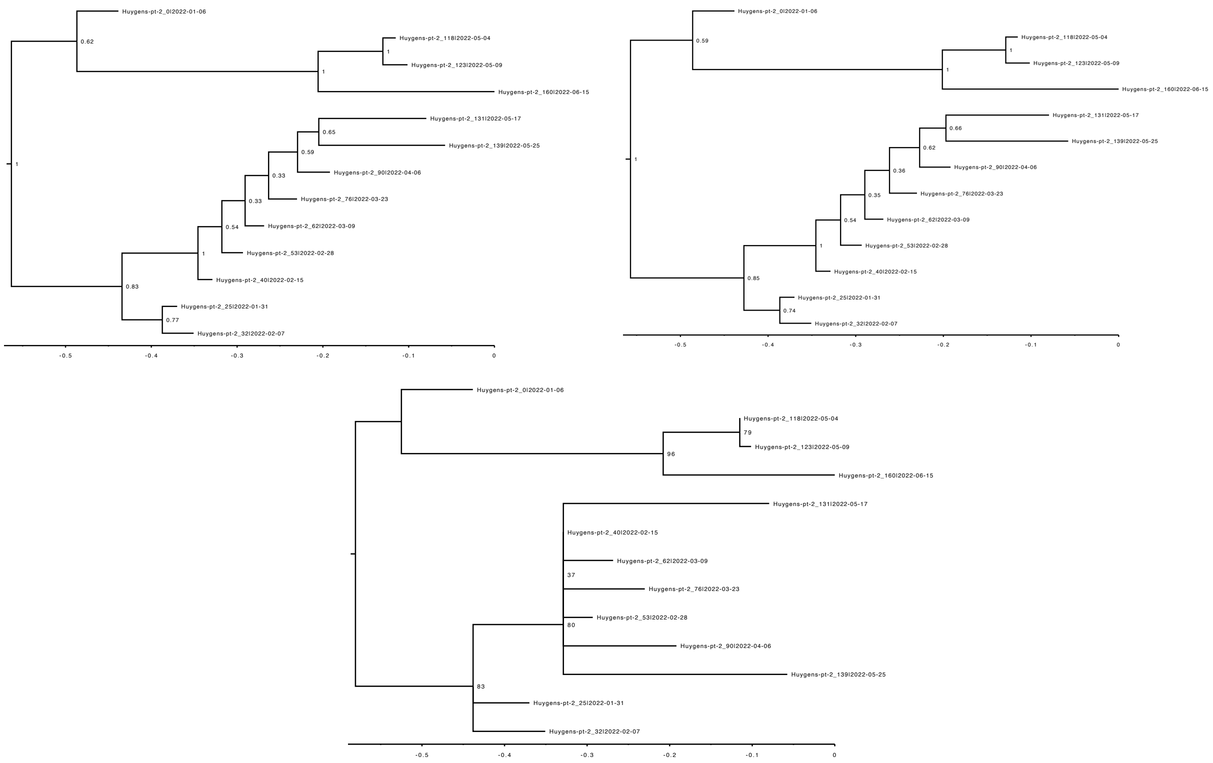

Supplementary figure S17. Time-trees for Huygens-pt-2. In the upper panel maximum clade credibility (MCC) trees from BEAST2 strict (left) and relaxed (right) clock analysis are given. In the lower panel a maximum likelihood tree generated with LSD2 is given. For the LSD2 tree, internal branches having branch length less than 1.67e-05 (= 0.5/sequence length) were collapsed. For BEAST2 trees node posterior support values are presented, for LSD2 bootstrap values. The tree topology revealed a notable substructure of the viral population. Whereas the first sequence for the datasets was obtained on the same day as the reported onset of symptoms (2022-01-06), the median estimates for the tree height date two months earlier with both clock models (2021-11-07). Similar estimates for the most recent common ancestor were obtained with LSD2 (collapse none: 2021-11-03, collapse default: 2021-11-15), and TreeDater yielded even older estimates (strict clock: 2021-08-18, relaxed clock: 2021-09-17). Based on this, it is plausible that the patient has been superinfected with two SARS-CoV-2 strains representing the same Pango lineage (BA.1.1).

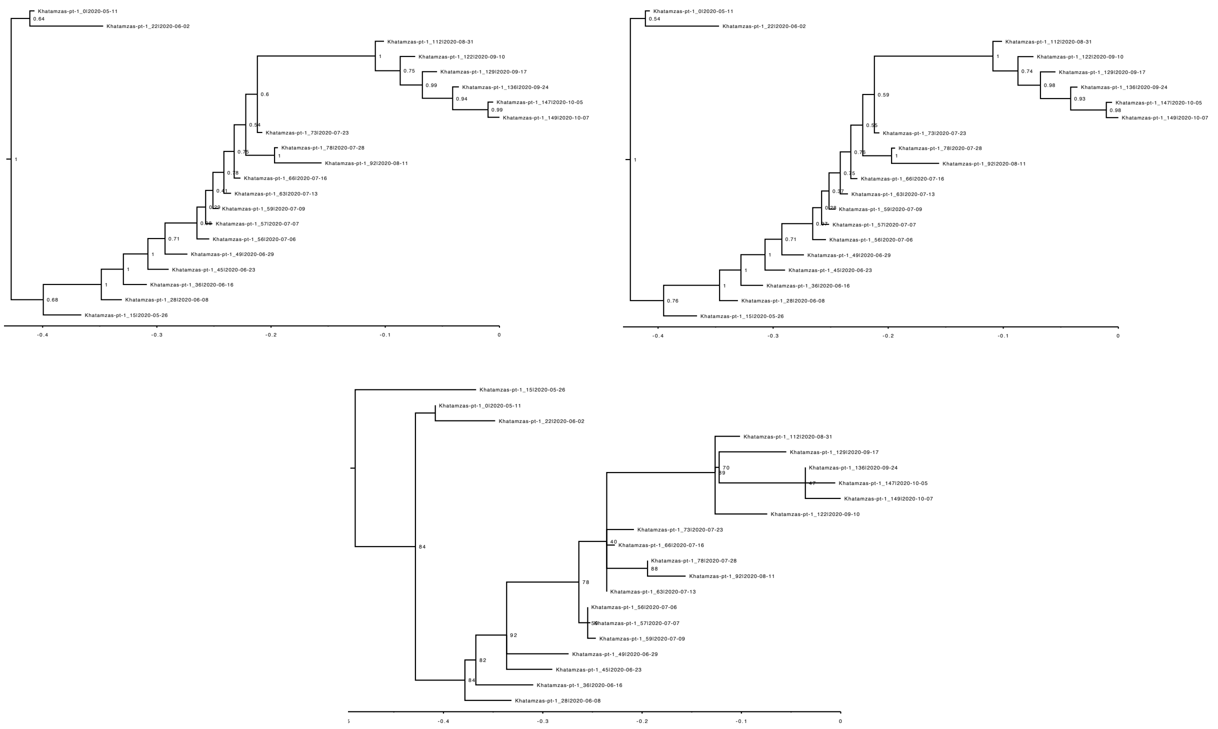

Supplementary figure S18. Time-trees for Khatamzas-pt-1. In the upper panel maximum clade credibility (MCC) trees from BEAST2 strict (left) and relaxed (right) clock analysis are given. In the lower panel a maximum likelihood tree generated with LSD2 is given. For the LSD2 tree, internal branches having branch length less than 1.67e-05 (= 0.5/sequence length) were collapsed. For BEAST2 trees node posterior support values are presented, for LSD2 bootstrap values.

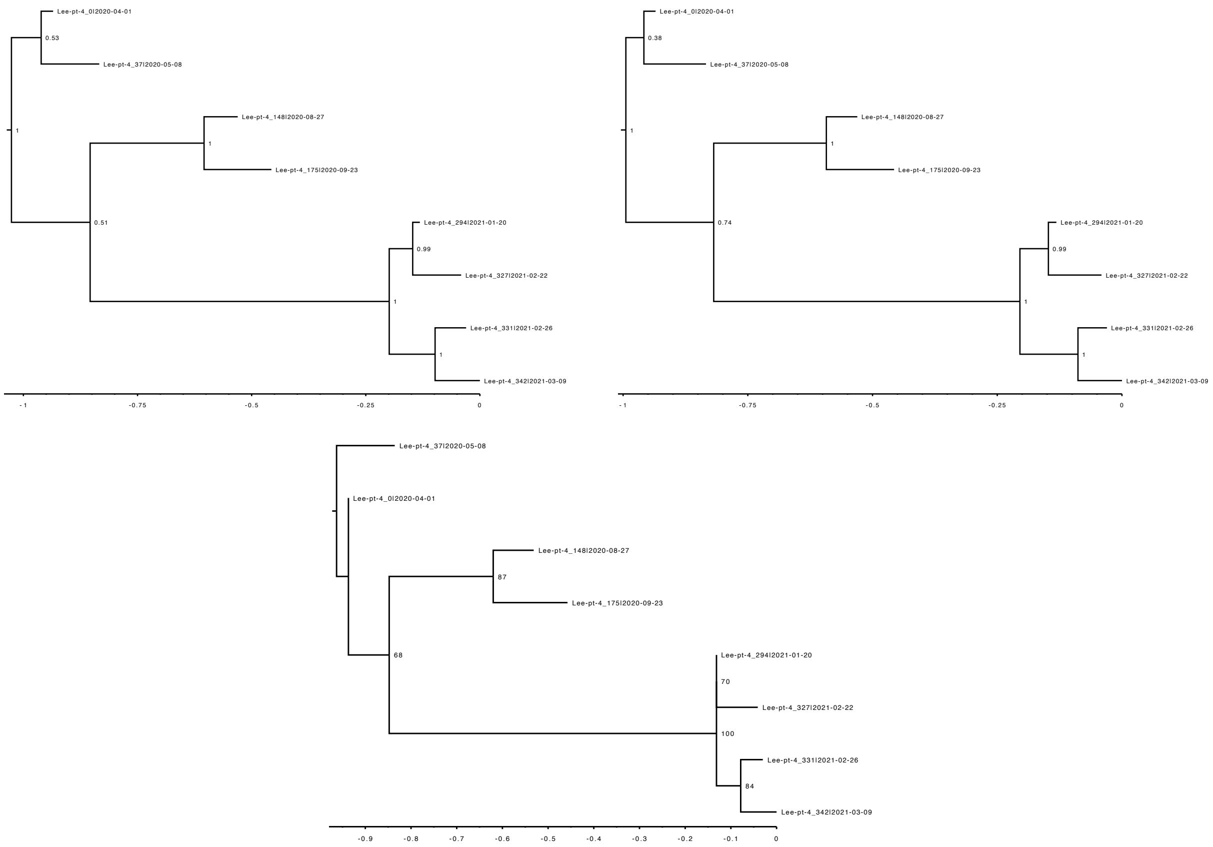

Supplementary figure S19. Time-trees for Lee-pt-4. In the upper panel maximum clade credibility (MCC) trees from BEAST2 strict (left) and relaxed (right) clock analysis are given. In the lower panel a maximum likelihood tree generated with LSD2 is given. For the LSD2 tree, internal branches having branch length less than 1.67e-05 (= 0.5/sequence length) were collapsed. For BEAST2 trees node posterior support values are presented, for LSD2 bootstrap values.

Supplementary table S15. Results from the PhyloTempo analysis performed for nine datasets. The temporal clustering (TC) statistics can get values between 0 and 1, TC=0 indicating a complete absence of temporal clustering (Gray et al. 2011; Norström et al. 2012). In (Gray et al. 2011) TC values of ~ 0.3 and above are considered to indicate a high degree of TC. 'Staircase-ness' statistic describes the proportion of imbalanced subtrees and values of zero indicate a perfectly balanced binary tree whereas values of one indicate a perfectly imbalanced tree (Norström et al. 2012). Degree of temporal clustering was considered as 'Unresolved' for those datasets for which TC scores obtained from three independent runs were highly divergent. Under the TC scores, the optimal number of time intervals as well as number of leaves assigned to each bin, are reported for each parallel run.

Dataset	Number of tips	TC score	Staircase-ness	Degree of temporal clustering
Brandolini-pt-1	8	0.4137 / 0.0971 / 0.4036 (3-2-3 / 3-3-2 / 3-2-3)	0.5714	Unresolved
Caccuri-pt-1	12	0.007 / 0.0064 / 0.0088 (4-4-4)	0.5455	Low
Chaguza-pt-1	30	0.2931 / 0.2979 / 0.2971 (6-6-6-6-6)	0.7241	High
Choi-pt-1	9	0.0674 / 0.0734 / 0.0751 (3-3-3)	0.625	Low
Halfmann-pt-1	12	0.3981 / 0.3952 / 0.3952 (4-4-4)	0.5455	High
Harari-pt-5	9	0.2782 / 0.2879 / 0.2942 (3-3-3)	0.75	High
Huygens-pt-2	13	0.1366 / 0.1652 / 0.3083 (3-3-3-4 / 4-3-3-3 / 3-4-3-3)	0.75	Unresolved
Khatamzas-pt-1	21	0.5373 / 0.5566 / 0.5543 (5-4-4-4-4 / 4-4-4-5-4 / 4-4-4-5-4)	0.8	High
Lee-pt-4	8	0.2402 / 0.4411 / 0.2385 (2-3-3 / 3-3-2 / 2-3-3)	0.4286	Unresolved

Supplementary table S16. Results from Z-test of positive selection. Table cells represent the test statistic (d_N - d_S) and green colour demonstrates statistically significant indication of positive selection (i.e. p values < 0.05). * All = ORF1ab, S, E, M and N.

**Dataset**	**ORF1ab**	**S**	**E**	**M**	**N**	**All***
Brandolini-pt-1	-0.82	1.80	0.00	0.00	0.00	-0.24
Caccuri-pt-1	-1.23	0.08	-0.92	0.00	0.00	-1.06
Chaguza-pt-1	-1.29	1.87	0.95	1.20	0.02	-0.07
Choi-pt-1	-1.48	2.30	0.92	1.36	1.040	0.74
Halfmann-pt-1	-1.03	2.10	1.08	0.00	-0.05	-0.11
Harari-pt-5	-0.14	0.67	0.00	0.00	-0.31	0.38
Huygens-pt-2	-1.25	1.32	0.00	-0.96	0.00	0.05
Khatamzas-pt-1	0.46	1.22	0.00	0.00	1.39	1.27
Lee-pt-4	2.41	0.54	1.30	0.49	0.00	2.36

Supplementary text S3. BEAST2 sensitivity analysis with fixed rate for 'Patient case histories'

As the BEAST2 estimates appeared biased towards higher rates, we further evaluated if the observed temporal oscillations in evolutionary rates hold when fixing the mean rate of the relaxed clock model to a commonly used substitution rate reference estimate of 8.0e-04 subst./site/year. As shown in Supplementary figures S19–S25, when the inferred mean rate estimate is close to the fixed rate used, patterns of rate changes through time are highly similar between trees with fixed and unfixed clock rates (Brandolini-pt-1, Chaguza-pt-1 and Halfmann-pt-1). Conversely, when the inferred rate estimate is somewhat lower or higher than the fixed rate, minor scale differences can be detected between the corresponding trees (Choi-pt-1, Harari-pt-5, Huygens-pt-2 and Khatamzas-pt-1). Nonetheless, the broad patterns of evolutionary rate changes remain comparable, allowing for further examinations of temporal concurrencies.

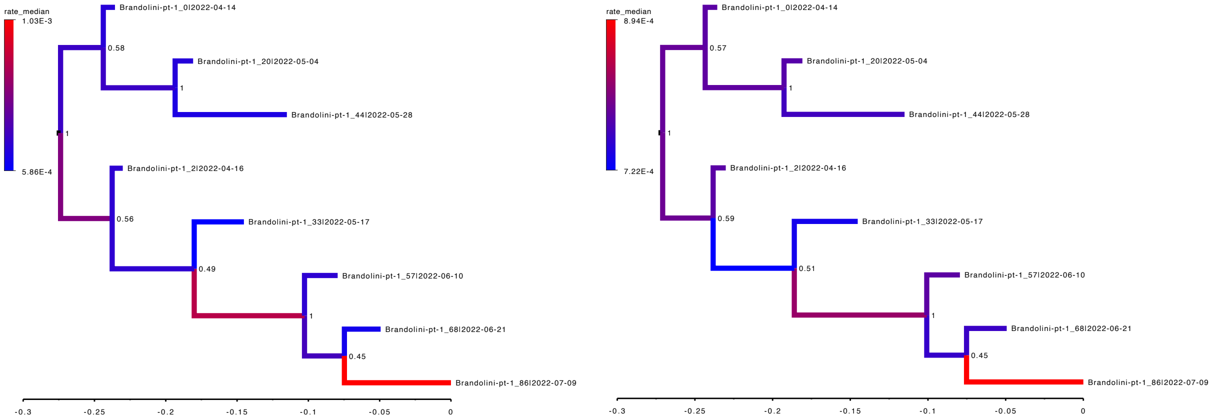

Supplementary figure S20. Impact of fixing the mean rate of relaxed clock analysis for Brandolini-pt-1. In the left, mean rate is estimated with prior N(0.0008, 0.0016) and in the right mean rate is fixed to 8.00e-04 substitutions/site/year.

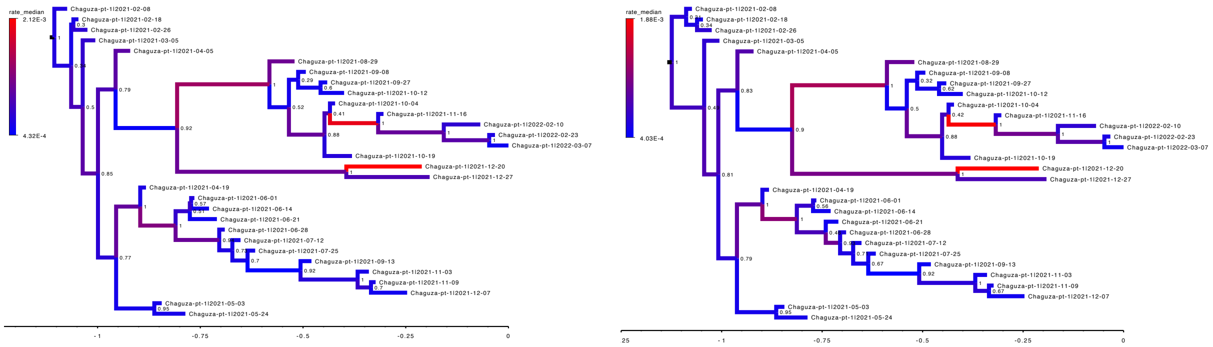

Supplementary figure S21. Impact of fixing the mean rate of relaxed clock analysis for Chaguza-pt-1. In the left, mean rate is estimated with prior N(0.0008, 0.0016) and in the right mean rate is fixed to 8.00e-04 substitutions/site/year.

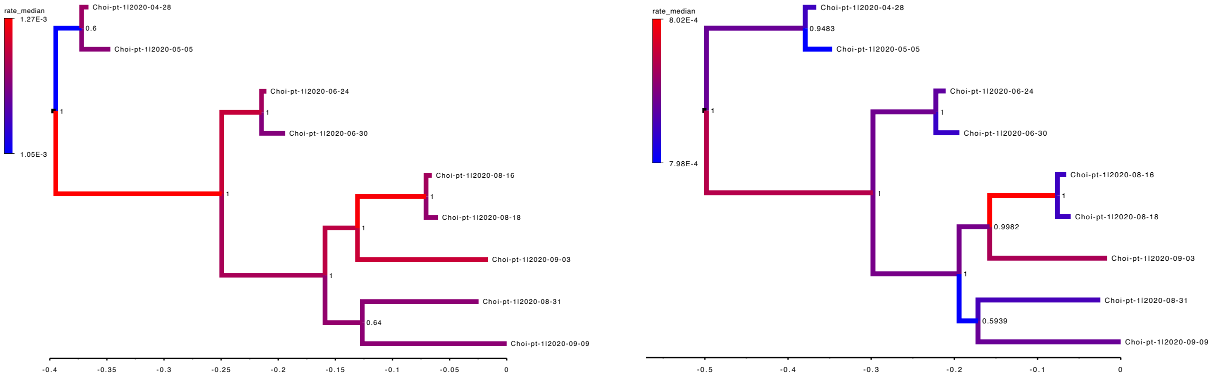

Supplementary figure S22. Impact of fixing the mean rate of relaxed clock analysis for Choi-pt-1. In the left, mean rate is estimated with prior N(0.0008, 0.0016) and in the right mean rate is fixed to 8.00e-04 substitutions/site/year.

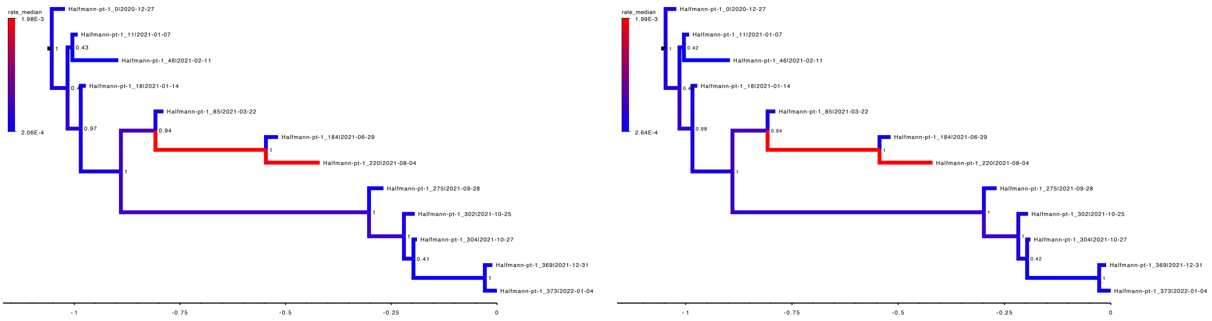

Supplementary figure S23. Impact of fixing the mean rate of relaxed clock analysis for Halfmann-pt-1. In the left, mean rate is estimated with prior N(0.0008, 0.0016) and in the right mean rate is fixed to 8.00e-04 substitutions/site/year.

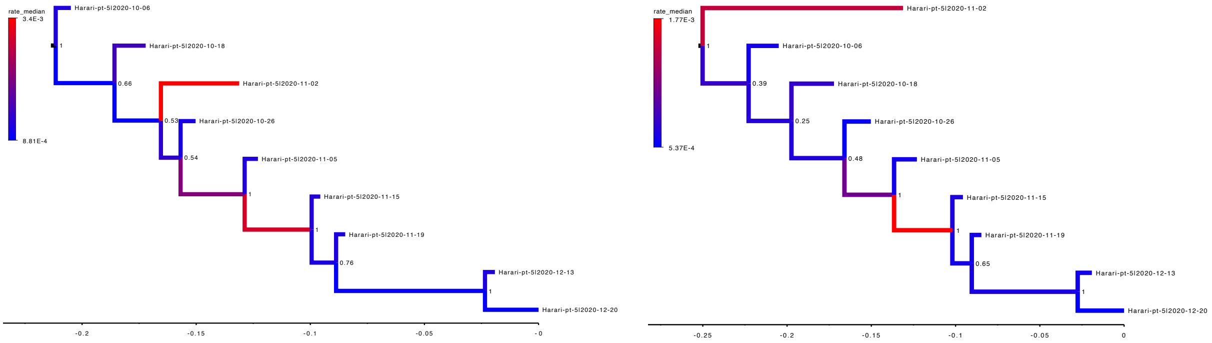

Supplementary figure S24. Impact of fixing the mean rate of relaxed clock analysis for Harari-pt-5. In the left, mean rate is estimated with prior N(0.0008, 0.0016) and in the right mean rate is fixed to 8.00e-04 substitutions/site/year.

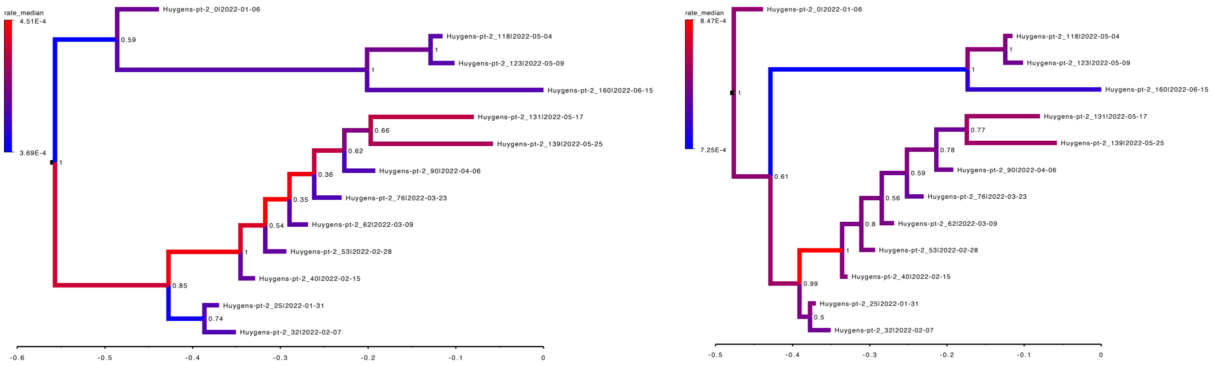

Supplementary figure S25. Impact of fixing the mean rate of relaxed clock analysis for Huygens-pt-2. In the left, mean rate is estimated with prior N(0.0008, 0.0016) and in the right mean rate is fixed to 8.00e-04 substitutions/site/year.

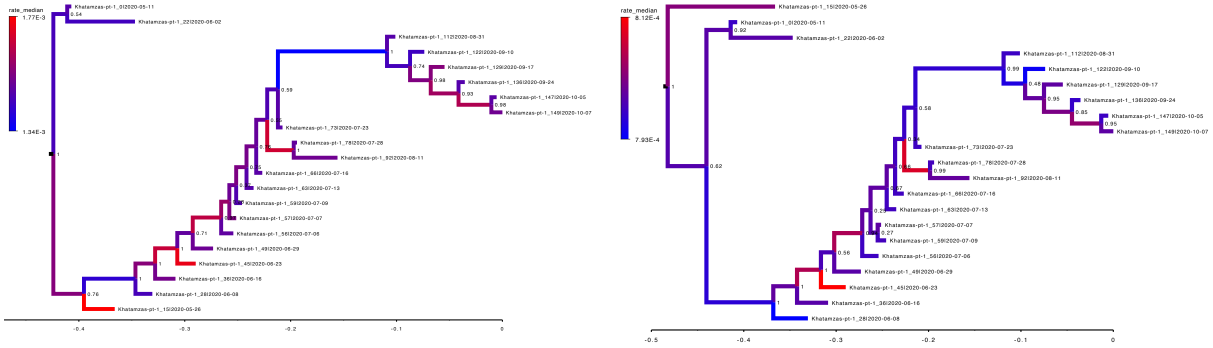

Supplementary figure S26. Impact of fixing the mean rate of relaxed clock analysis for Khatamzas-pt-1. In the left, mean rate is estimated with prior N(0.0008, 0.0016) and in the right mean rate is fixed to 8.00e-04 substitutions/site/year.

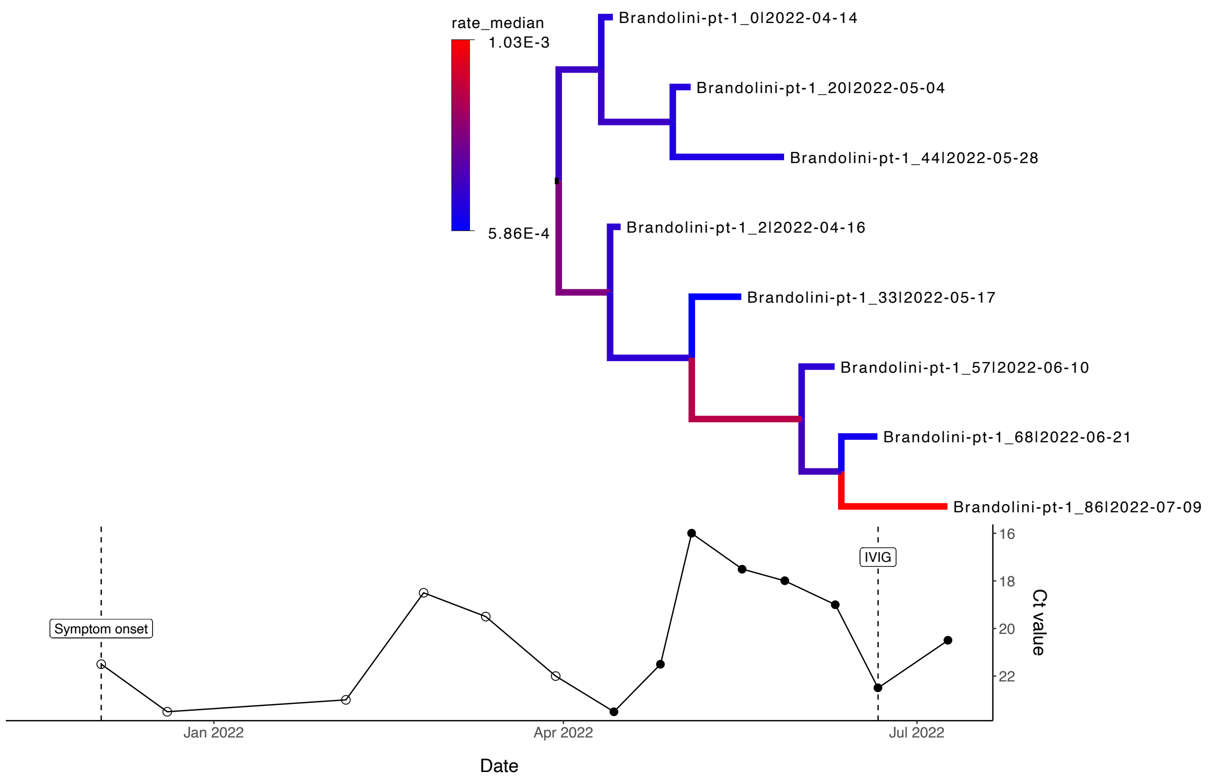

Supplementary figure S27. Patient case history for Brandolini-pt-1 patient, with follicular lymphoma as underlying clinical condition. Figure describes through time the changes in the evolutionary rates (by assuming an uncorrelated lognormal relaxed clock model), Ct values and SARS-CoV-2 treatments administered within the sampling window. For Brandolini-pt-1 the first viral sequence was obtained 132 days after the onset of symptoms. Patient was treated with intravenous immunoglobulin (IVIG) which targets spike-protein and has a half-time of approximately 26 days with notable variation. Colouring of the branches within the phylogenetic tree represents evolutionary rate estimates (in substitutions/site/year) obtained with BEAST2, lower values indicated with blue and higher rates with red colour. Open circles denote samples for which only Ct values were available and coloured circles denote samples which were sequenced.

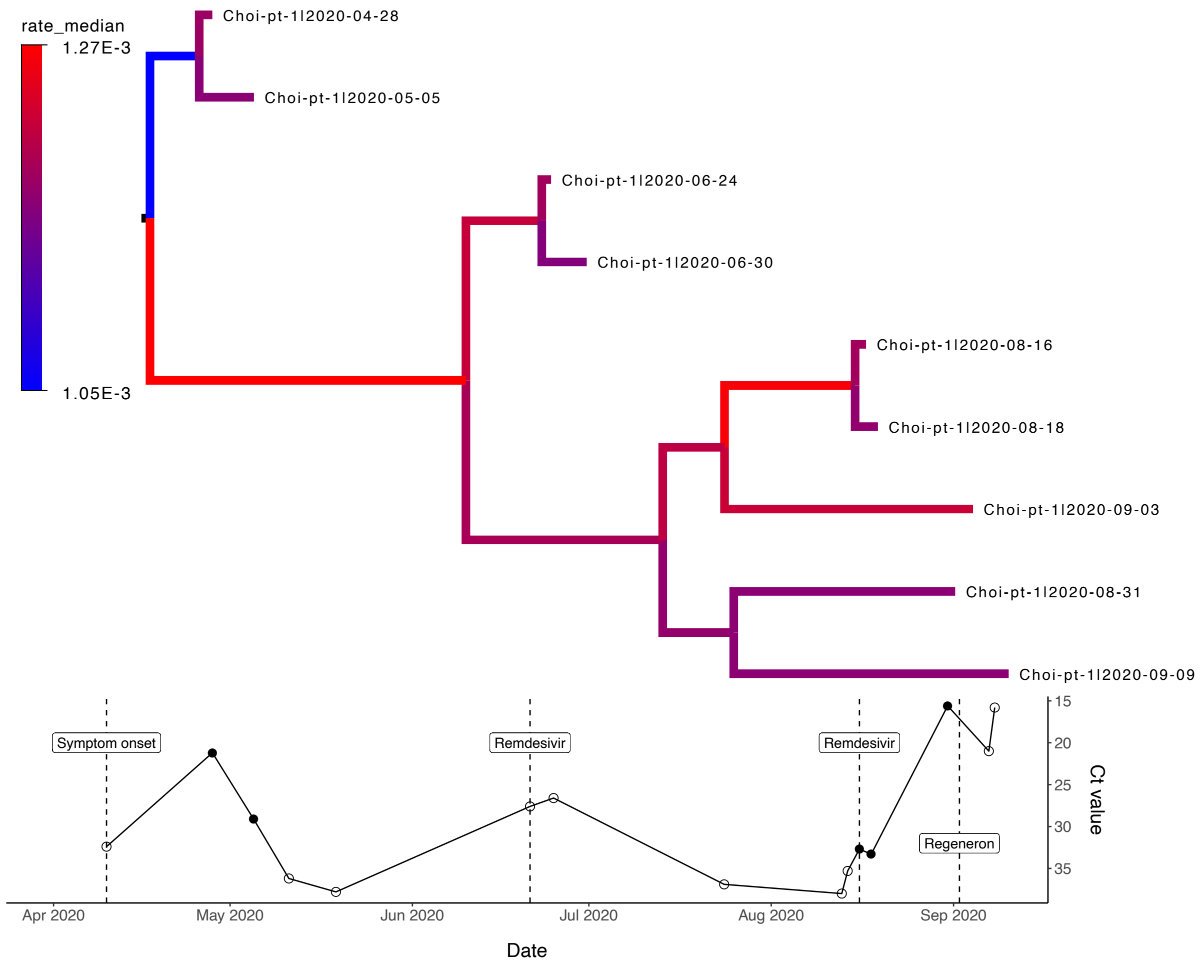

Supplementary figure S28. Patient case history for Choi-pt-1 patient, with catastrophic antiphospholipid syndrome (CAPS) as underlying clinical condition. Figure describes through time the changes in the evolutionary rates (by assuming an uncorrelated lognormal relaxed clock model), Ct values and SARS-CoV-2 treatments administered within the sampling window. For Choi-pt-1 the first viral sequence was obtained 18 days after the onset of symptoms. Patient was treated twice with Remdesivir which targets polymerase and has a half-time of approximately 17 hours. Patient was also treated with an antibody cocktail against SARS-CoV-2 (Regeneron, (Baum et al. 2020)). Colouring of the branches within the phylogenetic tree represents evolutionary rate estimates (in substitutions/site/year) obtained with BEAST2, lower values indicated with blue and higher rates with red colour. Open circles denote samples for which only Ct values were available and coloured circles denote samples which were sequenced.

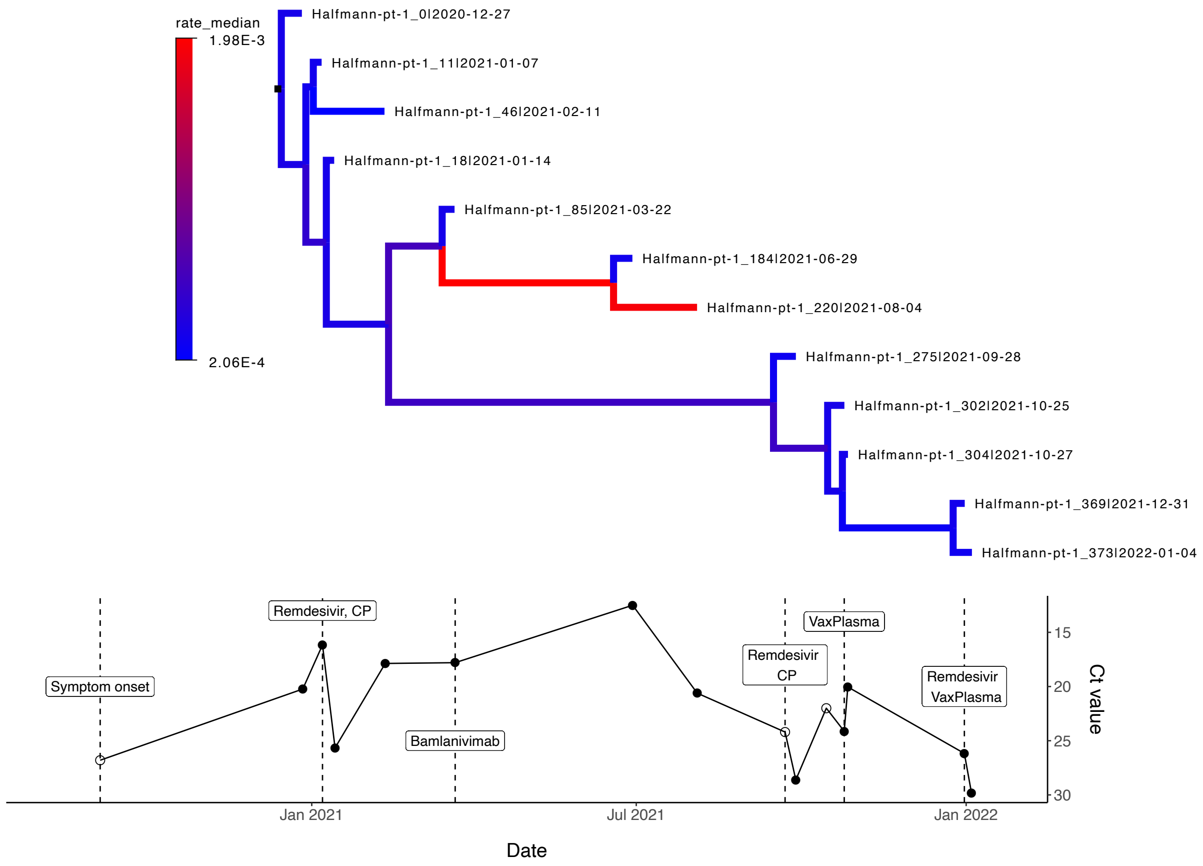

Supplementary figure S29. Patient case history for Halfmann-pt-1 patient, with primary immunodeficiency as underlying clinical condition. Figure describes through time the changes in the evolutionary rates (by assuming an uncorrelated lognormal relaxed clock model), Ct values and SARS-CoV-2 treatments administered within the sampling window. For Halfmann-pt-1 the first viral sequence was obtained 113 days after the onset of symptoms. Patient was treated with multiple SARS-CoV-2 treatments within the sampling window. Colouring of the branches within the phylogenetic tree represents evolutionary rate estimates (in substitutions/site/year) obtained with BEAST2, lower values indicated with blue and higher rates with red colour. Open circles denote samples for which only Ct values were available and coloured circles denote samples which were sequenced.

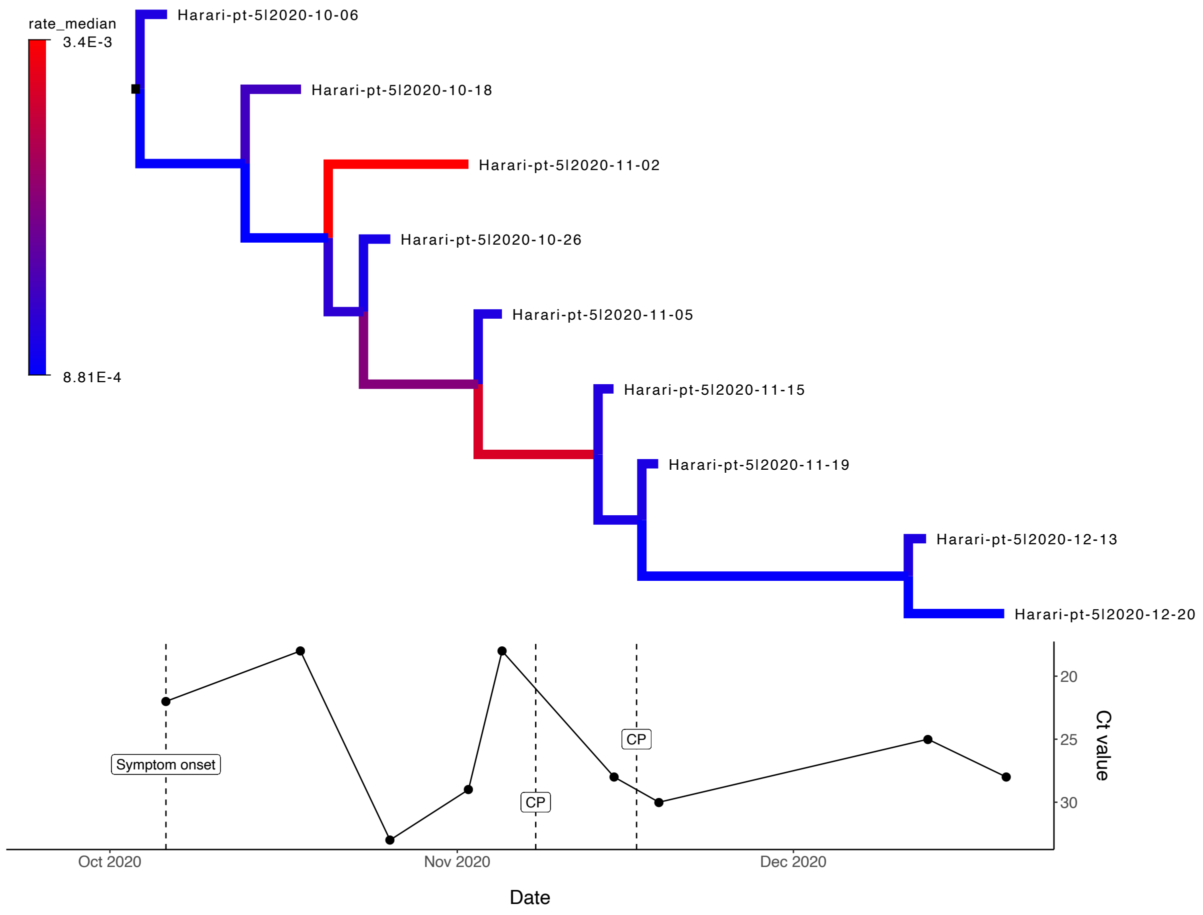

Supplementary figure S30. Patient case history for Harari-pt-5 patient, with acute lymphoblastic leukemia (ALL) as underlying clinical condition. Figure describes through time the changes in the evolutionary rates (by assuming an uncorrelated lognormal relaxed clock model), Ct values and SARS-CoV-2 treatments administered within the sampling window. For Harari-pt-5 the first viral sequence was obtained on the same day as the onset of symptoms. Patient was treated with convalescent plasma (CP) in total four times: on days 33 & 34 and 42 & 43 after the onset of symptoms. Convalescent plasma targets spike-protein and has a half-time of approximately 26 days with notable variation. Colouring of the branches within the phylogenetic tree represents evolutionary rate estimates (in substitutions/site/year) obtained with BEAST2, lower values indicated with blue and higher rates with red colour. Open circles denote samples for which only Ct values were available and coloured circles denote samples which were sequenced.

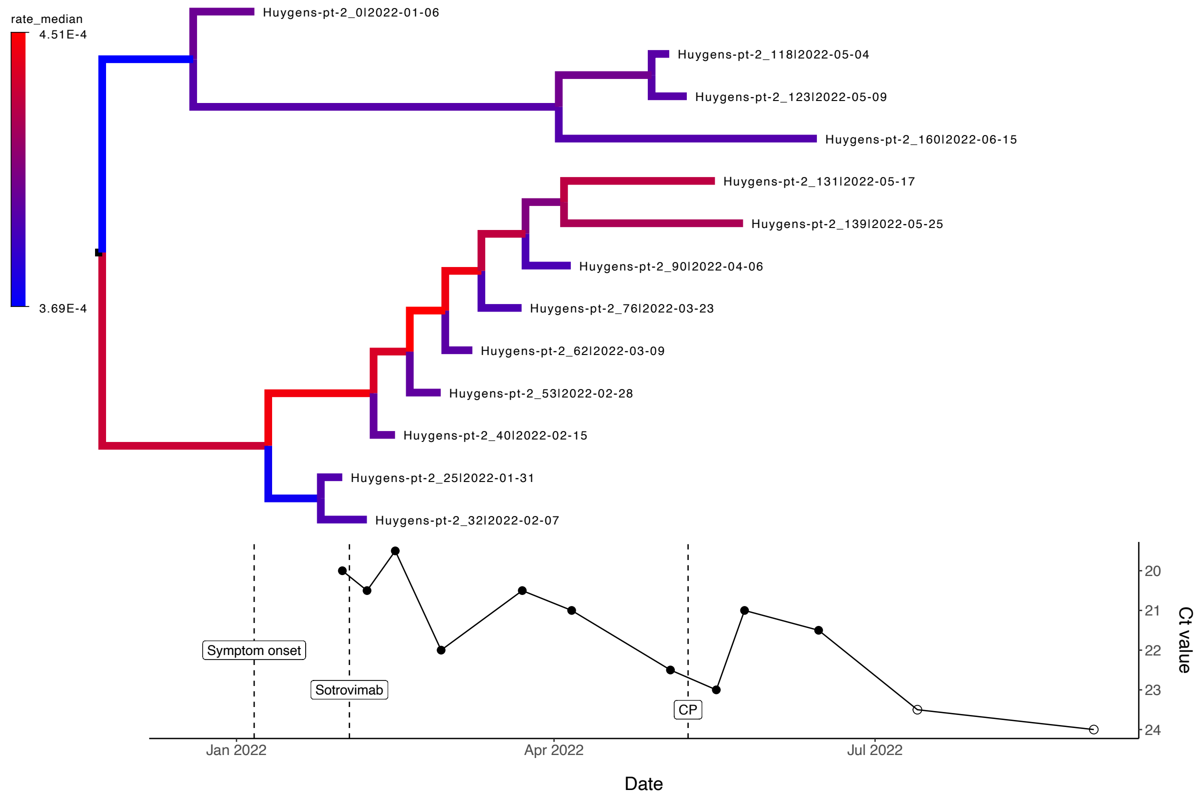

Supplementary figure S31. Patient case history for Huygens-pt-2 patient, with lymphoma as underlying clinical condition. Figure describes through time the changes in the evolutionary rates (by assuming an uncorrelated lognormal relaxed clock model), Ct values and SARS-CoV-2 treatments administered within the sampling window. For Huygens-pt-2 the first viral sequence was obtained on the same day as the onset of symptoms. Patient was treated with Sotrovimab, which targets the spike-protein and has a half-time of approximately 49 days. Additionally, the patient was treated with convalescent plasma (CP). Colouring of the branches within the phylogenetic tree represents evolutionary rate estimates (in substitutions/site/year) obtained with BEAST2, lower values indicated with blue and higher rates with red colour. Open circles denote samples for which only Ct values were available and coloured circles denote samples which were sequenced.

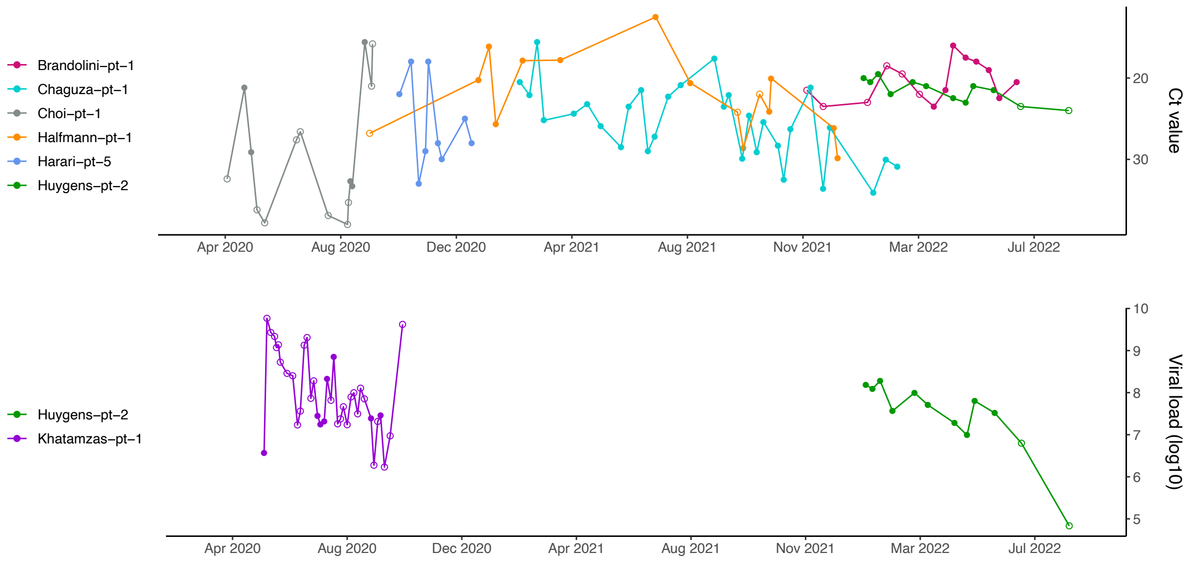

Supplementary figure S32. Ct values (upper panel) and viral load (lower panel) for seven of the datasets. Open circles denote samples for which only Ct values were available and coloured circles denote samples which were sequenced. For Huygens-pt-2 both Ct values and viral load estimates were available.

References

Alteri C, Cento V, Piralla A, Costabile V, Tallarita M, Colagrossi L, Renica S, Giardina F, Novazzi F, Gaiarsa S, et al. 2021. Genomic epidemiology of SARS-CoV-2 reveals multiple lineages and early spread of SARS-CoV-2 infections in Lombardy, Italy. *Nat. Commun.* 12:434.

Attwood SW, Hill SC, Aanensen DM, Connor TR, Pybus OG. 2022. Phylogenetic and phylodynamic approaches to understanding and combating the early SARS-CoV-2 pandemic. *Nat. Rev. Genet.* 23:547–562.

Baele G, Lemey P, Bedford T, Rambaut A, Suchard MA, Alekseyenko AV. 2012. Improving the accuracy of demographic and molecular clock model comparison while accommodating phylogenetic uncertainty. *Mol. Biol. Evol.* 29:2157–2167.

Bai Y, Jiang D, Lon JR, Chen X, Hu M, Lin S, Chen Z, Wang X, Meng Y, Du H. 2020. Comprehensive evolution and molecular characteristics of a large number of SARS-CoV-2 genomes reveal its epidemic trends. *Int. J. Infect. Dis.* 100:164–173.

Baum A, Fulton BO, Wloga E, Copin R, Pascal KE, Russo V, Giordano S, Lanza K, Negron N, Ni M, et al. 2020. Antibody cocktail to SARS-CoV-2 spike protein prevents rapid mutational escape seen with individual antibodies. *Science* 369:1014–1018.

Boskova V, Bonhoeffer S, Stadler T. 2014. Inference of Epidemiological Dynamics Based on Simulated Phylogenies Using Birth-Death and Coalescent Models. *PLOS Comput. Biol.* 10:e1003913.

Chaguza C, Hahn AM, Petrone ME, Zhou S, Ferguson D, Breban MI, Pham K, Peña-Hernández MA, Castaldi C, Hill V, et al. 2023. Accelerated SARS-CoV-2 intrahost evolution leading to distinct genotypes during chronic infection. *Cell Rep. Med.* 4:100943.

Didelot X, Croucher NJ, Bentley SD, Harris SR, Wilson DJ. 2018. Bayesian inference of ancestral dates on bacterial phylogenetic trees. *Nucleic Acids Res.* 46:e134.

Díez-Fuertes F, Iglesias-Caballero M, García-Pérez J, Monzón S, Jiménez P, Varona S, Cuesta I, Zaballos Á, Jiménez M, Checa L, et al. 2021. A Founder Effect Led Early SARS-CoV-2 Transmission in Spain. *J. Virol.* 95:e01583-20.

Douglas J, Mendes FK, Bouckaert R, Xie D, Jiménez-Silva CL, Swanepoel C, de Ligt J, Ren X, Storey M, Hadfield J, et al. 2021. Phylodynamics Reveals the Role of Human Travel and Contact Tracing in Controlling the First Wave of COVID-19 in Four Island Nations. *Virus Evol.*:1–18.

Drummond AJ, Bouckaert RR. 2015. Bayesian Evolutionary Analysis with BEAST. 1st ed. Cambridge University Press Available from: https://www.cambridge.org/core/product/identifier/9781139095112/type/book

Drummond AJ, Rambaut A, Shapiro B, Pybus OG. 2005. Bayesian Coalescent Inference of Past Population Dynamics from Molecular Sequences. *Mol. Biol. Evol.* 22:1185–1192.

Duchene S, Featherstone L, Haritopoulou-Sinanidou M, Rambaut A, Lemey P, Baele G. 2020. Temporal signal and the phylodynamic threshold of SARS-CoV-2. *Virus Evol.* 6:veaa061.

Fauver JR, Petrone ME, Hodcroft EB, Shioda K, Ehrlich HY, Watts AG, Vogels CBF, Brito AF, Alpert T, Muyombwe A, et al. 2020. Coast-to-Coast Spread of SARS-CoV-2 during the Early Epidemic in the United States. *Cell* 181:990-996.e5.

Galmiche S, Cortier T, Charmet T, Schaeffer L, Chény O, Platen C von, Lévy A, Martin S, Omar F, David C, et al. 2023. SARS-CoV-2 incubation period across variants of concern, individual factors, and circumstances of infection in France: a case series analysis from the ComCor study. *Lancet Microbe* 4:e409–e417.

Geidelberg L, Boyd O, Jorgensen D, Siveroni I, Nascimento FF, Johnson R, Ragonnet-Cronin M, Fu H, Wang H, Xi X, et al. 2021. Genomic epidemiology of a densely sampled COVID-19 outbreak in China. *Virus Evol.* 7:veaa102.

Ghafari M, Du Plessis L, Pybus OG, Katzourakis A. 2020. Time dependence of SARS-CoV-2 substitution rates. *Virological* [Internet]. Available from: https://virological.org/t/time-dependence-of-sars-cov-2-substitution-rates/542

Gray RR, Pybus OG, Salemi M. 2011. Measuring the temporal structure in serially sampled phylogenies: Temporal structure in phylogenies. *Methods Ecol. Evol.* 2:437–445.

Hill V, Du Plessis L, Peacock TP, Aggarwal D, Colquhoun R, Carabelli AM, Ellaby N, Gallagher E, Groves N, Jackson B, et al. 2022. The origins and molecular evolution of SARS-CoV-2 lineage B.1.1.7 in the UK. *Virus Evol.* 8:veac080.

Komissarov AB, Safina KR, Garushyants SK, Fadeev AV, Sergeeva MV, Ivanova AA, Danilenko DM, Lioznov D, Shneider OV, Shvyrev N, et al. 2021. Genomic epidemiology of the early stages of the SARS-CoV-2 outbreak in Russia. *Nat. Commun.* 12:1–43.

Lartillot N. 2023. Identifying the Best Approximating Model in Bayesian Phylogenetics: Bayes Factors, Cross-Validation or wAIC? *Syst. Biol.* 72:616–638.

Möller S, du Plessis L, Stadler T. 2018. Impact of the tree prior on estimating clock rates during epidemic outbreaks. *Proc. Natl. Acad. Sci. U. S. A.* 115:4200–4205.

Neher RA. 2022. Contributions of adaptation and purifying selection to SARS-CoV-2 evolution. *Virus Evol.* 8:veac113.

Norström MM, Prosperi MCF, Gray RR, Karlsson AC, Salemi M. 2012. PhyloTempo: A Set of R Scripts for Assessing and Visualizing Temporal Clustering in Genealogies Inferred from Serially Sampled Viral Sequences. *Evol. Bioinforma.* 8:261–269.

Pipes L, Wang H, Huelsenbeck JP, Nielsen R. 2021. Assessing Uncertainty in the Rooting of the SARS-CoV-2 Phylogeny.Malik H, editor. *Mol. Biol. Evol.* 38:1537–1543.

Seemann T, Lane CR, Sherry NL, Duchene S, Gonçalves da Silva A, Caly L, Sait M, Ballard SA, Horan K, Schultz MB, et al. 2020. Tracking the COVID-19 pandemic in Australia using genomics. *Nat. Commun.* 11:1–9.

Sigal A, Neher RA, Lessells RJ. 2024. The consequences of SARS-CoV-2 within-host persistence. *Nat. Rev. Microbiol.* [Internet]. Available from: https://www.nature.com/articles/s41579-024-01125-y

Smith MR. 2020. Information theoretic generalized Robinson–Foulds metrics for comparing phylogenetic trees. *Bioinformatics* 36:5007–5013.

To TH, Jung M, Lycett S, Gascuel O. 2016. Fast Dating Using Least-Squares Criteria and Algorithms. *Syst. Biol.* 65:82–97.

Volz EM, Frost SDW. 2014. Sampling through time and phylodynamic inference with coalescent and birth-death models. *J. R. Soc. Interface* 11:20140945.

Wolf JM, Wolf LM, Bello GL, Maccari JG, Nasi LA. 2023. Molecular evolution of SARS-CoV-2 from December 2019 to August 2022. *J. Med. Virol.* 95:e28366.
